# Synthesis, Characterization, and Evaluation of Palladium(II) Complexes Containing Chalcones on Gastric Adenocarcinoma Cells and *Helicobacter pylori*

**DOI:** 10.1021/acsomega.5c07262

**Published:** 2026-04-27

**Authors:** Jéssica Rodrigues Pereira de Oliveira Borlot, Rodrigo de Almeida Romagna, Ricardo Machado Kuster, Marcos Antônio Ribeiro, Priscilla Paiva Luz, Rita de Cássia Ribeiro Gonçalves, Reginaldo Bezerra dos Santos, Rodrigo Rezende Kitagawa

**Affiliations:** † Graduate Program of Chemistry, Exact Sciences Center, Federal University of Espirito Santo, Avenida Fernando Ferrari 514, Goiabeiras, Vitoria 29075-910, Espírito Santo, Brazil; ‡ Graduate Program of Pharmaceutical Sciences, Health Sciences Center, Federal University of Espirito Santo, Avenida Marechal Campos 1468, Bonfim, Vitoria 29047-105, Espírito Santo, Brazil; § Department of Chemistry, Exact Sciences Center, Federal University of Espirito Santo, Avenida Fernando Ferrari 514, Goiabeiras, Vitoria 29075-910, Espírito Santo, Brazil

## Abstract

Metal–chalcone complexes have emerged as promising scaffolds in bioinorganic medicinal chemistry; however, their potential as dual-acting agents targeting both gastric cancer and *Helicobacter pylori* infection remains insufficiently explored. In this study, eight palladium­(II) complexes containing chalcone-derived ligands were synthesized and characterized, corresponding to the molecular formulas C_30_H_22_Cl_4_O_4_Pd **(1C)**, C_38_H_24_Cl_2_O_4_Pd **(2C)**, C_38_H_28_Cl_2_O_4_Pd **(3C)**, C_40_H_30_O_6_Pd **(4C)**, C_30_H_24_Cl_2_O_4_Pd **(5C)**, C_30_H_20_F_2_O_4_Pd **(6C)**, C_32_H_26_O_6_Pd **(7C)**, and C_38_H_24_F_2_O_4_Pd **(8C)**. The complexes were evaluated for cytotoxic activity against human gastric adenocarcinoma cells (AGS), antibacterial activity against *H. pylori*, and inhibition of the urease enzyme. Structural characterization was carried out by using UV–vis and FTIR spectroscopy analyses, ^1^H NMR spectroscopy, high-resolution mass spectrometry, elemental analysis, and thermogravimetric analysis. In cytotoxicity assays, most palladium­(II) complexes exhibited enhanced activity and selectivity compared to their corresponding free ligands, with selected derivatives, including complexes **1C, 4C,** and **8C**, showing improved selectivity indices relative to cisplatin. Against *H. pylori*, all complexes displayed bactericidal activity stronger than that of the free ligands, with naphthol-based derivatives bearing methoxy or fluorine substituents showing the highest activity. In urease inhibition assays, several complexes were active, with phenolic derivatives exhibiting more pronounced effects; among them, complex **5C** showed the lowest IC_50_ value (9.72 μM). Molecular docking studies were employed as a qualitative and exploratory tool to rationalize trends observed in the in vitro assays, revealing consistent interaction patterns between the most active complexes and key catalytic residues of urease, including binding geometries compatible with coordination to the Ni^2+^ ions at the active site. The results demonstrate that coordination to palladium­(II) significantly modulates the biological profile of chalcone ligands, enhancing both cytotoxic and antibacterial activities. These findings highlight palladium–chalcone complexes as promising lead structures for the development of multifunctional agents targeting gastric cancer and *H. pylori*-associated diseases.

## Graphical abstract

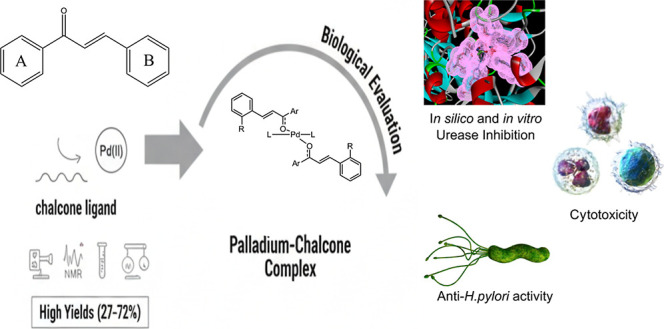

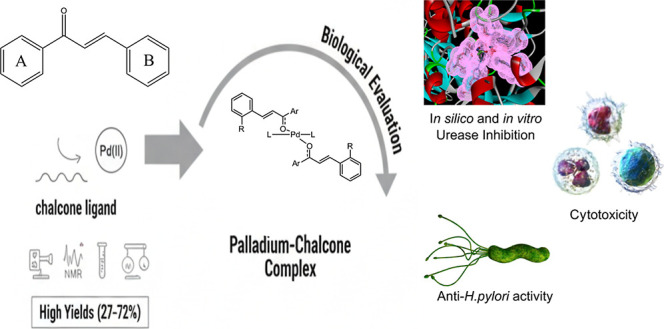

## 1 Introduction

Gastric cancer represents a significant global public health challenge as one of the most frequent and deadly malignant tumors. According to recent GLOBOCAN data, it ranks fifth in incidence among all cancer types and is among the four leading causes of cancer-related deaths worldwide, behind only lung, colorectal, and liver cancers.^1^ Its occurrence is complex and multifactorial; however, the most concerning aspect is that 75% of gastric cancer cases are due to infections by *Helicobacter pylori*.^2^

The outcome of an *H. pylori* infection may be atrophic gastritis, which can lead to gastric carcinoma, or it may remain localized and limited to duodenal ulcers. The factor that most influences this outcome is the presence of polymorphisms or mutations in the host’s genes, which regulate the intensity of inflammation in the gastric tissue and, therefore, affect the risk of specific clinical outcomes.^3^

*H. pylori* possesses characteristics that make it a potent carcinogenic agent. Initially, it acts as a typical pathogen, invading the host and triggering inflammation in target tissues, especially the gastric mucosa. However, what distinguishes it are its specific pathogenic mechanisms and potent virulence factors, which not only induce chronic inflammation but also directly damage the host cell DNA and activate pathways that favor cell survival. These processes are interconnected and often reinforce each other, resulting in a reduced DNA repair capacity in infected cells. This impairment contributes to increased genetic instability and accumulation of mutations, which can activate oncogenes and inactivate tumor suppressor genes, progressively increasing the risk of gastric cancer development.^4–6^

Thus, after colonizing the host’s stomach, *H. pylori* must overcome essential steps to maintain a persistent infection. First, the bacterium resists the acidic environment of the stomach thanks to the action of urease, which neutralizes the local pH and facilitates survival.^7^ Then, its motility, enabled by flagella, allows it to move toward the gastric mucosa.^8^ Adhesion to host cells is mediated by specific proteins such as BabA and SabA, which bind to receptors on epithelial cells.^9^ Finally, the release of CagA and VacA toxins promotes tissue damage and chronic inflammation—factors that contribute to infection pathogenesis and increase the risk of gastric cancer.^10^

To minimize the risk of gastric cancer, eradication of *H. pylori* is essential, which poses a challenge due to the growing antibiotic resistance of *H. pylori* to first-line drugs. Therefore, there is an urgent need to develop new and effective treatments against *H. pylori*.^11^

Natural products play a fundamental role in drug discovery and are valuable sources of information for the development of innovative therapies. Studies show that more than 50% of approved drugs are derived from or inspired by natural compounds, highlighting their historical and current importance in pharmacology.^12^ Moreover, the structural diversity and molecular complexity of natural products offer significant advantages in modulating complex therapeutic targets such as proteins and metabolic pathways associated with diseases.^13,14^

Among natural products, chalcones stand out as aromatic ketones, naturally occurring as the central scaffold of many bioactive compounds. They are structural precursors of flavonoids and isoflavonoids. The basic structure of chalcones consists of 1,3-diphenyl-2-propen-1-one, which occurs in *trans*- and cis-isomeric forms. The trans-isomer is thermodynamically more stable than the cis-isomer and is the most predominant configuration.^15–17^ The chemistry of chalcones continues to fascinate researchers in the 21st century due to the large number of substitutable hydrogens, which allows for the generation of a wide variety of derivatives and promising biological activities.^17,18^

One strategy for obtaining new derivatives is the application of coordination chemistry, which has emerged as a strategic area in the development of new therapeutic agents, particularly due to the ability of metal complexes to interact selectively with biomolecular targets. The versatility of transition metals, combined with the structural diversity of organic ligands, enables the construction of complexes with tunable physicochemical properties such as solubility, stability, and biological reactivity—features essential for pharmacological applications. Complexes containing metals, such as platinum, palladium, ruthenium, copper, and gold, have shown efficacy in treating various conditions, including cancer, microbial infections, and neurodegenerative diseases. Cisplatin, for instance, is one of the milestones of modern chemotherapy, and its discovery spurred the search for more selective and less toxic analogs.^19–21^

It is now widely recognized that many organic compounds used in medicine do not act through strictly organic mechanisms and that their activation or biotransformation depends entirely or partially on the presence of metal ions in the biological environment.^22,23^

Numerous studies have reported that natural and synthetic chalcones possess a wide range of pharmacological effects, modulating various molecular targets.^15,22,24,25^

Due to these properties, chalcone-based complexes have been increasingly investigated in bioinorganic medicinal chemistry owing to their chelating ability and coordination with various metals, showing modulatory effects on several targets. Chalcone-based complexes have been reported in multiple areas, including photophysics, catalysis, bioimaging, and especially in medical and pharmaceutical areas, demonstrating antitumor, antimicrobial, antimalarial, and antioxidant activities.

In one study, Travníček et al. (2024) synthesized copper­(II) chalcone complexes^26^ and evaluated their cytotoxicity against cancer cell lines such as ovarian (A2780), cisplatin-resistant ovarian (A2780R), prostate (PC3), osteosarcoma (HOS), breast (MCF7), lung (A549), and nonmalignant fibroblast cells (MRC-5). The complexes exhibited significant cytotoxicity against cancer cells in most cases, with IC_50_ values around 0.35–7.8 μM.

Campos et al. (2023) synthesized a Zn­(II) complex with chalcones and thiosemicarbazone and tested it against HTLV-1-infected leukemia cells (MT-2). They reported that the Zn­(II) complex was more cytotoxic than the free ligand, with IC_50_ values of 30.01 and 47.06 μM, respectively. In addition, the compounds exhibited pro-apoptotic effects without the release of reactive oxygen species (ROS) and with DNA interaction.^27^

Patange et al. (2011) tested in vitro antibacterial and antifungal activities of a series of methoxy- and nitro-substituted chalcones and their metal complexes, demonstrating that metal complexation influenced the activity, either enhancing or reducing it.^28^

Muskinja et al. (2016) synthesized ferrocenyl–chalcone complexes, which were tested for biological activity and showed relatively good in vitro antimicrobial activity against various bacterial and fungal strains.^29^

Therefore, the increasing incidence of resistant bacterial infections, along with the high mortality associated with gastric cancer, underscores the urgent need for new therapeutic agents with greater selectivity and efficacy than those of currently available molecules. In this context, there is strong evidence supporting the promising results of metal coordination with organic compounds. Thereby, we propose the synthesis and characterization of palladium­(II) metal complexes with chalcone-derived ligands, aiming to evaluate their effects against *H. pylori*—a key factor in gastric cancer pathogenesis—and analyze their cytotoxicity against gastric tumor and normal cells, to assess their therapeutic potential and selectivity.

## 2 Results and Discussion

### 2.1 Synthesis of Palladium Complexes Containing Chalcones

The complexes were synthesized from chalcone ligands that were synthesized according to Romagna et al. (2024).^30^ The yields are presented in Table 1.

**Table 1:** Yield of Palladium Complex

complex	chalcone	Ar	R	catalyst	yield(%)
1C	1	1	Cl	NaOH	57
2C	2	2	Cl		55
3C	3	2			30
4C	4	2	OMe		39
5C	5	1		NaOH	39
6C	6	1	F	NaOH	32
7C	7	1	OMe	NaOH	27
8C	8	2	F		72

During the synthesis of the palladium complexes (Scheme 1), different chalcones were used as ligands, with the main variation being the hydroxyl group present on one of the aromatic rings: some ligands contained a naphthol moiety, while others featured a phenol group. This structural difference had a direct impact on both the synthetic strategy employed and the yields obtained. For the chalcones with phenol groups, the use of sodium hydroxide as a catalyst was necessary to promote deprotonation of the hydroxyl group, thereby enabling coordination to the metal center. In contrast, chalcones containing a naphthol group exhibited greater intrinsic acidity, which facilitated complexation with palladium without the need for any additional catalyst.

**Scheme 1:**
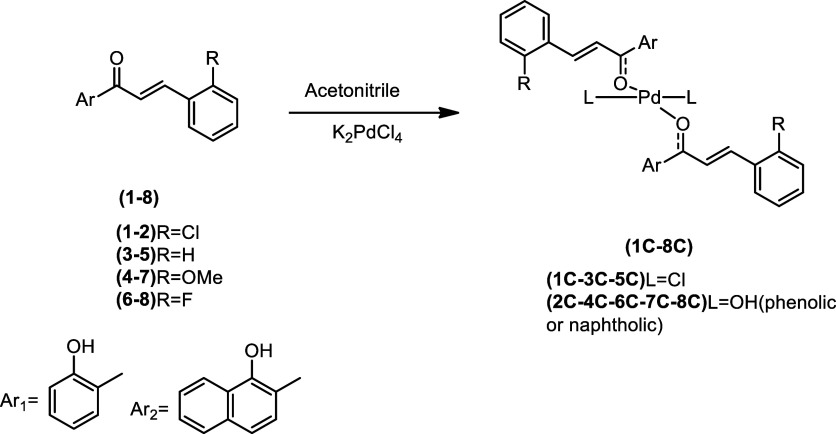
Synthesis of Palladium Complexes[Fn s1fn1]

a
**1–8**: chalcones (ligands) and **1C–8C**: palladium complexes with chalcones.

Analysis of the yields obtained for the eight synthesized complexes revealed significant variation, ranging from 27% to 72%. The products derived from naphthol-based chalcones—specifically complexes **2C**, **3C**, **4C**, and **8C**—generally showed higher yields. Notably, complexes **3C** (30%) and **8C** (72%) represent the extremes of this series. While complex **8C** was the most efficient, complex **3C** showed a lower yield, possibly due to structural differences in the formed product that may have limited the efficiency of coordination with the metal center. On the other hand, the complexes formed from chalcones containing a phenol group—namely, complexes **1C**, **5C**, **6C**, and **7C**—presented lower yields, between 27% and 57%, even with the use of sodium hydroxide as a catalyst. These results indicate that the presence of the naphthol moiety contributes to more efficient complexation with the metal center, likely due to its higher acidity and ease of coordination, highlighting the importance of the structural features of the ligand in the efficiency of metal complex synthesis.

### 2.2 Spectroscopic and Thermogravimetric Analysis of the Palladium Complexes

#### 2.2.1 FTIR Spectroscopic Data

Infrared spectroscopy (FTIR) was employed to investigate the coordination of the ligands to the palladium centers, focusing on the vibrational regions associated with the stretching of the conjugated C=O (1668–1645 cm^–1^),^31^ the C=C stretching (∼1602 cm^–1^),^32^ and the aromatic O–H absorptions (3500–3200 cm^–1^).^33^ This technique also enables the identification of changes in other characteristic vibrational modes, particularly those related to C–O and the conjugated aromatic system. The observed shifts in these bands indicate modifications in the vibrational profile upon complexation, providing relevant information about the structural changes resulting from the interaction with the metal. The main absorption values observed for the ligands and their corresponding palladium­(II) complexes are summarized in Table 2.

**Table 2:** Selected FTIR Bands (cm^–1^) and Their Assignments for Chalcone Ligands and Their Palladium Complexes

compound	ν(O–H)	ν(C–H) aromatic	ν(C = O) conjugated	ν(C = C) arom/conj	ν(C–O) (phenol/Ar–O)	γ(C–H) trans vinyl
1		3068	1638	1571, 1483, 1439	1202, 1154	973
1C		3058	1621	1606, 1549, 1510	1196, 1161	967
2	3422	3066, 3042	1631	1606, 1562	1144	
2C	3314	3055	1627	1573, 1467	1060	
3	3319	3053	1625	1612, 1510	1200	971
3C		3056	1631	1612, 1519	1245	971
4		3053	1623	1571	1196, 1148, 1062	745
4C		3045	1627	1608, 1558	1241, 1142	977, 745
5		3056, 3026	1636	1567	1199, 1151	975
5C	3348	3056, 3021	1618,1605	1536, 1605	1195	972
6		3042	1685	1636, 1560	1263	986
6C		3081	1618, 1605	1535, 1483	1193, 1137	
7	3151		1636, 1618	1583, 1518	1022	
7C	3077		1685	1638, 1569, 1486	1022	
8	3352	3042	1632, 1610	1559	1195	
8C		3057	1613, 1511, 1473	1511, 1473, 1394	1199	

The FTIR spectra of the ligands exhibited characteristic stretching IR absorption bands for the conjugated C=O group in the 1623–1685 cm^–1^ region. For instance, compound **1** displayed this band at 1638 cm^–1^ and compound **6** at 1685 cm^–1^. In the corresponding palladium­(II) complexes, the C=O stretching absorption was generally shifted to lower frequencies, such as 1621 cm^–1^ in **1C** and 1618/1605 cm^–1^ in **6C**, or appeared to be split into multiple components, as in **8C** (1613, 1511, and 1473 cm^–1^). These spectral changes clearly indicate modifications in the carbonyl vibrational environment upon coordination.

Broad stretching IR absorption bands for O–H were observed in all ligands between 3150 and 3420 cm^–1^, for example, at 3422 cm^–1^ in compound **2** and 3151 cm^–1^ in compound **7**. In the complexes, these absorptions were either shifted to lower frequencies, as in **7C** (3077 cm^–1^), or significantly attenuated, as in **3C** and **8C**. Additional variations were also recorded in the C–O stretching region (1020–1260 cm^–1^), such as the shift from 1144 cm^–1^ in compound **2** to 1060 cm^–1^ in **2C**, and in the C=C stretching region, where absorptions observed in the ligands were reorganized in the complexes (e.g., 1 at 1571/1483/1439 cm^–1^ compared to **1C** at 1606/1549/1510 cm^–1^). The γ­(C–H) *trans*-vinylic bending bands, typically found between 745 and 986 cm^–1^, also displayed shifts or reductions in intensity upon complexation, as seen in **1C** (967 cm^–1^) and **5C** (972 cm^–1^). Together, these results demonstrate systematic modifications in the vibrational profiles of the ligands upon formation of palladium­(II) complexes.

#### 2.2.2 UV–Vis Spectroscopy

The spectrophotometric analysis revealed significant spectral changes upon the complexation of the ligands with Pd­(II). While the free ligands exhibited intense π → π* bands, the complexes showed hypsochromic shifts along with the appearance of charge-transfer bands. These include LMCT (ligand-to-metal charge transfer), where electrons are donated from the ligand to the metal, and MLCT (metalla-to-ligand charge transfer), in which electrons are transferred from the metal to the ligand, reflecting the electronic interactions between the metal center and the coordinated ligands. The corresponding data are presented in Table 3.

**Table 3:** UV–Vis Absorption Bands (λmax, nm) of the Chalcone Ligands and Palladium­(II) Complexes Recorded in Acetonitrile

complex	ligand (λ_max_)	complex (λ_max_)	observation
1C	322	319, 455	increased intensity and MLCT
2C	373, 455	353, 428	hypsochromic and LMCT
3C	<350, 350–500	shifted bands	decreased intensity and hypsochromic
4C	330, 440	320, 410	hypsochromic without loss of intensity
5C	∼340	∼320	hypsochromic
6C	∼340	∼320	hypsochromic
7C	∼280, 370–390	∼310–330, 360–370	hypsochromic and MLCT
8C	325, 460	∼300, 325, 400	multiple bands and MLCT

#### 2.2.3 Proton Nuclear Magnetic Resonance Spectroscopy (^1^H NMR)

^1^H NMR spectroscopy was employed to investigate possible chemical shifts that could indicate coordination of the ligands to palladium­(II) complexes. In the free ligands, protons attached to sp^2^-hybridized carbons resonated between δ: 7.0 and 8.1 ppm, showing typical coupling patterns with coupling constants of approximately *J* ≈ 8.0 Hz for ortho-positioned protons and *J* ≈ 2.0 Hz for meta-coupling. The α and β protons adjacent to the carbonyl group appeared as doublets around δ: 8.0 ppm with *J* ≈ 15.0 Hz, confirming the E-configuration of the double bond. Particular attention was also given to the resonance peak assigned to the phenolic/naphtholic OH proton, which was detected in some cases as a highly deshielded singlet around δ: 12 ppm; however, in several samples, this signal was not clearly visible, most likely due to fast proton exchange and/or strong hydrogen-bonding interactions, which commonly broaden or suppress hydroxyl resonances in metal-coordinated systems. The NMR characterization of Pd­(II) complexes is intrinsically challenging due to factors such as reduced solubility, signal broadening caused by coordination effects, and possible dynamic behavior in solution. These factors may limit the acquisition of complete ^13^C and two-dimensional NMR data sets. In contrast, the Hα and Hβ protons were well resolved across the series, allowing a direct comparison between the ligands and their corresponding complexes. The chemical shift values obtained are summarized in Table 4.

**Table 4:** ^1^H NMR Chemical Shifts (δ, ppm) of the Chalcone Ligands and Their Corresponding Palladium­(II) Complexes, Recorded in Either CDCl_3_ or DMSO-*d*_6_ Depending on Solubility^a^

compound	δ(OH)	δ(Hα carbonyl)	δ(Hβ carbonyl)
1	12.24	8.06	8.12
1C	12.23	8.07	8.13
2	–	7.91	8.39
2C	–	8.25	8.25
3	–	7.73	8
3C	–	7.73	7.99
4	–	8.13	8.29
4C	–	7.29	7.46
5	–	7.42	7.57
5C	12.45	7.83	8.01
6	–	7.88	8.08
6C	-	7.91	8.07
7	–	7.98	8.15
7C	–	8.15	8.19
8	–	7.65	8.39
8C	–	8.04	8.22

a For each ligand–complex pair, spectra were acquired in the same solvent.

The ^1^H NMR spectra of the ligands and their corresponding palladium­(II) complexes revealed characteristic resonances associated with hydroxyl and vinylic protons. The resonance peak assigned to the phenolic/naphtholic OH proton, when detectable, appeared as a highly deshielded singlet around δ: 12 ppm, as observed for ligand **1** (δ: 12.24 ppm) and complex **5C** (δ: 12.45 ppm). In most cases, however, this signal was absent, which can be attributed to fast proton exchange or strong hydrogen-bonding interactions that broaden or suppress hydroxyl resonances, particularly in coordinating solvents.

The α and β vinylic protons adjacent to the carbonyl group were consistently observed as doublets with large coupling constants (*J* ≈ 15 Hz), confirming the E-configuration of the enone system. In the free ligands, Hα resonated within δ: 7.4–8.1 ppm and Hβ within δ: 7.5–8.4 ppm. Upon complexation, systematic variations were detected. For example, in ligand 2, Hα and Hβ appeared at δ: 7.91 and 8.39 ppm, respectively, whereas in **2C,** both signals coalesced at δ: 8.25 ppm. A more pronounced effect was observed for ligand **4**, where Hα and Hβ were recorded at δ: 8.13 and 8.29 ppm, respectively, but shifted upfield to δ: 7.29 and 7.46 ppm in 4C. Smaller downfield shifts were also noted in complexes such as **7C** and **8C** relative to their parent ligands.

Overall, the ^1^H NMR results indicate that the most consistent spectroscopic changes upon complexation are reflected in the chemical shifts of the α and β vinylic protons. The disappearance or attenuation of the OH resonance in several complexes, together with systematic variations in Hα and Hβ chemical shifts, demonstrates perturbations in the electronic environment of the α,β-unsaturated carbonyl framework upon coordination to palladium­(II).

It is important to consider that some spectra were recorded in DMSO-*d*_6_, a coordinating solvent known to interact with Pd­(II) centers and potentially promote dynamic equilibria in solution. Although complexes **1C** and **3C** exhibit aromatic regions resembling those of their corresponding free ligands, such a similarity does not necessarily imply decomplexation. O,O-chelated Pd­(II) systems commonly preserve the conjugated π-framework of the parent chalcone, resulting in comparable aromatic patterns. Importantly, no distinct additional set of resonances attributable exclusively to the free ligand was detected. Furthermore, high-resolution mass spectrometry independently confirmed the presence of intact palladium-containing molecular ions with isotopic distributions consistent with the proposed formulations. Therefore, while minor solution equilibria in a coordinating medium cannot be completely excluded, the combined NMR and HRMS data do not support extensive ligand dissociation under the experimental conditions.

#### 2.2.4 Mass Spectrometry

Mass spectrometry provided clear evidence of the molecular integrity and stoichiometry of the synthesized complexes (**1C**-**8C**). The spectra exhibited intense peaks corresponding to the protonated molecular ions [M + H]^+^, in excellent agreement with the theoretical monoisotopic masses calculated for each complex (Table 5) and exemplified in Figure 1 for compound **5C**. The deviations between experimental and theoretical *m*/*z* values were minimal (<0.5 Da), confirming the high precision of the measurements and validating the proposed molecular compositions.

**Table 5:** Major Signals’ Mass Spectrometry Data for Palladium Complexes

complex	chemical formula	*m*/*z* theoretical	*m*/*z* observed	error (ppm)	DBE	intensity (%)	assignment
1C	C_30_H_22_Cl_4_O_4_Pd	692.94	694.54	+1.60	18	48	[M + H]^+^
2C	C_38_H_24_Cl_2_O_4_Pd	721.02	721.08	+0.06	26	13.3	[M + H]^+^
3C	C_38_H_28_Cl_2_O_4_Pd	725.05	725.17	+0.12	24	19	[M + H]^+^
4C	C_40_H_30_O_6_Pd	713.12	713.44	+0.32	26	15.4	[M + H]^+^
5C	C_30_H_24_Cl_2_O_4_Pd	625.02	624.87	–0.15	17	18.75	[M + H]^+^
6C	C_30_H_20_F_2_O_4_Pd	589.04	588.91	–0.13	19	1	[M + H]^+^
7C	C_32_H_26_O_6_Pd	613.08	613.48	+0.40	19	4	[M + H]^+^
8C	C_38_H_24_F_2_O_4_Pd	689.08	689.16	+0.08	26	1	[M + H]^+^

**Figure 1:**
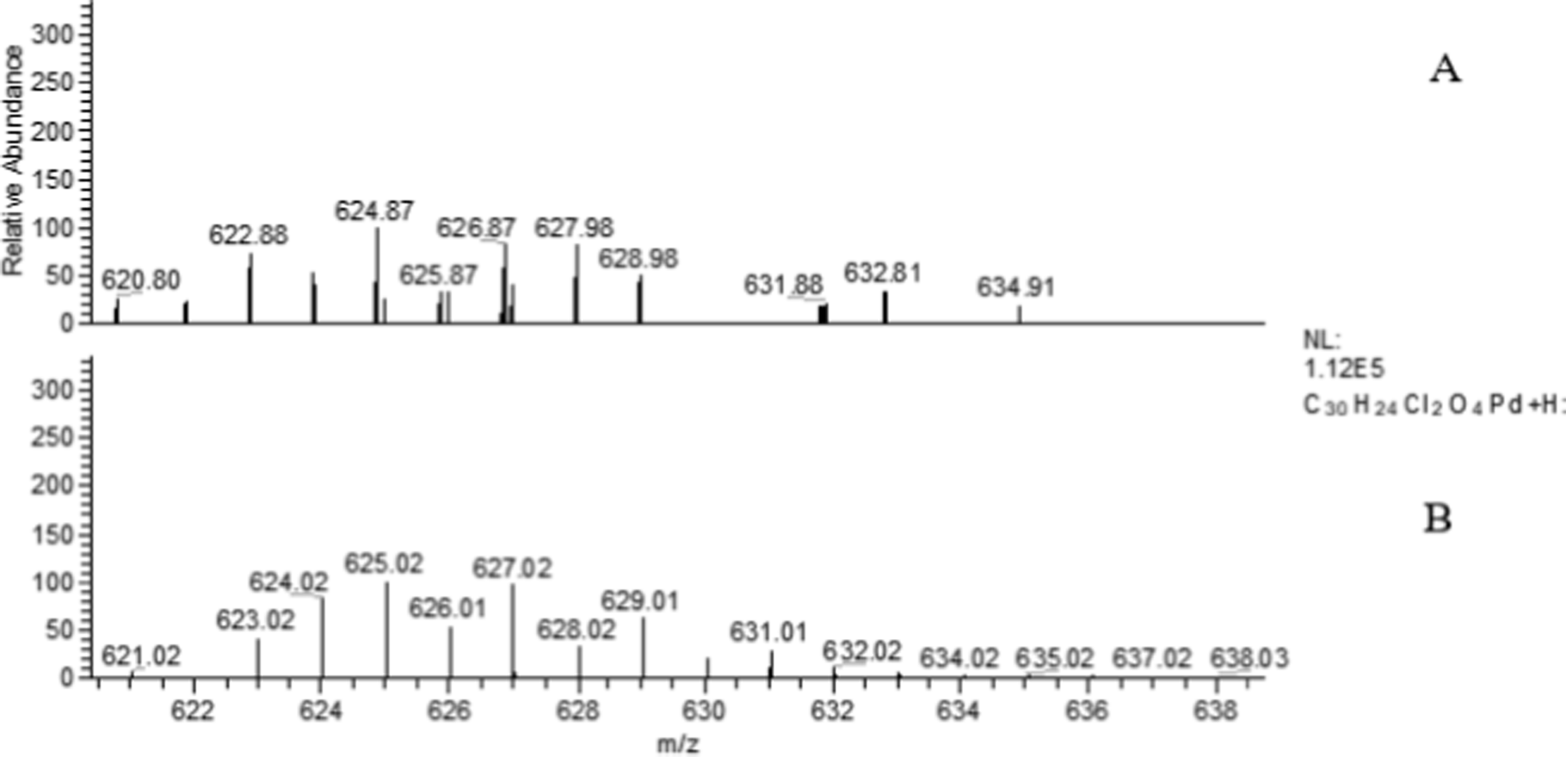
Comparison between the experimental (A) and theoretical (B) ESI–MS spectra of compound **5C** showing the [M + H]^+^ ion and the characteristic isotopic distribution pattern of palladium.

All spectra revealed the characteristic isotopic distribution arising from the natural isotopes of palladium (^106^Pd, ^108^Pd, and ^110^Pd), confirming the presence of the metal center within the molecular framework. The experimental isotopic envelopes closely matched the simulated theoretical patterns, supporting the elemental composition and homogeneity of the analyzed materials.

Minor fragment ions were also detected, associated with the loss of small functional groups and aromatic fragments. The low deviations observed between theoretical and experimental values (error < ± 3 ppm in most cases) confirm the accuracy of the data obtained, attesting to the reliability of the high-resolution spectra in the structural characterization of these complexes. Thus, the results support the formation of the coordinated species and validate the presence of the expected ligands, backing the other spectroscopic and structural evidence used in the elucidation of these palladium compounds.

#### 2.2.5 Thermogravimetric Analysis

Thermogravimetric analysis (TGA) was employed to evaluate the thermal behavior of the synthesized palladium­(II) complexes, allowing for the identification of decomposition steps, temperature ranges, and the corresponding percentages of mass loss associated with each thermal event. The initial (*m*_i_) and final (*m*_f_) masses of the samples were automatically recorded by a thermogravimetric analyzer. The percentages of mass loss (% Δ*m*) and residue (% *R*) were calculated according to the expressions

$$\%\;\Delta m=\left(m_{f}-m_{i}\right)/m_{i}\times 100$$

$$\%\;R=m_{f}/m_{i}\times 100$$

For comparison purposes, the theoretical mass losses were determined from the ratio between the molar masses of the eliminated fragments and that of the metal complex, according to the equation

$$\%\;\Delta M_{alc}=M_{fragment}/M_{complex}\times 100$$

where *M*_fragment_ corresponds to the molar mass of the eliminated group (e.g., HCl, HF, and organic fragments), and *M*_complex_ is the total molar mass of the compound.

By comparing the experimental and theoretical values, it was possible to identify the stages of solvent loss, elimination of volatile halogenated species (HCl, HF), degradation of the organic ligand, and the final oxidation of carbonaceous residues. The decomposition stages, temperature ranges, mass loss percentages, as well as the decomposition products, are presented in Table 6.

**Table 6:** Thermal Decomposition Stages (TG) of the Palladium Complexes and Final Residues

complex	main decomposition range (°C)	DTG max (°C)	total mass loss (%)	final residue
1C	150–500	226	87.1	PdO + C (10.8%)
2C	150–500	440	88.8	Pd/PdO (8.2%)
3C	150–400	304	88.8	Pd/PdO (8.3%)
4C	150–450	300	90.3	Pd/PdO (5.9%)
5C	150–500	256	69.9	PdO + C (29.2%)
6C	150–800	259	78.9	Metallic Pd (17.8%)
7C	150–400	276	78.3	Metallic Pd (17.7%)
8C	150–430		84.5	Metallic Pd (15.2%)

To illustrate this, the procedure for interpreting and calculating mass variations was applied to complex **7C**. The sample displayed typical thermal behavior for palladium­(II) complexes containing organic chalcone-type ligands, exhibiting four main stages of decomposition, as indicated in Table 6.

From the TG curve, it was observed that the initial mass (*m*_i_) of the sample was 2.147 mg, and the final mass (*m*_f_), obtained after 950 °C, was 0.380 mg. Thus, the total experimental mass loss (Δ*m*%) was calculated using the following equation:

%Δm=(2.147−0.380)/2.147×100=82.3%

This value is in excellent agreement with the expected behavior for the elimination of the organic fraction of the complex, indicating that approximately 17.7% of the initial mass remains as an inorganic residue at the end of thermal decomposition.

The molar mass of complex C_32_H_26_O_6_Pd is M = 612.97 g·mol^–1^, and the theoretical fraction corresponding to the metal was calculated by the ratio

$$\%\;{Pd}_{theor}=106.42/612.97\times 100=17.4\%$$

The obtained experimental value (17.7%) is in remarkable agreement with the theoretical value, confirming that the final residue consists of metallic palladium (Pd^0^), resulting from complete oxidation of the organic matrix.

The same calculation procedure was applied to all other complexes in the series, allowing a comparison of the thermal stability and nature of the residues among the phenolic, naphtholic, chlorinated, and fluorinated systems. They showed good thermal stability up to approximately 200 °C, with no significant mass losses, indicating the absence of coordinated solvent molecules or hydration. The decomposition of the ligands began between 200 and 250 °C, with the main degradation stages occurring up to about 400 °C, associated with the rupture of the conjugated π-system and progressive oxidation of the aromatic skeleton. This behavior is typical of coordination compounds containing carbonyl groups and conjugated unsaturated ligands to the metal center.

The phenolic core complexes (**5C**, **6C**, **1C**, and **7C**) exhibited well-defined decomposition profiles, with residues between 17% and 30%, consistent with the formation of Pd^0^, PdO, or PdO + C. Within this group, complex **6C** showed the highest thermal stability and a final residue close to the theoretical value, while the chlorinated systems (**5C** and **1C**) exhibited more pronounced mass losses and lower residues. Complex **7C**, containing methoxy groups, showed a more gradual decomposition, with residue consistent with the presence of metallic Pd, reflecting the stabilizing effect of electron-donating substituents.

On the other hand, the naphtholic core complexes (**2C**, **3C**, **4C**, and **8C**) showed smaller residues, ranging from 5% to 15%, indicating higher susceptibility to oxidation and volatilization of organic fragments. This behavior is consistent with the larger extent of the conjugated aromatic system, which, while favoring electronic delocalization, also intensifies oxidative degradation at high temperatures. The fluorinated systems (**8C**) maintained residues close to the theoretical values for metallic Pd, while the chlorinated systems (**3C** and **2C**) showed values below the theoretical ones, associated with the partial volatilization of PdO_*x*_ and PdCl_2_ in an oxidizing atmosphere.

Despite the variations, the overall results demonstrate that the complexes exhibit consistent thermal profiles and that the controlled decomposition of the ligands leads to the formation of residues predominantly composed of metallic palladium or PdO. Thus, the thermogravimetric analyses reinforce the thermal stability and structural robustness of the coordinated systems while also highlighting the influence of aromatic substituents on thermal resistance and decomposition modes.

In addition to the spectroscopic analyses, elemental analysis (CHN) of the synthesized palladium complexes was performed. While some complexes showed satisfactory agreement between experimental and theoretical carbon and hydrogen values, significant deviations were observed for chloride-rich derivatives. Such discrepancies are not uncommon in halogenated palladium­(II) complexes and may reflect solid-state instability, partial chloride lability, or heterogeneity associated with drying and storage processes. Elemental analysis of metal-containing compounds can be particularly sensitive to these factors due to the high relative mass contribution of heavy atoms such as Pd and Cl.

Importantly, high-resolution mass spectrometry (HRMS) unequivocally confirmed the proposed molecular formulations, including the characteristic isotopic distribution patterns of the palladium and chlorine atoms. The low parts per million errors and the agreement between calculated and observed isotopic envelopes strongly support the presence of the expected number of halogen atoms in each complex. Therefore, despite the deviations observed in some CHN values, the structural assignments remain supported by the combined spectroscopic, mass spectrometric, and thermogravimetric evidence.

The combined analysis of the different techniques employed allowed for a clear elucidation of the structural particularities of each Pd­(II) complex. In the case of **1C**, the shifts observed in the FTIR spectrum, together with the charge-transfer bands in the UV–vis spectrum and the [M + H]^+^ ion detected by mass spectrometry, confirmed its formation, further supported by thermal stability up to 500 °C and the consistency of HRMS and spectroscopic data. Complex **2C** displayed a broader coordination profile, with shifts in all FTIR bands, a hypsochromic shift accompanied by LMCT transitions, significant changes in the carbonyl protons in the NMR spectrum, and the presence of PdO as the final residue in the TGA. Taken together with the spectroscopic, mass spectrometric, and thermogravimetric data, these results reinforce the robustness of its structural assignment. Complex **3C** revealed preferential coordination through the C=O group, with lower spectral intensity in the UV–vis spectrum and no marked variations in the NMR spectrum; however, its identity was ensured by the [M + K]^+^ adduct, the metallic residue after decomposition, and the consistency of HRMS and thermal data. Complex **4C** presented shifts in all FTIR bands, hypsochromic shift without loss of intensity, perceptible changes in the NMR signals, and structural confirmation by mass spectrometry, in addition to thermal stability and structural integrity supported by complementary analytical techniques. Complex **5C** was characterized by the combination of C=O and C=C shifts, variations in carbonyl protons, presence of the [M + Na]^+^ adduct, and a distinctive thermal profile, whose residue was attributed to PdCl_2_, while remaining consistent with the overall analytical profile discussed above. Complex **6C**, although showing complete band shifts and a hypsochromic shift, did not exhibit significant changes in the NMR spectrum; nonetheless, its characterization was supported by the [M + K]^+^ adduct and the high thermal stability with Pd^0^ as the residue, despite the absence of elemental confirmation. Complex **7C**, in turn, presented a convergent set of evidence, with full shifts in FTIR and MLCT bands, marked changes in carbonyl protons, predominant [M + H]^+^ ion, stability up to 1000 °C, and elemental values within an acceptable experimental range considering the limitations discussed above. Finally, **8C** exhibited an equally complete profile, with shifts in all FTIR bands, multiple MLCT bands in UV–vis spectra, expressive changes in NMR spectra, the presence of the [M + K]^+^ adduct, stability up to 400 °C with the Pd^0^ residue, and composition consistent with the CHN analysis. Thus, although all complexes were validated by multiple techniques, each revealed subtle particularities in coordination, thermal stability, and electronic profile, reinforcing the solidity of the characterization while highlighting the structural nuances that differentiate the synthesized species.

Although single-crystal X-ray diffraction data were not obtained for these complexes, the structural assignments are supported by the combined spectroscopic evidence, including consistent shifts in the ^1^H NMR and FTIR spectra, elemental analysis, and mass spectrometry data. It is acknowledged that, in the absence of crystallographic confirmation, definitive structural determination remains limited. This is particularly relevant for complex **2C**, which exhibited unusual NMR signal coalescence, and for derivatives displaying multiple FTIR bands in the carbonyl region. Nevertheless, the convergence of independent analytical techniques supports the proposed coordination mode. Despite several crystallization attempts using different methods—including slow solvent evaporation, vapor diffusion, and gradual cooling—suitable single crystals could not be obtained. Therefore, structural proposals were supported by a consistent set of complementary techniques including FTIR analysis, UV–vis analysis, NMR, HRMS, CHN analysis, and thermogravimetric analysis.

Regarding solubility and stability under the experimental conditions, all palladium­(II) complexes showed good solubility in polar aprotic solvents, particularly dimethyl sulfoxide (DMSO) and acetonitrile, and partial solubility in ethanol. Notably, the complexes were readily and completely solubilized in DMSO, which was the solvent employed for the preparation of stock solutions in terms of cytotoxicity and anti-*H. pylori* assays. Under these conditions, no precipitation or visible degradation was observed during the time frame of the biological experiments, suggesting that the complexes remained sufficiently stable in solution to exert their biological effects. Although systematic stability studies under physiological conditions were not performed, these observations support the relevance and reliability of the in vitro assays conducted in this study.

### 2.3 Cytotoxicity in Human Gastric Adenocarcinoma Cells

Metal coordination to α,β-unsaturated systems, such as chalcones, is widely recognized for modulating pharmacological properties and biological responses. The introduction of a Pd­(II) center may influence parameters such as lipophilicity, stability, and cellular uptake, potentially enabling alternative interactions with biomolecules.^34,35^ Palladium­(II) complexes, due to their square-planar geometry and ligand-exchange properties, have been reported in the literature to interact with the nucleophilic sites of biomolecules, including sulfur- and nitrogen-containing residues.^36^ These interactions have been associated, in related systems, with effects such as oxidative stress modulation and apoptotic signaling. However, it should be emphasized that such mechanisms are proposed based on literature precedents and were not directly investigated in the present study.^37,38^

Coordination may also promote electronic redistribution within the conjugated chalcone system, reducing the electron density on the carbonyl group and enhancing the electrophilic character of the α,β-unsaturated double bond, thereby favoring Michael-type additions with nucleophilic biomolecules. This modification can contribute to the enhanced antiproliferative activity often observed for metal–chalcone derivatives.^39,40^ Furthermore, the presence of the metal center may influence the generation of ROS and intracellular redox balance, which are the factors involved in apoptotic responses.^35,41^

To evaluate their antitumor potential, the palladium­(II) complexes were assessed against tumor cells (AGS) and nonmalignant L-929 fibroblast cells, and the IC_50_ values and selectivity indices (SI’s) are presented in Table 7. As shown, most palladium­(II) complexes exhibited enhanced cytotoxic activity compared with their corresponding free ligands.

**Table 7:** Cytotoxicity Data for Complexes, Ligands, and Cisplatin and Their Selectivity Index (SI)

substance	IC50 (ligand) (μM)		SI	IC50 (complex) (μM)		SI
	AGS	L-929		AGS	L-929	
**1C**	34.48 ± 1.96	63.70 ± 3.8	1.85	6.38 ± 0.6^a^	25.56 ± 1.2^a^	4.01^b^
**2C**	28.70 ± 4.39	303.67 ± 2.89	10.58	11.50 ± 0.9^a^	36.43 ± 1.3^a^	3.17^b^
**3C**	25.34 ± 0.13	95.55 ± 0.1	3.77	14.74 ± 0.9^a^	49.91 ± 1.5^a^	3.39^b^
**4C**	135.74 ± 3.76	43.18 ± 2.88	0.32	15.93 ± 0.8^a^	44.96 ± 1.5^a^	2.82^b^
**5C**	159.55 ± 2.64	404.55 ± 5.64	2.54	11.94 ± 0.9^a^	23.07 ± 1.0^a^	1.93^b^
**6C**	74.22 ± 5.52	175.40 ± 4.21	2.36	18.93 ± 1.0^a^	37.44 ± 1.2^a^	1.98^b^
**7C**	42.20 ± 0.04	120.03 ± 2.32	2.84	17.86 ± 1.0^a^	34.91 ± 1.3^a^	1.95^b^
**8C**	21.90 ± 0.8	105.92 ± 1.4	4.84	20.99 ± 1.1^a^	110.30 ± 1.8^a^	5.26^b^
**cisplatin**	15.06 ± 0.2	13.74 ± 1.8	0.61			0.61

a *p* < 0.0001 compared to the IC_50_ value of cisplatin.

b *p* < 0.05 compared to the SI of cisplatin.

The palladium­(II) precursor salt K_2_PdCl_4_ was also evaluated as a control to assess whether the metal center alone could account for the observed biological effects. Under the same experimental conditions, K_2_PdCl_4_ did not exhibit significant biological activity and, therefore, did not influence the results obtained for the palladium­(II) complexes.

Within the evaluated series, coordination to the Pd­(II) ion is generally associated with lower IC_50_ values compared with those of the corresponding free ligands, particularly against the AGS cell line. This observation suggests that complexation to the metal center modulates the cytotoxic profile of the compounds; however, no direct mechanistic relationship can be established based solely on the data presented.

Regarding the selectivity indices (SI’s), variable behavior was observed among the compounds. Complex **1C** showed a considerable increase in SI (from 1.85 to 4.01), whereas **2C, 5C, 6C,** and **7C** exhibited lower values compared to their corresponding ligands. Nevertheless, all Pd­(II) derivatives displayed SI values higher than that of cisplatin (SI = 0.61), suggesting relatively lower cytotoxicity toward the nontumor L-929 fibroblast line.

The derivatives **1C, 2C, 3C, 4C,** and **5C** presented IC_50_ values lower than those of cisplatin (15.06 μM). Complex **1C**, bearing a phenolic nucleus with a chloro-substituent, exhibited an IC_50_ value of 6.38 μM, reflecting a high cytotoxic activity. The **2C** complex, containing a chlorinated naphtholic core, showed an IC_50_ value of 11.50 μM, while its unsubstituted naphtholic analogue **3C** had an IC_50_ value of 14.74 μM. This difference may be related to the inductive effect of the chlorine atom, which tends to increase the electrophilicity of the conjugated system, facilitating interactions with biological targets.^34,37^

Complex **5C**, which features a phenolic core without substituents, exhibited an IC_50_ value of 11.94 μM, also lower than that of cisplatin, suggesting that metal coordination alone may favor an enhancement of the cytotoxic activity. The **4C** derivative, bearing a methoxy group (−OCH_3_), showed an IC_50_ value similar to that of cisplatin (15.93 μM) but a higher SI (2.82), possibly due to the electron-donating (+M) nature of the substituent, which can influence the electronic density of the aromatic system.

A comparison between complexes **3C** (naphtholic, unsubstituted) and **5C** (phenolic, unsubstituted) highlights differences primarily related to the aromatic core. Complex **5C** exhibited a lower IC_50_ (11.94 μM) and thus higher potency, while **3C** showed greater selectivity (SI = 3.39 vs 1.93 for **5C**). These differences indicate that the nature of the aromatic core may influence the balance between the cytotoxicity and selectivity within this specific series of compounds. Nevertheless, any mechanistic attribution related to specific molecular interactions should be regarded as speculative and requires further experimental validation.

Complexes **6C, 7C,** and **8C**, which contain fluorinated substituents or extended conjugated systems, presented intermediate IC_50_ values (17–21 μM) but maintained SI values higher than those of cisplatin. Complex **8C** displayed the highest SI (5.26) among the series, which may be related to a balance between lipophilicity and polarity that favors selective accumulation in tumor tissues.^35,41^

In some cases, coordination led to a reduction in selectivity index values, which may be related to nonspecific mechanisms of cytotoxicity. The higher lability of Pd­(II) compared to Pt­(II) can facilitate ligand-exchange reactions with thiol-containing biomolecules, such as glutathione and cysteine, promoting the formation of ROS and diffuse oxidative damage.^34^ Changes in overall lipophilicity may also enhance passive diffusion into normal cells, thereby reducing selectivity. Another possible factor is nonspecific binding to serum proteins, such as albumin, which can affect biodistribution and reduce preferential accumulation in tumor sites.^35^ Additionally, coordination to the metal may partially decrease the electrophilicity of the α,β-unsaturated system, attenuating its specific reactivity toward tumor-associated biomolecular targets.^38,42^

Overall, the variations observed among the compounds suggest that both the nature of the substituents and the type of aromatic core influence the cytotoxic and selectivity profiles within the evaluated compound set. Certain trends can be noted, such as differences in the activity between halogenated derivatives and those bearing electron-donating groups. However, these observations should be interpreted with caution as they are based on a limited number of compounds and do not allow the establishment of generalized structure–activity relationships or definitive mechanistic conclusions.

### 2.4 Antibacterial Activity Against *H. pylori*

Given the observed cytotoxic activities, tests were carried out against *H. pylori*, considering that the bacterium is present in approximately 75% of gastric adenocarcinoma cases. The results are presented in Table 8, where the complexes exhibited significant activity against *H. pylori*. Moreover, both the ligands and their respective complexes demonstrated bactericidal activity against the bacterium.

**Table 8:** Anti-*H. pylori* Activity of Ligands and Complexes Determined by MIC and MBC

substance	(ligand)				(complex)			
	MIC (μg/mL)	MIC (μM)	MBC (μg/mL)	MBC (μM)	MIC (μg/mL)	MIC (μM)	MBC (μg/mL)	MBC (μM)
1C	2	7.73	2	7.73	1	1.44	1	1.44
2C	8	25.91	8	25.91	4	5.54	4	5.54
3C	4	14.58	8	29.16	4	5.51	4	5.51
4C	8	26.29	16	52.57	1	1.4	2	2.8
5C	16	71.35	16	71.35	4	6.39	4	6.39
6C	4	16.51	8	33.02	2	3.4	2	3.4
7C	4	15.73	4	15.73	4	6.53	4	6.53
8C	2	6.84	2	6.84	1	1.45	8	11.61
amoxicillin	0.0625	0.17	0.125	0.34				

The authors have reported that metal ions exhibit unique antibacterial mechanisms and can interfere with the metabolic activity of *H. pylori* by altering the membrane permeability or promoting the production of ROS.^43–45^ As an effective strategy, anti-*H. pylori* mechanisms mainly involve metal ion release, ROS generation, and disruption of the bacterial cell membrane and biofilm.

Furthermore, metal complexes stand out as promising alternatives to organic compounds due to their steric and electronic properties, which enable mechanisms such as redox processes and electron transfer. Their tendency to form soluble positive ions with high affinity for electron-rich biomolecules, such as DNA and proteins, supports their potential as antimicrobial agents.^46,47^

Camargo et al. synthesized a silver-ion-based complex, demonstrating high efficacy against both planktonic and biofilm-associated *H. pylori*, with no in vivo toxicity in *Galleria mellonella*.^48^ Zhang et al. used palladium in the nanoparticle form and observed that Pd nanoparticles can absorb and release hydrogen, altering the bacterial cell membrane permeability and interfering with *H. pylori* metabolism. Furthermore, their material not only eradicated *H. pylori* but also inhibited the inflammatory response and repaired damaged gastric mucosa by suppressing the secretion of inflammatory factors in macrophages and removing ROS.^49^

The evaluation of anti-*H. pylori* activity for the ligands and their corresponding palladium complexes, expressed in terms of minimum inhibitory concentration (MIC) and minimum bactericidal concentration (MBC), clearly demonstrates the positive impact of metal coordination on antimicrobial potency.

In general, all complexes showed significantly lower MIC and MBC values compared with their free ligands. For example, complex **1C** reduced the MIC from 7.73 μM (2 μg/mL) (ligand) to 1.44 μM (1 μg/mL) (complex), representing a potency increase of approximately 5.4-fold. Complex **2C** showed a reduction from 25.91 μM (8 μg/mL) to 5.54 μM (4 μg/mL), and complex **3C** decreased from 14.58 μM (4 μg/mL) to 5.51 μM (4 μg/mL). Complex **4C** stood out with one of the most pronounced reductions—from 26.29 μM (8 μg/mL) to 1.40 μM (1 μg/mL), reflecting a nearly 19-fold increase in potency.

Similar trends were observed for complexes **5C** and **6C**: The MIC value decreased from 71.35 μM (16 μg/mL) to 6.39 μM (4 μg/mL) in complex **5C**, and from 16.51 μM (4 μg/mL) to 3.40 μM (2 μg/mL) in complex **6C**, indicating substantial improvements in antimicrobial activity. Complex **7C** showed reduced MIC value from 15.73 μM (4 μg/mL) to 6.53 μM (4 μg/mL), while complex **8C** presented a MIC value of 1.45 μM (1 μg/mL) compared to 6.84 μM (2 μg/mL) for the ligand, again confirming the benefit of metal insertion.

MBC values followed the same trend, reinforcing the enhanced bactericidal capacity conferred by palladium, particularly for complexes **1C**, **4C**, and **8C**, which exhibited the lowest MBC values (in μM).

To better understand these findings, the results were analyzed in relation to the aromatic core of the ligands—phenol or naphthol—as well as the nature of the substituent to evaluate their influence on antimicrobial activity. It was observed that complexes containing naphthol-based ligands (complexes **2C**, **3C**, **4C**, and **8C**) generally exhibited greater antibacterial activity, especially when combined with strong electron-donating or electron-withdrawing groups. Complex **4C**, which combines a naphtholic core with a methoxy group, and complex **8C**, with naphthol and fluorine, displayed the lowest MIC values [1.40 μM (1 μg/mL) and 1.45 μM (1 μg/mL)], respectively. This suggests that expanding the aromatic system, along with substituents that modulate electron density, enhances antimicrobial activity.

Complex **2C**, which also has a naphthol core but is substituted with chlorine, showed a MIC of 5.54 μM (4 μg/mL)—very similar to complex **3**, which lacks any substituent MIC = 5.51 μM (2 μg/mL)—suggesting that chlorine alone has a limited effect on activity in naphthol-containing systems.

Among the phenolic complexes (complexes **1C**, **5C**, **6C**, and **7C**), the presence and type of substituent also clearly influenced activity. Complex **1C**, containing chlorine on the phenolic ring, showed a MIC of 1.44 μM (1 μg/mL), substantially lower than complex **5C**, which has an unsubstituted phenolic core [MIC = 6.39 μM (4 μg/mL)]. This indicates that chlorine significantly enhances activity in phenol-based systems, contrary to its modest effect in naphthol. Complex **6C** (phenol with fluorine) also exhibited good activity [MIC = 3.40 μM (2 μg/mL), highlighting the favorable role of electron-withdrawing groups in promoting bactericidal effects. Conversely, complex **7C**, with a methoxy-substituted phenolic core, demonstrated weaker activity [MIC = 6.53 μM (4 μg/mL), suggesting that electron-donating groups like methoxy are less effective in phenol-based systems, possibly due to reduced conjugation and resonance effects compared to naphthol.

Overall, the results indicate that the combination of an expanded naphtholic system with substituents such as fluorine or methoxy is the most promising for maximizing activity against *H. pylori*. In phenol-based systems, electron-withdrawing groups (e.g., chlorine and fluorine) outperformed electron-donating ones. While unsubstituted cores were also active, they showed reduced potency, as seen with complex **5C** (phenol) and complex **3C** (naphthol). These findings highlight the potential of appropriate substituents to fine-tune polarity, electronic properties, and biological interactions, reinforcing the value of structure–activity relationship (SAR) studies in the rational design of novel palladium complexes as promising antimicrobial agents.

In this context, the clinical relevance of the results becomes evident as several complexes exhibited significant activity against *H. pylori*, with MIC and MBC values in the micromolar range, particularly for **1C**, **4C**, **6C**, and **8C**. These results are noteworthy given the increasing resistance of *H. pylori* to β-lactam antibiotics, including amoxicillin,^50^ and suggest that these compounds may act through complementary mechanisms such as urease inhibition and oxidative stress induction, thereby reducing the likelihood of cross-resistance. Thus, the results support the clinical potential of these complexes as promising alternatives for the treatment of gastric infections.

### 2.5 Evaluation of Activity on the Urease Enzyme

*H. pylori* can survive hostile pH conditions due to the production of large amounts of urease (6–10% of total protein), which converts urea into ammonia and carbamate, allowing the bacterium to persist in the acidic gastric environment by generating a neutral microenvironment.^51^ Therefore, due to its role in pathogenesis, urease is a key target in the search for agents to treat *H. pylori* infection. The deficiency of this enzyme can limit the bacterium’s ability to colonize the gastric environment.

To evaluate the inhibitory effects on urease, all of the synthesized compounds and their ligands were assessed. The detailed results are presented in Table 9.

**Table 9:** Inhibition Activity of Urease Enzyme by Ligands and Complexes

substance	IC50 (μM)	
	ligand	complex
1C	>500.00	33.29 ± 1.20
2C	>500.00	>500.00
3C	>500.00	>500.00
4C	>500.00	>500.00
5C	>500.00	9.72 ± 0.64
6C	>500.00	18.93 ± 1.20
7C	>500.00	71.58 ± 1.67
8C	>500.00	121.5 ± 1.4
acetohydroxamic acid	5.88 ± 0.91	

The results demonstrated that the free ligands were inactive against the enzyme, exhibiting IC_50_ values above the maximum tested concentration, indicating that the presence of the metal center is essential for biological activity.

Recent investigations suggest that the superior performance of metal complexes as urease inhibitors is associated with the electronic contribution of the coordinated metal center. Metal–ligand coordination can alter the charge distribution, stabilize reactive conformations, and enhance interactions with amino acid residues at the HPU active site. As a result, these complexes frequently surpass the inhibitory capacity of the uncoordinated ligands, since the metal center not only reinforces ligand binding but also interferes directly with the catalytic machinery of the enzyme.^52,53^

In this study, the palladium complexes exhibited urease inhibitory activity that appeared to be influenced by the type of substituents on the coordinated ligands. Among them, complex **5C**, derived from a phenolic ligand without additional substituents on the aromatic ring, showed the best inhibitory activity in the series, with an IC_50_ value of 9.72 μM. This observation suggests that the absence of bulky or strongly electron-modifying groups may allow a more favorable orientation of the hydroxyl group for interaction with the urease catalytic site, favoring the formation of hydrogen bonds with the active residues.

The introduction of electronegative substituents, such as fluorine (complex **6C**, IC_50_ = 18.93 μM) and chlorine (complex **1C**, IC_50_ = 33.29 μM), decreases the enzymatic activity when compared to complex **5C**. Although these groups can increase the acidity of the phenolic hydroxyl, they may also introduce steric hindrance or alter the electronic density of the aromatic system, thereby limiting molecular recognition at the active site. In this series, therefore, the addition of electronegative substituents did not enhance activity, suggesting the need for a careful balance between electronic and steric effects.

Complex **7C**, containing a methoxy group on the phenolic ring (an electron-donating group), exhibited a more modest activity (IC_50_ = 71.58 μM), indicating that donor groups may reduce hydroxyl acidity and disfavor interactions with urease. Additionally, steric bulk from the methoxy group could hinder optimal fitting into the active site.

For naphthol-based ligands, a trend of reduced or absent inhibitory activity was observed. Complexes **2C**, **3C**, and **4C** were inactive regardless of substituents, whereas complex **8C** (fluorinated naphthol) showed moderate activity (IC_50_ = 121.50 μM). These findings suggest that the steric hindrance and reduced conformational flexibility of the naphthol scaffold may limit proper accommodation in the urease active site and that the electronic redistribution of the hydroxyl group could decrease its acidity and ability to form hydrogen bonds with catalytic residues.

Taken together, these results indicate that the activity of the complexes depends on the substituent type and aromatic scaffold, but clear structure–activity relationships require further investigation. In particular, the comparisons suggest that, within this series, the simpler phenolic system without substituents (complex **5C**) offered more favorable interactions with the enzyme’s active site than its substituted analogues.

Although the complexes did not outperform acetohydroxamic acid (IC_50_ = 5.88 μM), used as a positive control, compounds **5C**, **6C**, and **1C** exhibited relevant activity and provided a structural basis for the development of urease inhibitors based on metal complexes. These results contribute to understanding how substituents influence activity and may guide future rational design of new molecules with optimized enzymatic interaction.

Urease inhibitors based on transition-metal complexes have attracted great interest because Ni^2+^ ions are found in the active site, and enzyme inhibition by metal compounds may be due to interactions of both the organic ligands and the transition-metal ions with the sulfhydryl group at the urease active center.^54^

Although acetohydroxamic acid is considered a classical and extensively studied urease inhibitor, its clinical application is limited by several factors. AHA shows low selectivity and stability, as it can form complexes with essential metal ions in biological systems, leading to significant toxicity, including hepatic and hematological effects.^55^ Additionally, the molecule undergoes rapid degradation under physiological conditions and exhibits low lipophilicity, which reduces its ability to cross biological membranes and reach the *H. pylori* infection site.^56^

These pharmacological and toxicological limitations justify the search for new classes of inhibitors, and metal complexes have emerged as promising alternatives. Coordination compounds, particularly those based on palladium­(II), can combine greater chemical stability and tunable lipophilicity with potential multiple mechanisms of action, such as urease inhibition via coordination with histidine or cysteine residues and direct antimicrobial effects.^57^ Thus, the observed results for complex **5C**, although slightly less potent than AHA, are notable, as they represent a promising starting point for the development of new urease inhibitors with improved selectivity, stability, and reduced toxicity.

### 2.6 Molecular Docking at the *H. pylori* Urease Active Site

Since urease is an essential enzyme for the survival of *H. pylori* in acidic environments, the search for effective inhibitors against this target has become a relevant focus in the development of new therapeutic approaches. In this context, molecular docking studies were employed as a qualitative and hypothesis-generating tool to explore possible interactions between the synthesized palladium complexes and the urease active site, allowing a rationalization of trends observed in the in vitro assays.

To evaluate interactions within the active site of *H. pylori* urease and to understand the potential interactions of the complexes, the binding modes of acetohydroxamic acid were investigated using molecular docking. The active site was defined based on the crystallographic complex deposited in the Protein Data Bank (PDB: 1E9Y43),^58^ with a 10 Å radius sphere centered on the crystallographic ligand. All scoring functions available in GOLD were tested, and ChemScore was selected as it best reproduced the ligand conformation. Ten runs were performed for each molecule, and the resulting docking poses were subsequently evaluated through visual inspection.

To inhibit the enzyme, acetohydroxamic acid forms four hydrogen bonds with amino acids Asp362, Gly279, Ala365, and His221. Additional interactions at the site involve coordination between the oxygen atom of the acid and the Ni atoms present in the enzyme’s active site, as reported by Shi et al.^59^ These Ni atoms interact with amino acids, assisting in the anchoring of the molecule to the active site.

A redocking study was performed to evaluate the docking software’s ability to reproduce the known bioactive conformation of acetohydroxamic acid, whose binding mode in the urease active site is well established.^58^ The poses obtained from docking were compared to the crystallographic conformation to determine the RMSD (rms deviation), a metric that illustrates the deviation from the experimental pose. The average RMSD obtained was 0.7607 Å, and the lowest RMSD found was 0.6528 Å (Figure 2), where values up to 2.0 Å are considered acceptable.^60^

**Figure 2:**
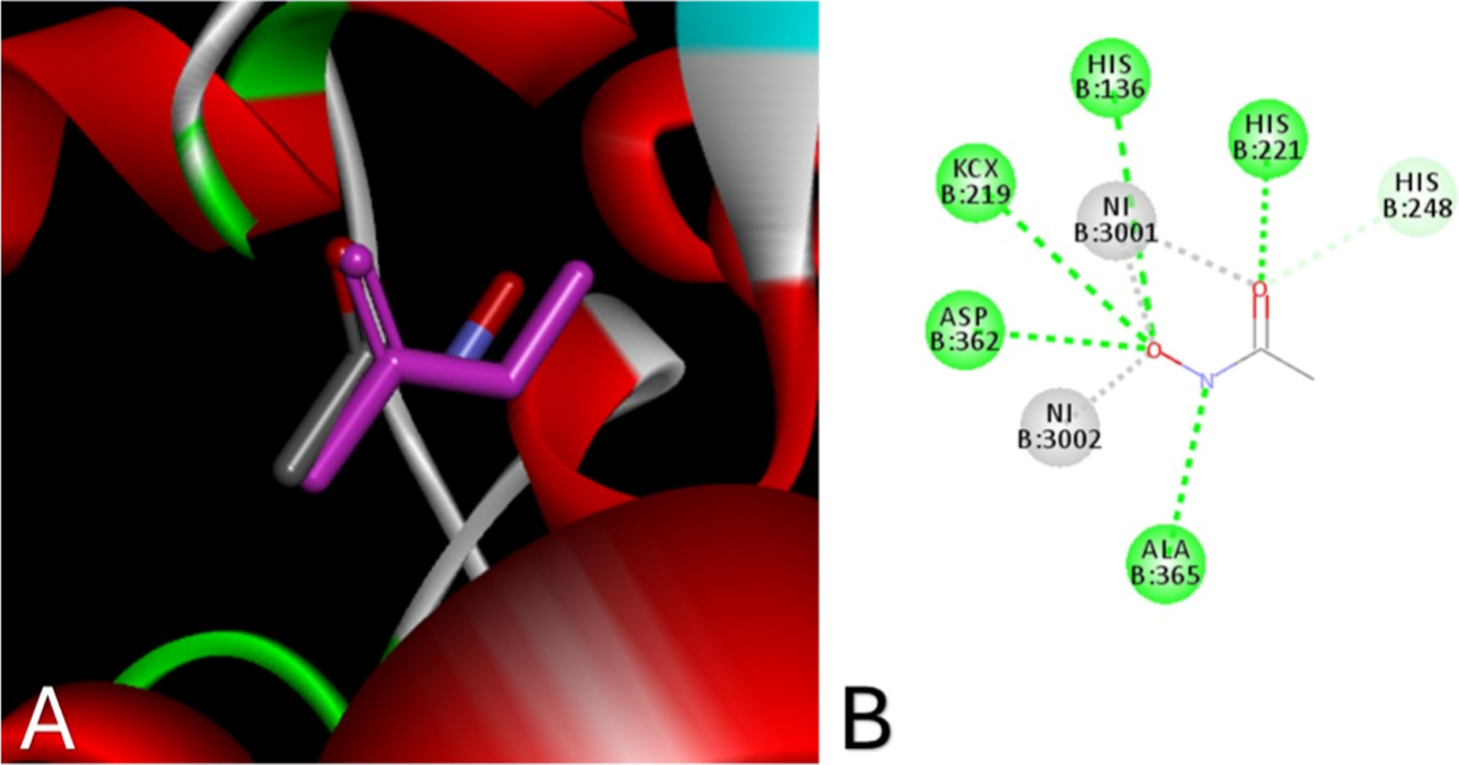
(A) Validation of the docking model through redocking (RMSD = 0.6528 Å) at the urease active site (PDB: 1E9Y). The reference ligand, acetohydroxamic acid, is shown with carbon atoms in gray, and the redocked pose is shown in purple. (B) Two-dimensional representation of the urease active site highlighting the main interactions of the crystallographic ligand acetohydroxamic acid. Note: hydrogen atoms are hidden.

Docking studies revealed that complexes **1C**, **5C**, **6C**, **7C**, and **8C**, which showed the lowest IC_50_ values in the biological assays, also exhibited key interactions with some of the catalytic residues of urease, such as Asp223, Gly279, Ala365, and His221—the same residues involved in the inhibition mechanism of acetohydroxamic acid, the reference inhibitor described by Shi et al. (2016). These complexes adopted binding poses oriented toward the Ni^2+^ ions in the active site, suggesting a potential for metal-centered interactions that may contribute to catalytic inhibition.

Complex **6C** was particularly noteworthy for mimicking the binding pattern of the reference inhibitor, forming interactions with Asp223, Gly279, Ala365, and His221, and showing proximity to the metal ions. This structural profile explains its notable in vitro performance with an IC_50_ value of 18.93 μM.

Complex **5C**, the most active of the series (IC_50_ = 9.72 μM), formed a hydrogen bond with Asp223 and established additional hydrophobic and π-stacking interactions with residues Trp224, His322, Cys321, Ala169, and Phe334. The combination of hydrophobic contacts and proper orientation within the catalytic pocket accounts for its high affinity and inhibitory efficiency.

Although complex **5C** exhibited inhibitory activity approaching that of acetohydroxamic acid under the same experimental conditions, no kinetic parameters (e.g., *K*_i_ values or inhibition mode) were determined in this study. Therefore, a direct mechanistic comparison with the reference inhibitor cannot be established at this stage.

Complex **1C**, with moderate activity (IC_50_ = 33.29 μM), exhibited a hydrogen bond with MET366, potentially contributing to binding stability along with various hydrophobic and van der Waals interactions, including interactions with key residues of the catalytic pocket such as His221, Ala365, and Gly279. The absence of coordination with Ni atoms and the weaker contacts with key catalytic residues explain the lower inhibitory potency observed in vitro.

Complexes **7C** and **8C** also yielded promising docking results consistent with their intermediate biological activities. Complex **7C** exhibited multiple interactions with important residues, such as Asp223, Ala365, Gly279, and His221, and additional contacts with Arg338 and His248, along with positioning close to the metal center. Complex **8C** showed a hydrogen bond with Cys321 and complementary interactions with Ala365, Arg338, Met366, Ala169, and Met317. These interactions support their experimentally observed intermediate inhibitory activity (IC_50_ values of 71.58 and 121.50 μM, respectively).

In contrast, complexes **2C**, **3C**, and **4C**, which were inactive in vitro, displayed only peripheral interactions in docking, lacking direct contact with Ni^2+^ ions and showing fewer interactions with critical catalytic residues. The absence of metal coordination in these cases aligns with the low efficacy observed in biological assays.

The lack of activity observed for these derivatives may be related not only to the absence of predicted metal-centered interactions but also to suboptimal orientation within the catalytic cavity, steric hindrance within the narrow access channel of urease, or insufficient stabilization by key catalytic residues. These factors may limit effective inhibition despite moderate docking scores.

As for the free ligands (chalcones), docking results confirmed their inability to coordinate directly with Ni^2+^ ions, compromising their potential as effective inhibitors. Although some chalcones, such as ligand 6, demonstrated favorable interactions with Ala365, Gly279, and even some proximity to the metal ions, this was not reflected in meaningful in vitro inhibition. These observations reinforce that complexation with palladium is crucial to achieving an appropriate binding geometry and functional urease inhibition.

The urease inhibition results should be interpreted as an initial screening assessment aimed at identifying promising candidates for further mechanistic investigation. Overall, the molecular docking results are consistent with the experimental trends, as the palladium­(II) complexes that exhibited lower IC_50_ values in vitro also showed more favorable interaction profiles within the urease active site. Complexes **5C, 6C**, and **7C** established interactions with key catalytic residues, including Asp223, Gly279, Ala365, and His221, and adopted binding orientations directed toward the Ni^2+^ ions present in the catalytic center. These observations provide qualitative insights into possible binding modes and offer a plausible rationale for the differences in inhibitory activity observed among the compounds.

However, molecular docking simulations are predictive in nature and do not constitute experimental proof of inhibition mechanisms. Therefore, complementary studies—such as enzyme kinetics, metal-binding investigations, or additional mechanistic assays—are required to validate the proposed inhibitory hypotheses. Taken together, the data suggest that complexes **5C, 6C**, and **7C** may represent promising lead structures within this series for further investigation rather than definitive urease inhibitors.

## 3 Conclusions

In this study, eight palladium­(II) complexes containing chalcone-derived ligands were successfully synthesized and characterized through complementary spectroscopic, thermal, and mass spectrometric techniques, confirming their structural integrity and coordination patterns. The presence of phenolic or naphtholic moieties and the nature of the substituents were found to significantly influence the synthetic yield, thermal stability, and spectroscopic behavior.

Biological assays demonstrated that complexation with Pd­(II) generally enhanced cytotoxic, antibacterial, and urease inhibitory activities compared to those of the free ligands. Several complexes exhibited IC_50_ values lower than that of cisplatin against AGS gastric carcinoma cells, with improved selectivity indices toward nonmalignant fibroblast cells. Moreover, all complexes showed a higher potency against *H. pylori* than their corresponding ligands, highlighting the favorable impact of metal coordination on antimicrobial activity. In urease inhibition assays, the phenolic derivatives—particularly complex **5C**—displayed the most promising results (IC_50_ = 9.72 μM), underscoring the importance of the hydroxyl group orientation and the absence of bulky substituents for optimal enzyme interaction.

Molecular docking studies provided mechanistic support for the biological findings, revealing that the most active complexes (**5C**, **6C**, and **7C**) interact with catalytic residues (Asp223, Gly279, Ala365, His221) and coordinate with Ni^2+^ ions at the enzyme’s active site—features considered essential for urease inhibition. The strong agreement between *silico* predictions and in vitro data confirms that both the aromatic framework and the metal coordination environment play critical roles in determining biological performance.

Taken together, the results highlight the potential of palladium–chalcone complexes as multifunctional agents with anticancer and anti-*H. pylori* activities. Their dual mechanism—combining direct bacterial inhibition with interference in urease activity—opens new perspectives for designing metal-based therapeutic agents targeting gastric cancer associated with *H. pylori* infection. Further mechanistic and in vivo studies are warranted to optimize selectivity, stability, and pharmacokinetic properties toward clinical application.

## 4 Materials and Methods

### 4.1 Chemistry

#### 4.1.1 Thin Layer Chromatography

Thin layer chromatography analyses were carried out using aluminum chromatoplates coated with silica gel UV 254 (250 μm, 20 × 20 cm). The samples were diluted in dichloromethane, applied to the plate using a glass capillary, and eluted with appropriate solvent mixtures. Visualization was performed under ultraviolet light (250 and 300 nm), and when necessary, a chemical developer (vanillin) was applied under heating.

#### 4.1.2 Ultraviolet–Visible (UV–Vis) Absorption Spectrophotometry

UV–vis absorbance spectra were obtained using a Biotek Epoch spectrophotometer in the range of 0–999 nm, with sample concentrations of 1 × 10^–5^ mol L^–1^ in acetonitrile.

#### 4.1.3 Infrared Spectroscopy

Infrared spectra were acquired by using a PerkinElmer Spectrum 400 spectrometer. All analyses were performed in Attenuated Total Reflectance (ATR) mode with a horizontal zinc selenide (ZnSe) crystal, averaging 16 scans at a resolution of 2 cm^–1^. The spectral window ranged from 4000 to 400 cm^–1^, within the mid-infrared (MIR) region.

#### 4.1.4 Proton Nuclear Magnetic Resonance (^1^H NMR) Spectroscopy

^1^H NMR spectra were recorded on a Varian 400 MHz spectrometer with a 5 mm Broadband ^1^H/X/D probe. Chemical shifts (δ) were reported in parts per million (ppm) relative to an internal standard. DMSO-*d*_6_ and CDCl_3_ were used as solvents, and tetramethylsilane (TMS) was used as the internal reference. The following conventions were adopted to describe signal multiplicity in ^1^H spectra: s (singlet), d (doublet), dd (doublet of doublets), ddd (doublet of doublets of doublets), t (triplet), dt (doublet of triplets), td (triplet of doublets), dtd (doublet of triplet of doublets), and m (multiplet).

#### 4.1.5 Mass Spectrometry

HRMS using direct infusion electrospray ionization (ESI) enabled the accurate characterization of the metal complexes. Samples were dissolved in 1 mL of methanol and analyzed in positive ion mode, ESI (+), by using an Orbitrap Exploris 120 mass spectrometer (Thermo Scientific, Bremen, Germany). Analyses were performed over an *m*/*z* range of 100–1000, with a resolution of 120,000 (*m*/*z* 200), spray voltage of 3500 V, source temperature of 320 °C, and vaporizer temperature of 275 °C. The sheath gas was set to 35 arbitrary units and the auxiliary gas to 7. Direct infusion was performed at a flow rate of 5 μL/min for 1 min. Spectra were processed using Xcalibur and MZmine,^61^ with ion assignment based on monoisotopic mass and formation of adducts such as [M + H]^+^, [M + Na]^+^, and [M + K]^+^, with mass errors below 10 ppm.

#### 4.1.6 Thermogravimetric Analysis

Measurements were carried out on a Shimadzu DTG-60H instrument with a nitrogen 5.0 flow rate of 50 mL/min and industrial oxygen at 50 mL/min. Samples were heated from room temperature to 1000 °C at a rate of 10 °C/min. Mass percentage calculations were performed using the following formula:

$$\Delta \;\%=\frac{MMproduct\;x\;100}{MMcompound}$$

#### 4.1.7 Elemental Analysis

The carbon, hydrogen, and nitrogen contents present in the samples sent with the medication were evaluated through elemental analysis on a PerkinElmer CHNSO model 2400 equipment.

### 4.2 Synthesis of Palladium Complex

In a 25 mL round-bottom flask equipped with a magnetic stir bar, the corresponding chalcone (0.50 mmol) was dissolved in 5.0 mL of acetonitrile under magnetic stirring. Depending on the nature of the chalcone, the reaction medium was previously activated with sodium hydroxide (NaOH, 0.50 mmol), and the mixture was stirred for approximately 5 min until a characteristic color change was observed. Subsequently, an aqueous solution of potassium tetrachloropalladate­(II) (K_2_PdCl_4_, 0.25 mmol) was added, and the resulting suspension was stirred continuously at room temperature for 48 h.

After completion of the reaction, the solid product was collected by vacuum filtration, washed successively with acetonitrile, ethanol, and water, and then dried under reduced pressure. The reaction schemes for obtaining the **1C**-**8C** complexes are shown sequentially (Schemes 2–9).

**Scheme 2:**
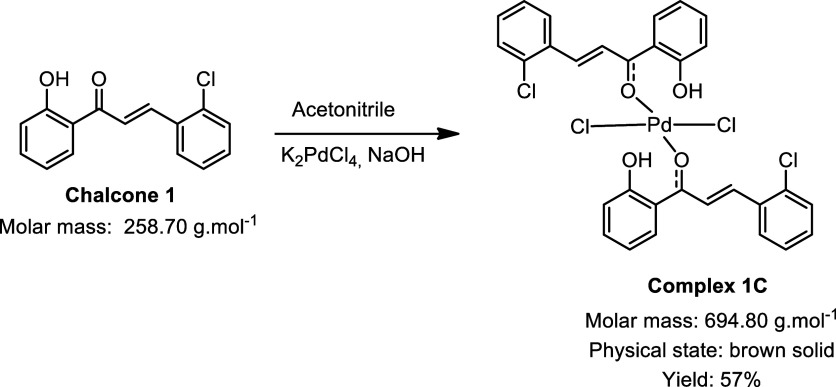
Synthesis of Palladium Complex **1C**

**3:**
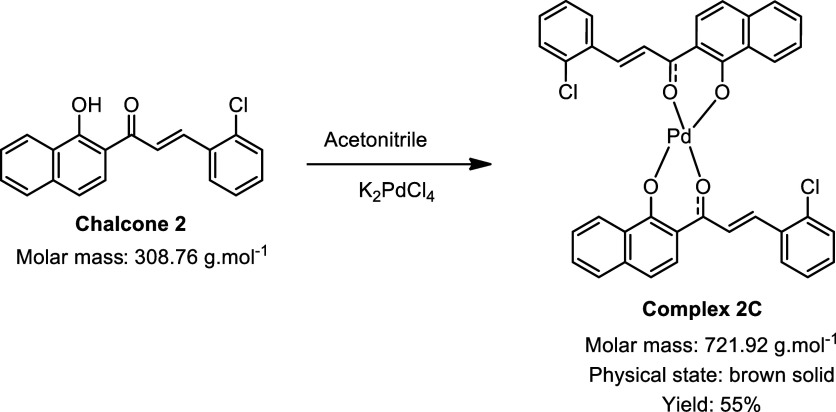
Synthesis of Palladium Complex **2C**

**4:**
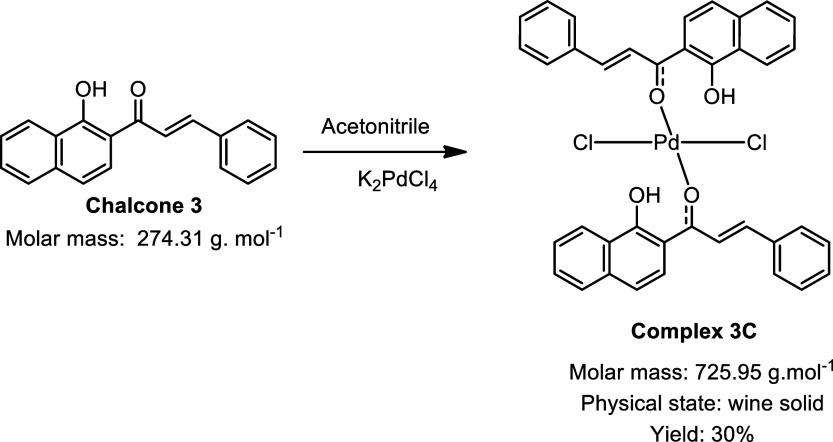
Synthesis of Palladium Complex **3C**

**5:**
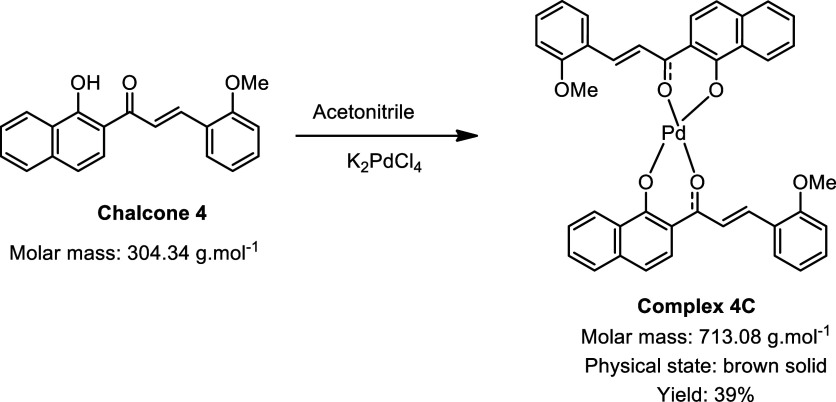
Synthesis of Palladium Complex **4C**

**6:**
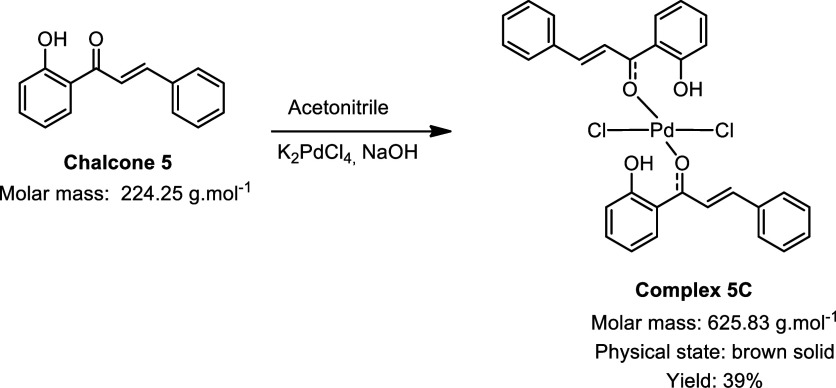
Synthesis of Palladium Complex **5C**

**7:**
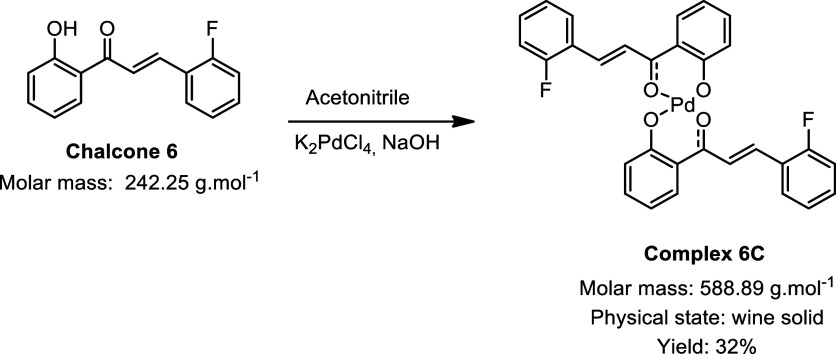
Synthesis of Palladium Complex **6C**

**8:**
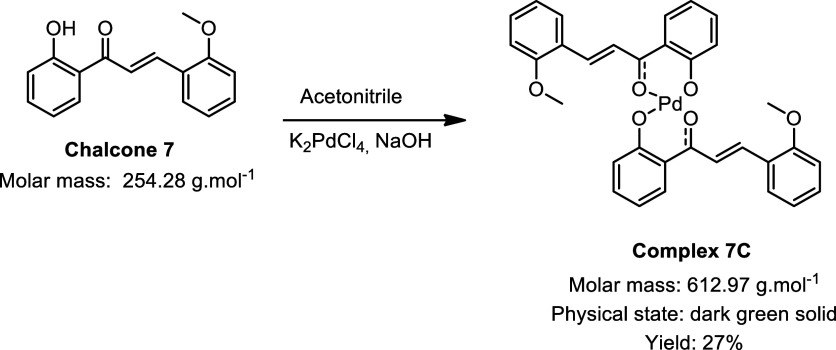
Synthesis of Palladium Complex **7C**

**Scheme 9:**
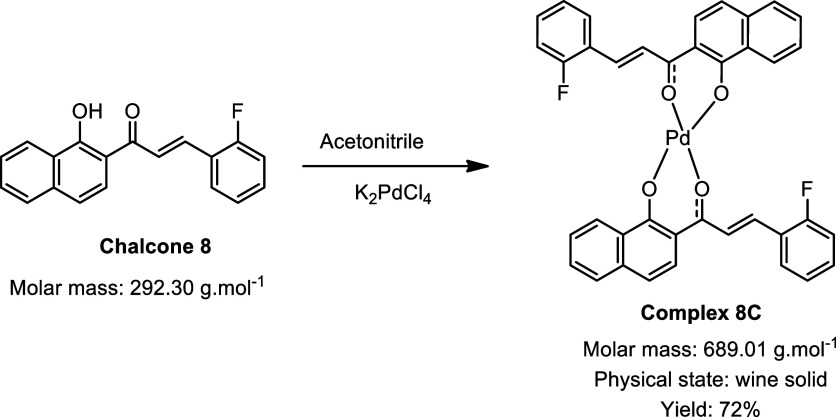
Synthesis of Palladium Complex **8C**

#### 4.2.1 Complex **1C**

**I.V. ν**_**max**_ (cm^–1^, ATR): 3055 (ν O–H); 1638 (ν C=O); 1572 (ν C=C).

##### 4.2.1.1 NMR de ^1^H

(400 MHz, DMSO-*d*_6_) δ: 12.23 (s, 1H), 8.26–8.19 (m, 2H), 8.13 (d, *J* = 15.6 Hz, 1H), 8.07 (d, *J* = 15.5 Hz, 1H), 7.62–7.56 (m, 2H), 7.53–7.46 (m, 2H), 7.05–7.00 (m, 2H), 3.33 (s, 8H), 2.51 (q, *J* = 1.9 Hz, 2H).

#### 4.2.2 Complex **2C**

**I.V. ν**_**max**_ (cm^–1^, ATR): 3057 (ν, O–H); 1625 (ν, C=O conjugated); 1572 (ν C=C).

##### 4.2.2.1 NMR de ^1^H

(400 MHz, DMSO-*d*_6_) δ: 8.38 (dd, *J* = 8.3, 1.3 Hz, 1H), 8.36–8.33 (m, 1H), 8.32 (d, *J* = 9.0 Hz, 1H), 8.25 (s, 1H), 7.95 (d, *J* = 8.1 Hz, 1H), 7.75 (ddd, *J* = 8.2, 6.9, 1.3 Hz, 1H), 7.65–7.58 (m, 2H), 7.54–7.46 (m, 3H), 3.32 (d, *J* = 2.0 Hz, 4H), 2.50 (p, *J* = 1.8 Hz, 2H).

#### 4.2.3 Complex **3C**

**I.V. νmax** (cm^–1^, ATR): 3054 (ν O–H); 1623 (ν C=O conjugated); 1610 (ν C=C).

##### 4.2.3.1 NMR de ^1^H

(400 MHz, CDCl3-d) δ: 8.51 (dq, *J* = 8.4, 0.7 Hz, 1H), 7.99 (d, *J* = 15.4 Hz, 1H), 7.86 (d, *J* = 8.9 Hz, 1H), 7.81–7.68 (m, 4H), 7.67–7.60 (m, 1H), 7.55 (ddd, *J* = 8.3, 6.9, 1.3 Hz, 1H), 7.46 (dt, *J* = 4.6, 3.1 Hz, 3H), 7.32 (d, *J* = 8.9 Hz, 1H).

#### 4.2.4 Complex **4C**

**I.V. νmax** (cm^–1^, ATR): 3078 (ν O–H); 1627 (ν C=O conjugated); 1601 (ν C=C).

##### 4.2.4.1 NMR de ^1^H

(400 MHz, DMSO-*d*_6_) δ: 7.59–7.52 (m, 1H), 7.50–7.42 (m, 1H), 7.35–7.25 (m, 1H), 7.12 (d, *J* = 8.2 Hz, 1H), 6.93–6.88 (m, 0H), 6.79 (ddd, *J* = 8.2, 6.8, 1.2 Hz, 1H), 6.71–6.61 (m, 1H), 6.33 (dd, *J* = 8.5, 1.0 Hz, 1H), 6.24 (d, *J* = 7.5 Hz, 0H), 3.12 (s, 1H).

#### 4.2.5 Complex **5C**

**I.V. νmax** (cm^–1^, ATR): 3021 (ν O–H); 1636 (ν C=O conjugated); 1568 (ν C=C).

##### 4.2.5.1 NMR de ^1^H

(400 MHz, DMSO-*d*_6_) δ: 8.26 (dd, *J* = 8.4, 1.7 Hz, 1H), 8.03 (d, 1H), 7.96–7.90 (m, 2H), 7.85 (d, *J* = 15.6 Hz, 1H), 7.53–7.46 (m, 3H), 7.05–6.98 (m, 2H).

#### 4.2.6 Complex **6C**

**I.V. νmax** (cm^–1^, ATR): 3080 (ν O–H); 1617 (ν C=O conjugated); 1605 (ν C=C).

##### 4.2.6.1 NMR de ^1^H

(400 MHz, DMSO-*d*_6_) δ: 8.19–8.11 (m, 1H), 8.07 (d, *J* = 15.8 Hz, 1H), 7.91 (d, *J* = 15.7 Hz, 1H), 7.87–7.81 (m, 1H), 7.62–7.52 (m, 2H), 7.21 (d, *J* = 7.4 Hz, 1H), 7.04–6.99 (m, 2H).

#### 4.2.7 Complex **7C**

**I.V. νmax** (cm^–1^, ATR): 3155 (ν O–H); 1636 (ν C=O conjugated); 1617 (ν C=C).

##### 4.2.7.1 NMR de ^1^H

(400 MHz, DMSO-*d*_6_) δ: 8.19 (dd, *J* = 8.4, 1.7 Hz, 1H), 8.15 (d, *J* = 15.7 Hz, 1H), 8.01–7.96 (m, 2H), 7.56 (ddd, *J* = 8.5, 7.2, 1.6 Hz, 1H), 7.50–7.45 (m, 1H), 7.13 (dd, *J* = 8.4, 1.0 Hz, 1H), 7.08–6.96 (m, 2H).

#### 4.2.8 Complex **8C**

**I.V. νmax** (cm^–1^, ATR): 3057 (ν O–H); 1624 (ν C=O conjugated); 1611 (ν C=C).

##### 4.2.8.1 NMR de ^1^H

(400 MHz, DMSO-*d*_6_) δ: 8.37 (d, *J* = 8.3 Hz, 1H), 8.27–8.24 (m, 1H), 8.24–8.19 (m, 2H), 8.04 (d, *J* = 15.6 Hz, 1H), 7.94 (d, *J* = 8.1 Hz, 1H), 7.76–7.71 (m, 1H), 7.64–7.44 (m, 4H), 7.37–7.30 (m, 2H).

### 4.3 Docking Studies

Molecular docking calculations were performed with GOLD software, v.5.5 2018.^62^ The crystallographic protein–ligand complex of urease and iNOS was obtained from the RCSB Protein Data Bank (PDB)^63^ under the codes: 1E9Y.^58^ The score function was set to CHEMSCORE.^64^ The grid region for docking was calculated over the coordinates of the central ligand, comprising a 10 Å radius sphere. The docking runs were set to 50. High-score solutions were saved for visual inspection at the protein binding sites and were used for pose-binding selection of the top-ranked compounds.

### 4.4 Antibacterial Assay

MIC and MBC determinations were performed by the broth microdilution technique according to the Clinical and Laboratory Standards Institute guidelines (CLSI, norms M7-A10, 2017) with adaptations. *H. pylori* strain ATCC 43504, amoxicillin-sensitive and metronidazole-resistant, was provided by the Oswaldo Cruz Foundation (Fiocruz, RJ, Brazil). The bacteria were cultured on Columbia Agar supplemented with 5% sheep blood and subsequently inoculated in Brain Heart Infusion (BHI) (Merck Millipore, Germany) supplemented with 10% (v/v) fetal bovine serum, incubated for 72 h at 37 °C (10% CO_2_). In a 96-well micro plate were added 100 μL of *H. pylori* suspension (∼106 cfu/mL; 1:20 McFarland 0.5 solution) in supplemented BHI and 100 μL of chalcone analogues (different concentrations) in the same medium. The micro plate absorbance was recorded at 620 nm and then incubated (37 °C/10% CO_2_/ 72 h). After the incubation period, the micro plate was homogenized, and a new reading was performed to determine the MIC. Amoxicillin was used as standard for antibiotic control.

The MBC was determined by the sample lowest concentration able to inhibit colony formation on Columbia Agar plates (5% sheep blood; 37 °C; 10% CO_2_; 72 h), corresponding to the micro plate well with no apparent growth in BHI.

### 4.5 Cell Lines

Murine fibroblast L-929 (ATCC CCL-1) and human gastric adenocarcinoma cells (AGS) (ATCC CRL-1739) were provided by Rio de Janeiro Cell Bank, Brazil. Cells were maintained in Dulbecco’s Modified Eagle’s Medium (DMEM) supplemented with 10% (v/v) fetal bovine serum and incubated at 37 °C (5% CO_2_ atmosphere).

### 4.6 Cytotoxicity Assay

AGS cells (0.2 mL) were seeded into 96-well tissue-culture plates at a density of 6 × 10^4^ cells/mL and incubated at 37 °C in a 5% CO_2_ atmosphere for 24 h. After incubation, the medium was removed and replaced with fresh medium containing different concentrations of the tested compounds or an unmodified medium (control). The plates were incubated for an additional 48 h under the same conditions.

Stock solutions (20 mg/mL) were prepared in DMSO and diluted in a culture medium to obtain the desired concentrations (up to 25 μg/mL). The final DMSO concentration did not exceed 0.125% (v/v). Vehicle control experiments were performed at the same DMSO concentration, and no significant effect on the cell viability was observed.

All working solutions were visually inspected before and after incubation. No visible precipitation or solid deposition at the bottom of the wells was observed during the cytotoxicity assays.

Subsequently, the medium was removed, and the plates were processed according to the MTT–tetrazolium method.^65^ After brief agitation, the optical density of each well was measured at 540 nm with a reference wavelength of 620 nm. Dose–response curves and the concentration required to reduce cell viability by 50% (IC_50_) were calculated by linear regression analysis with a 95% confidence interval. Cisplatin was used as a reference cytotoxic agent.

### 4.7 Statistical Analysis

Linear regression analysis with 95% confidence limit was used to define the dose–response curve and to compute the cytotoxic index that is needed to reduce the absorbance of the system by 50% (IC_50_), the so-called cytotoxic midpoint, in both cytotoxic assays. All results were obtained from three independent experiments and expressed as mean ± SD (*n* = 3). Statistically significant differences were determined by two-way analysis of variance with the posthoc Bonferroni test (using the GraphPad Prism 5.0 statistics package). Means were statistically significant at *p* < 0.05.

## Supplementary Material



## Notes

Infrared (IR), UV–vis, ^1^H, ^13^C NMR, and mass spectrometry spectra of the ligands and complexes, which confirm their molecular structures; thermogravimetric analysis (TG) data, providing additional evidence for the purity and stability of the compounds; and details and images from the molecular docking study, which illustrate the interactions of the complexes with the investigated biological targets (PDF)
