# Supplementary material for: Synthesis, Characterization, and Evaluation of Palladium(II) Complexes Containing Chalcones on Gastric Adenocarcinoma Cells and Helicobacter pylori
Source: ACS Omega. 2026 Apr 27;11(18):26159–77. doi: 10.1021/acsomega.5c07262 (PMC13177237; doi:10.1021/acsomega.5c07262)
Supplement: Supplementary file 1 [file ao5c07262_si_001.pdf]

Synthesis, characterization and evaluation of palladium(II) complexes containing chalcones on gastric adenocarcinoma cells and *Helicobacter pylori*

Jéssica Rodrigues Pereira de Oliveira Borlot[a], Rodrigo de Almeida Romagna[b], Ricardo Machado Kuster[a,c], Marcos Antônio Ribeiro[a,c], Priscilla Paiva Luz[a,c], Rita de Cássia Ribeiro Gonçalves[b], Reginaldo Bezerra dos Santos[c], Rodrigo Rezende Kitagawa[a,b]*

[a]Graduate Program of Chemistry, Exact Sciences Center, Federal University of Espirito Santo, Avenida Fernando Ferrari 514, Goiabeiras, 29075-910, Vitoria, ES, Brazil

[b]Graduate Program of Pharmaceutical Sciences, Health Sciences Center, Federal University of Espirito Santo, Avenida Marechal Campos 1468, Bonfim, 29047-105, Vitoria, ES, Brazil

[c]Department of Chemistry, Exact Sciences Center, Federal University of Espirito Santo, Avenida Fernando Ferrari 514, Goiabeiras, 29075-910, Vitoria, ES, Brazil.

* Corresponding author

1 Supporting Information

1.1 Infrared Characterization

Figure S1. Infrared spectrum of ligand **1**

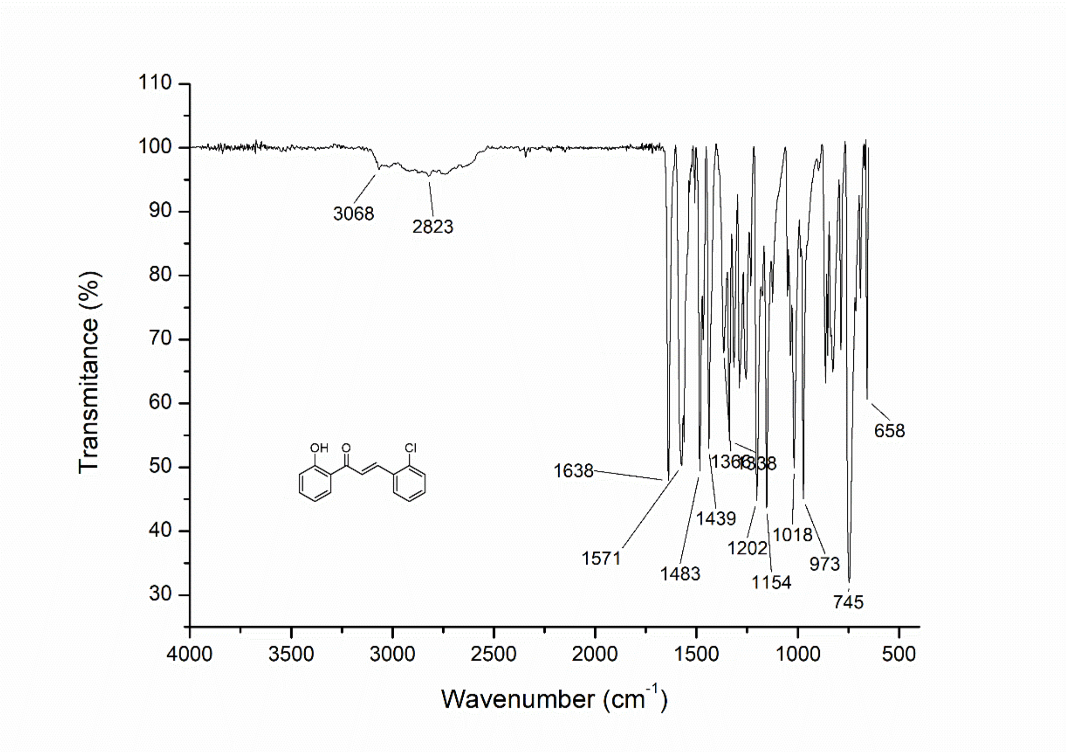

Figure S2. Infrared spectrum of complex **1C**

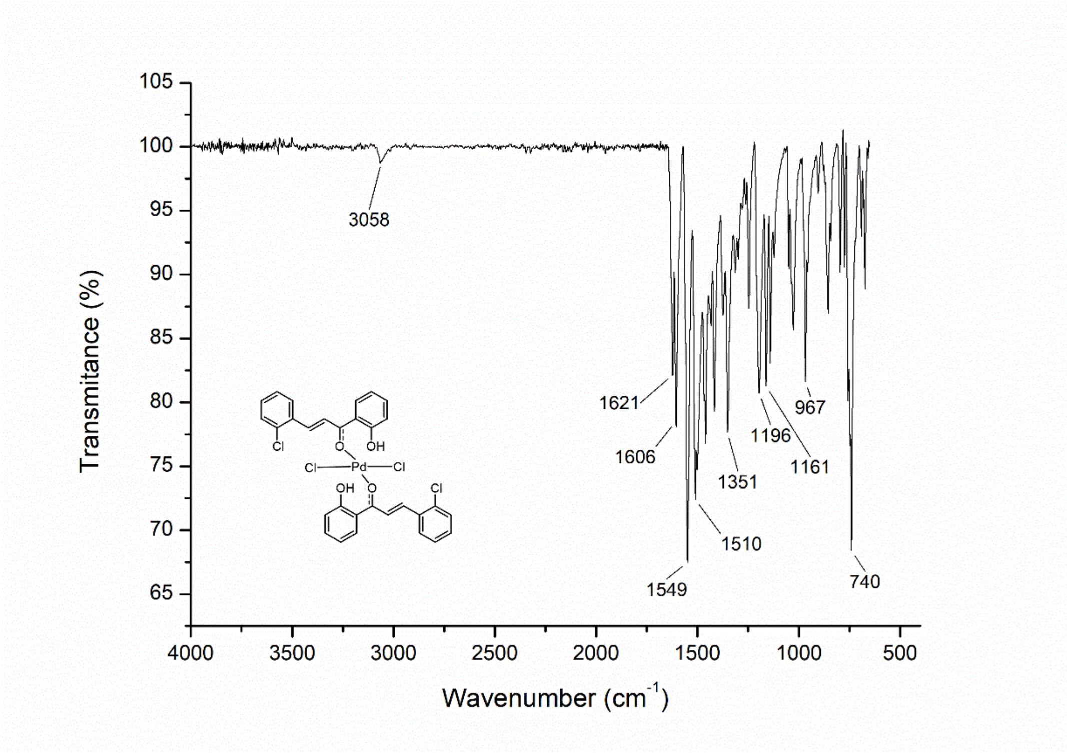

Figure S3. Infrared spectrum of ligand **2**

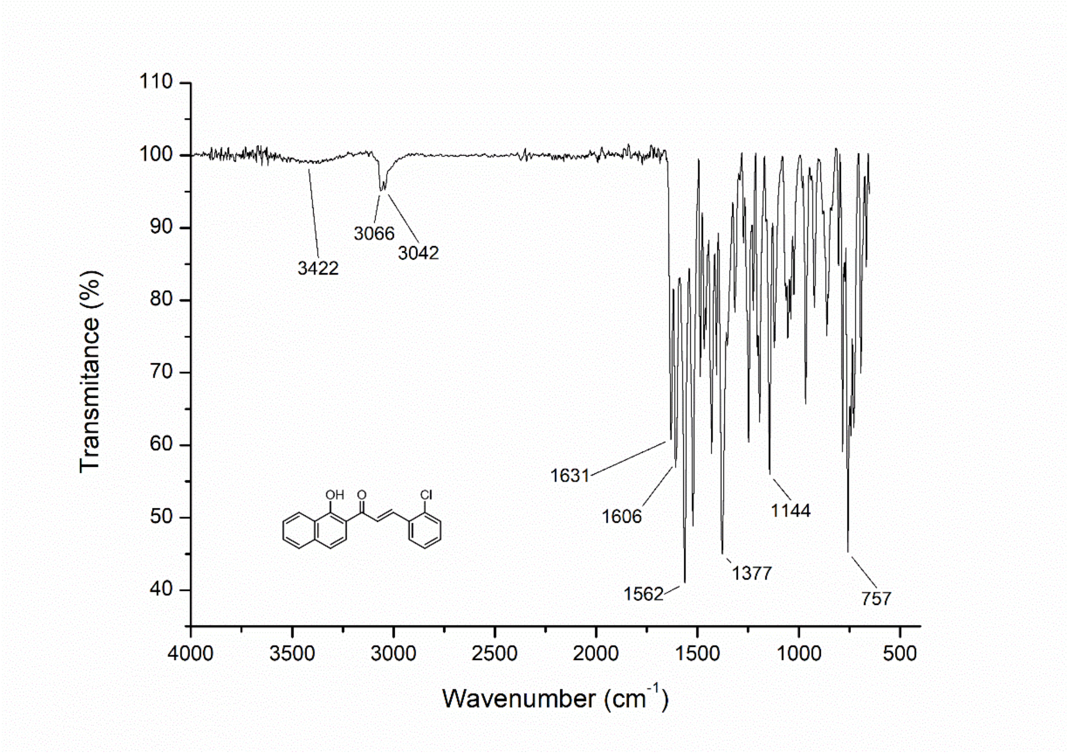

Figure S4. Infrared spectrum of complex **2C**

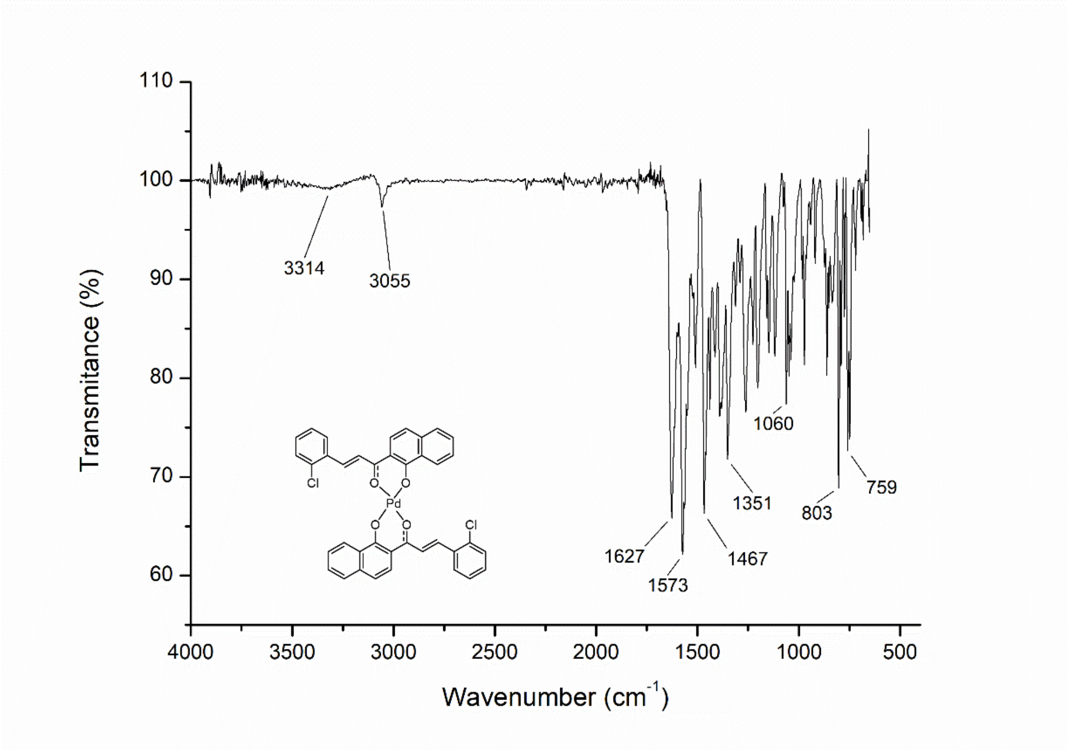

Figure S5. Infrared spectrum of ligand **3**

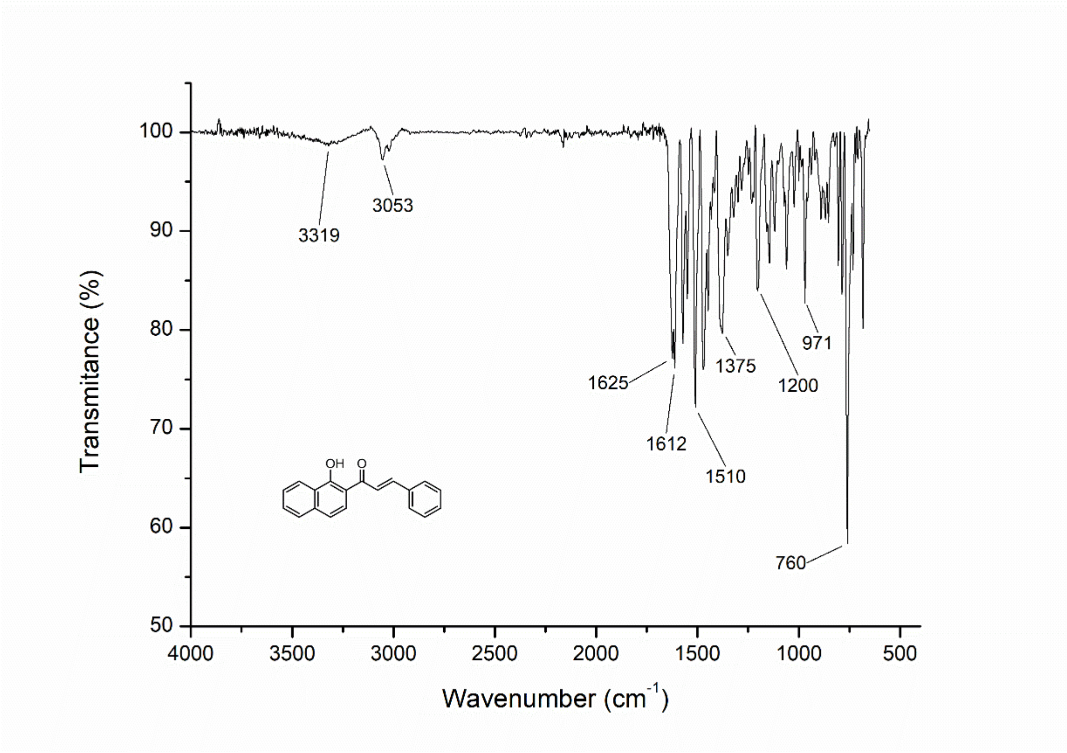

Figure S6. Infrared spectrum of complex **3C**

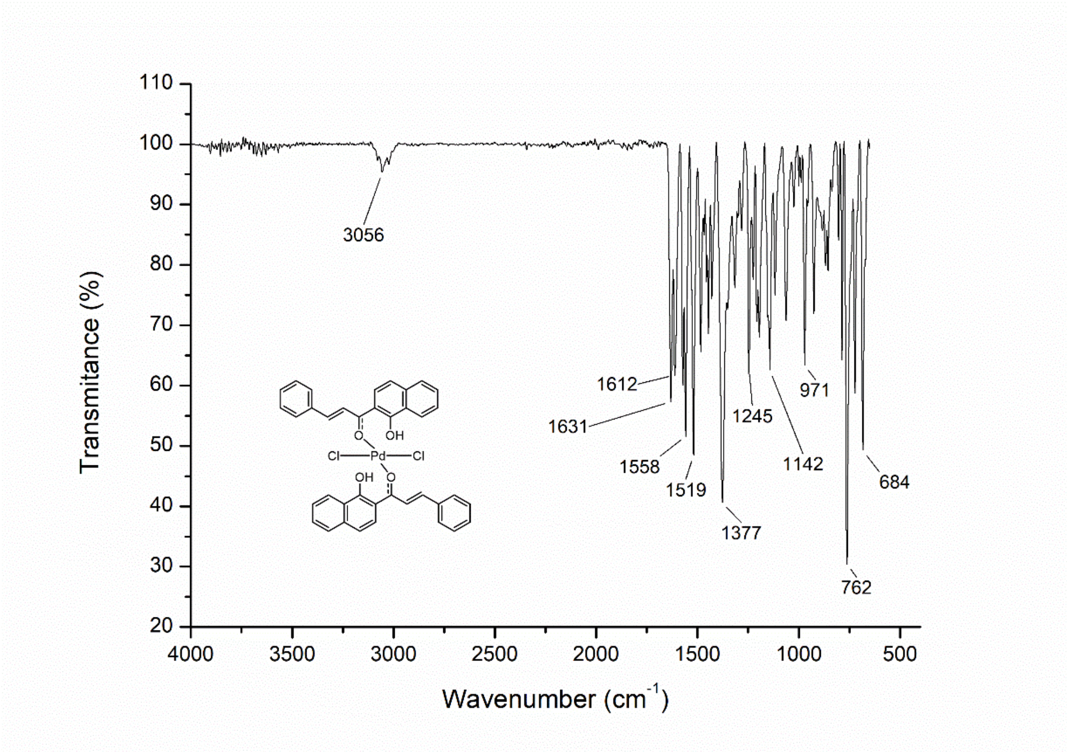

Figure S7. Infrared spectrum of ligand **4**

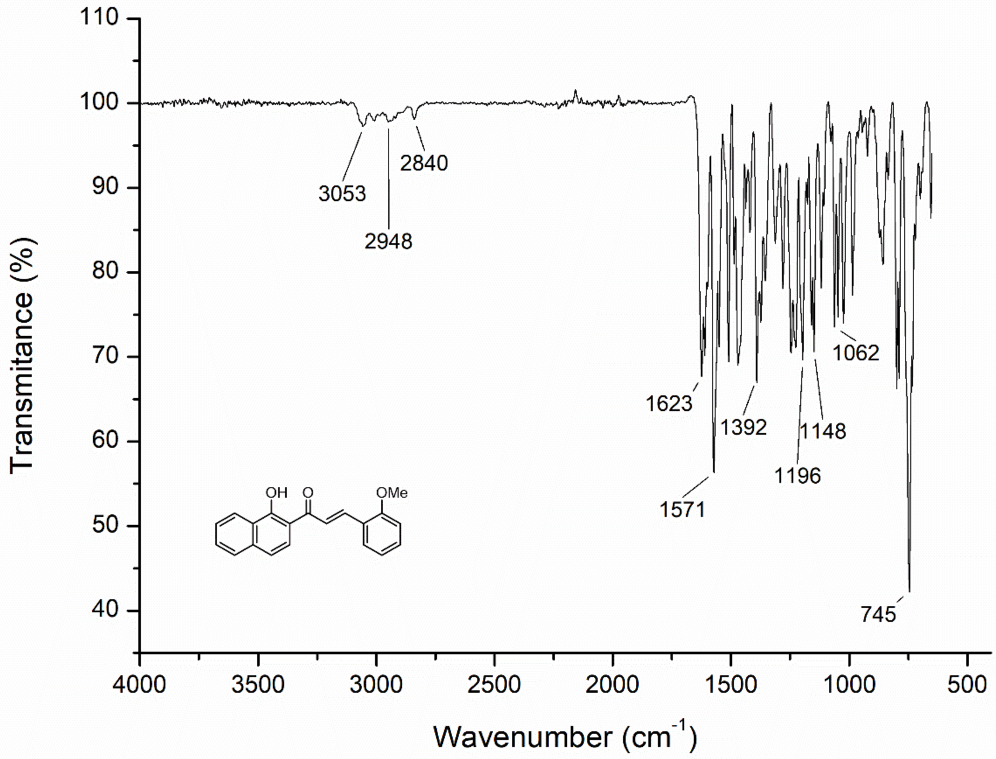
110
100
90
80
70
60
50
40
Transmitance (%)
4000
3500
3000
2500
2000
1500
1000
500
Wavenumber (cm-1)
3053
2948
2840
1623
1571
1392
1196
1148
1062
745
OH
O
OMe

Figure S8. Infrared spectrum of complex **4C**

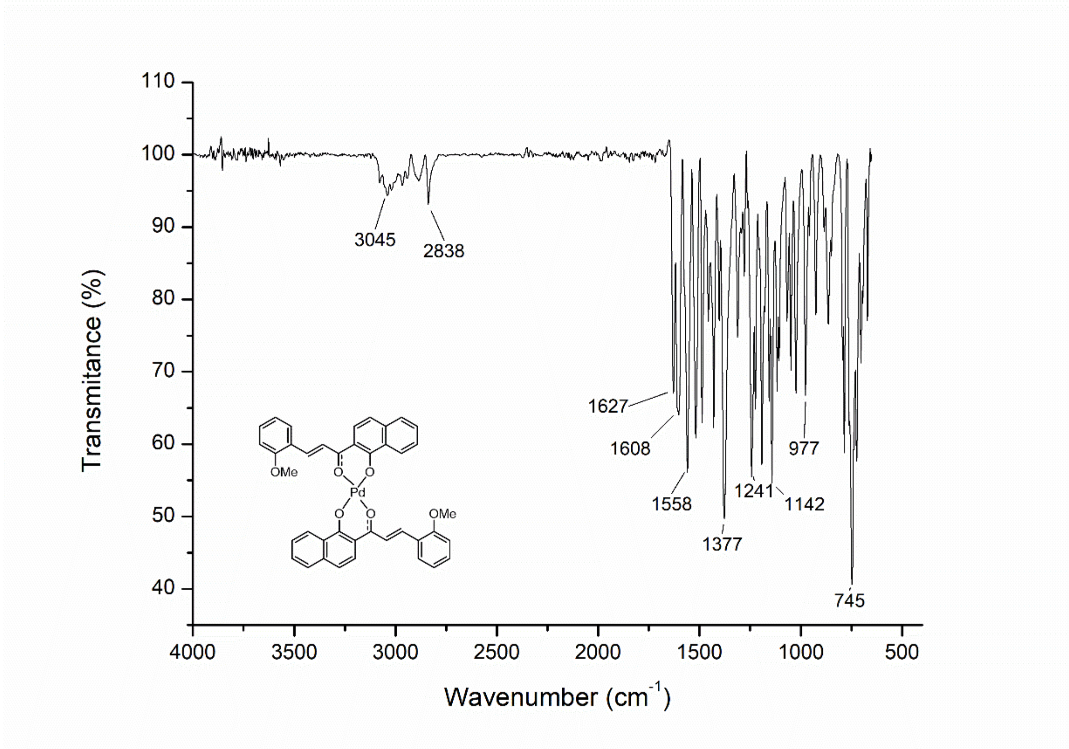

Figure S9. Infrared spectrum of ligand **5**

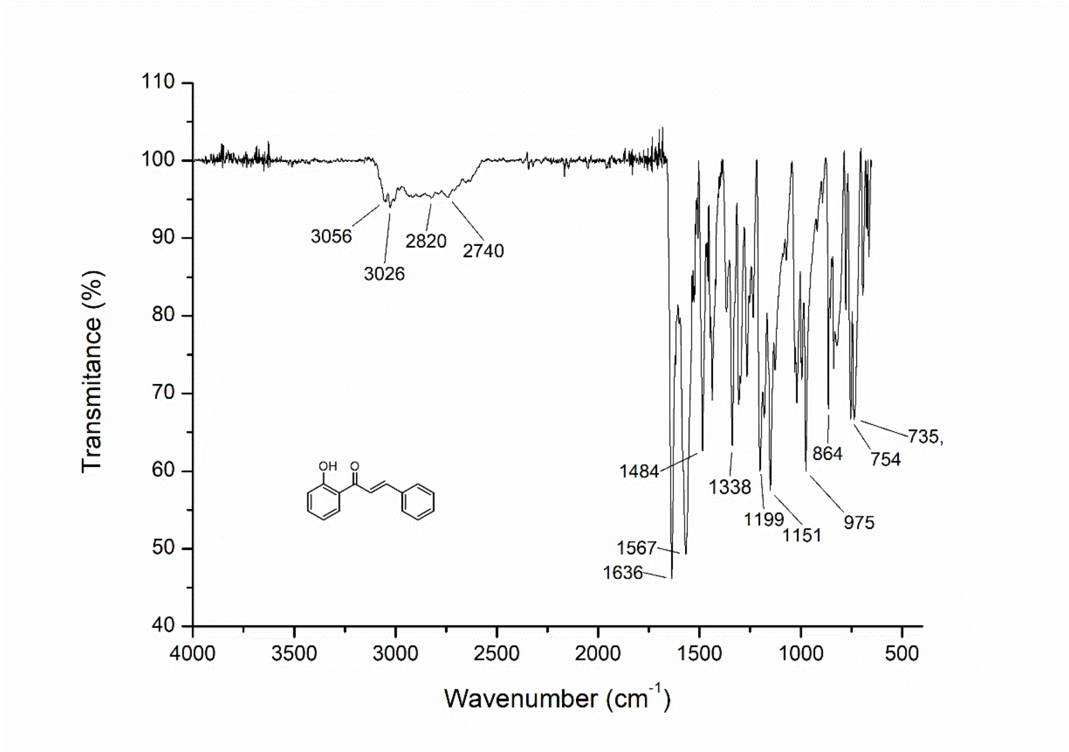

Figure S10. Infrared spectrum of complex **5C**

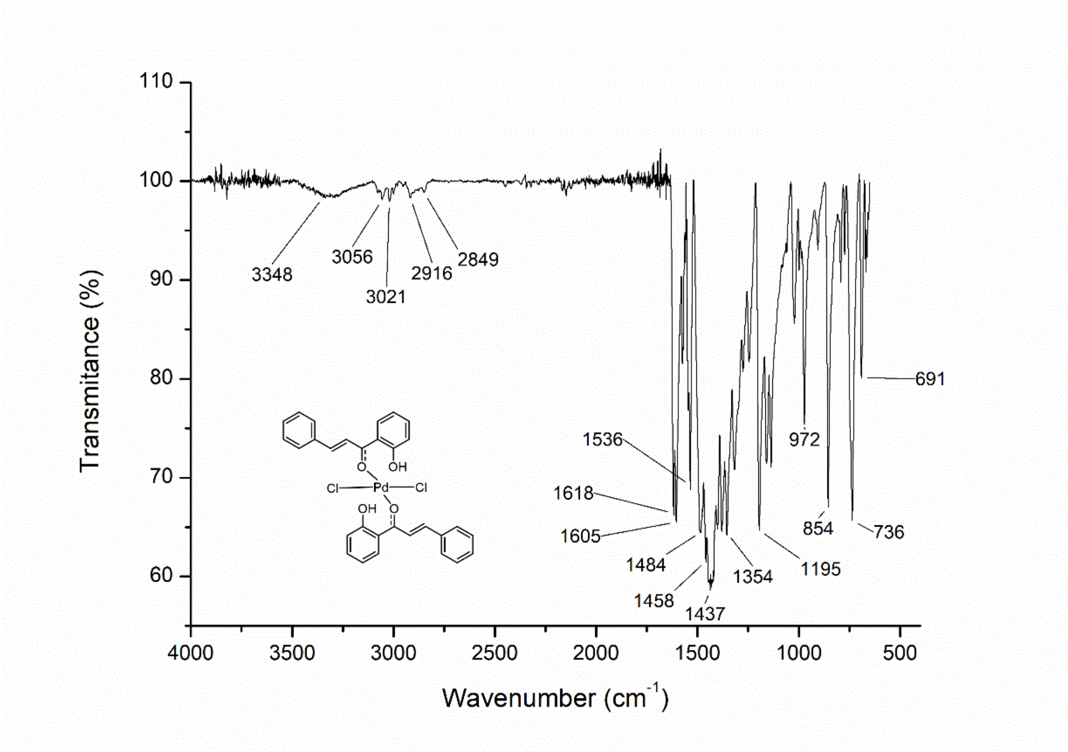

Figure S11. Infrared spectrum of ligand **6**

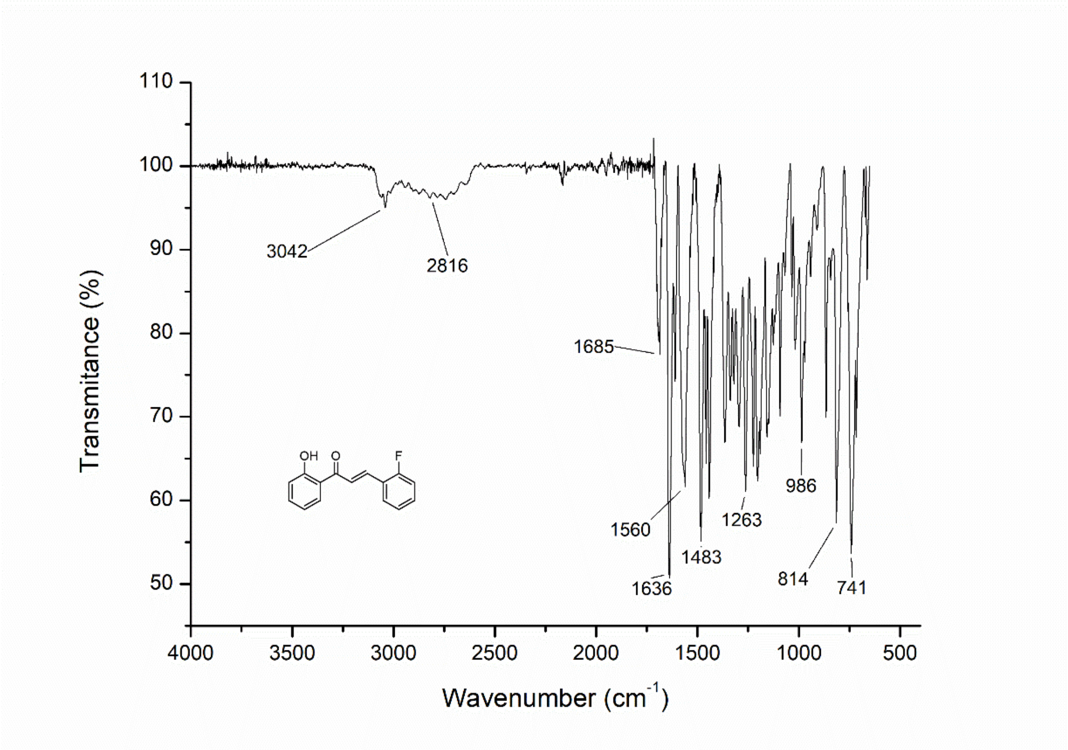

Figure S12. Infrared spectrum of complex 6C

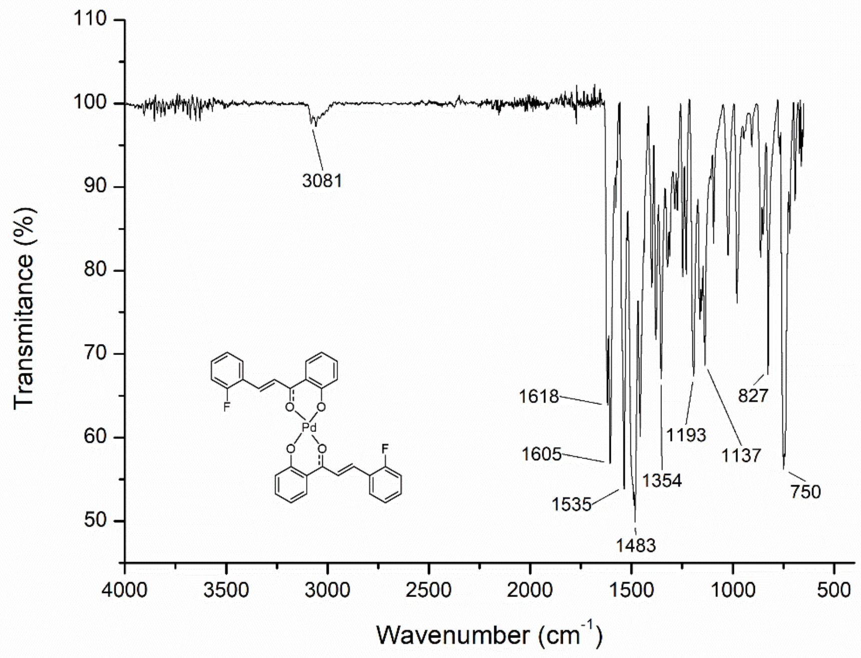

Figure S13. Infrared spectrum of ligand 7

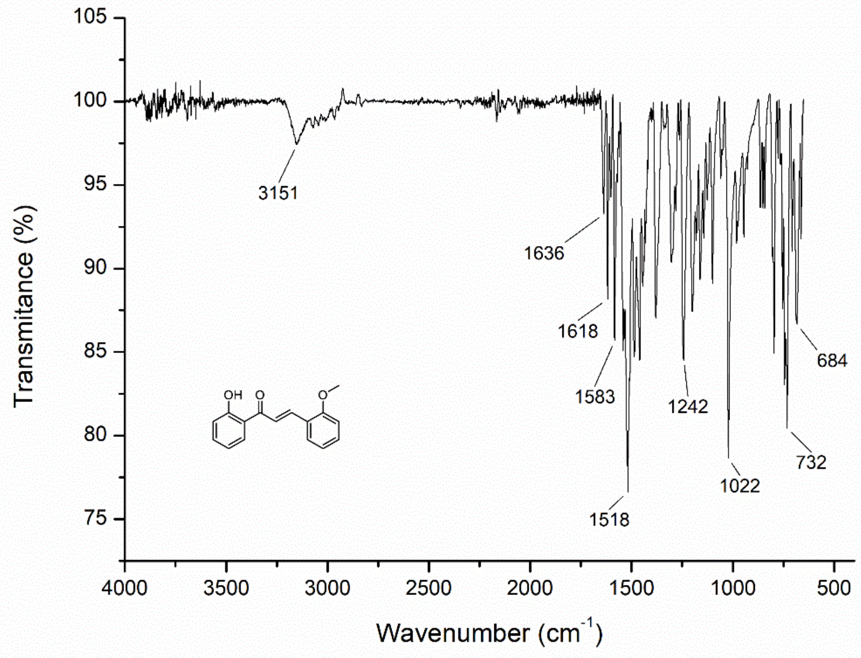

Figure S14. Infrared spectrum of complex **7C**

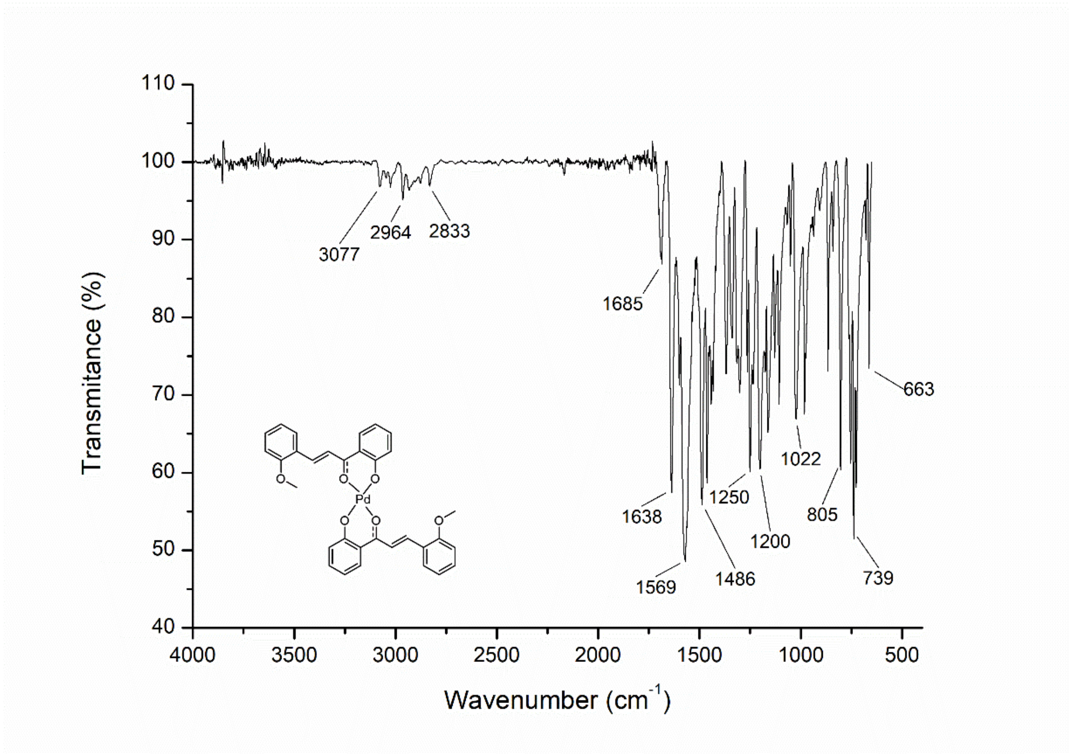

Figure S15. Infrared spectrum of ligand **8**

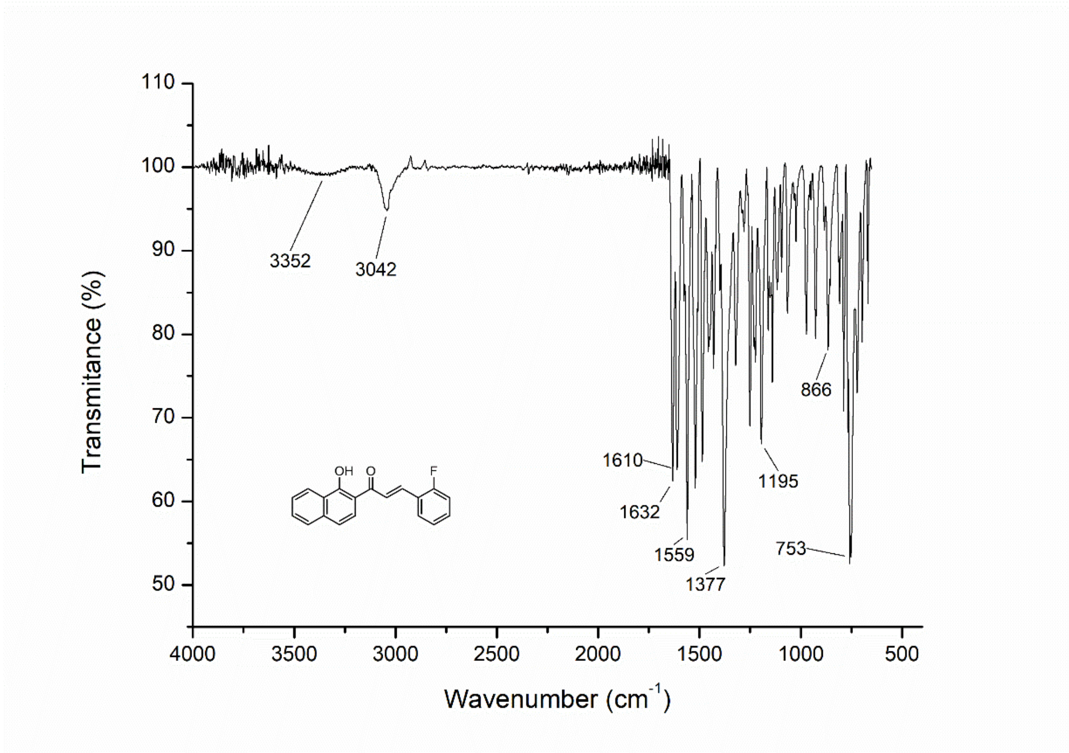

Figure S16. Infrared spectrum of complex **8C**

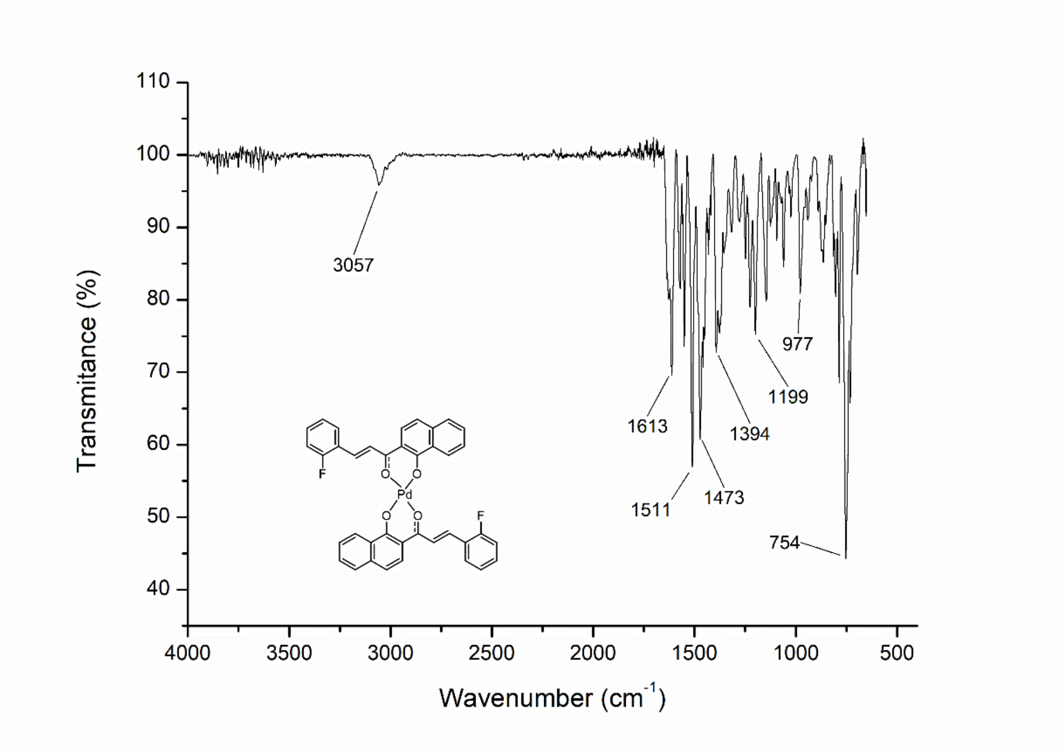

1.2 UV-vis Characterization

Figure S17. UV-vis spectrum of complex **1C** and its ligand

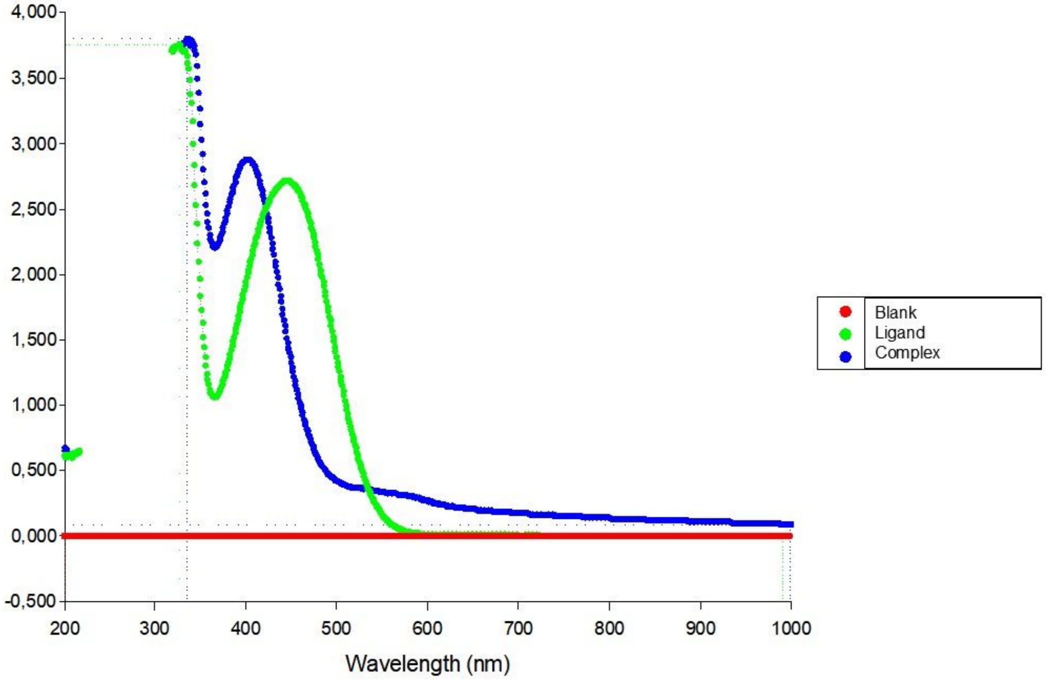

Figure S18. UV-vis spectrum of complex **2C** and its ligand

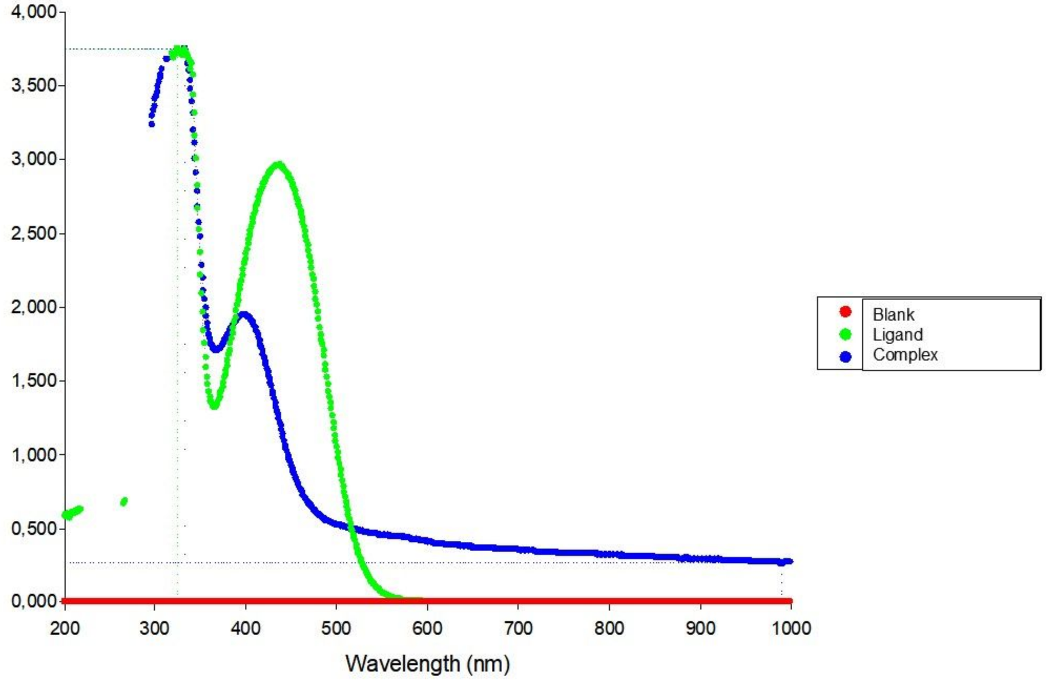

Figure S19. UV-vis spectrum of complex **3C** and its ligand

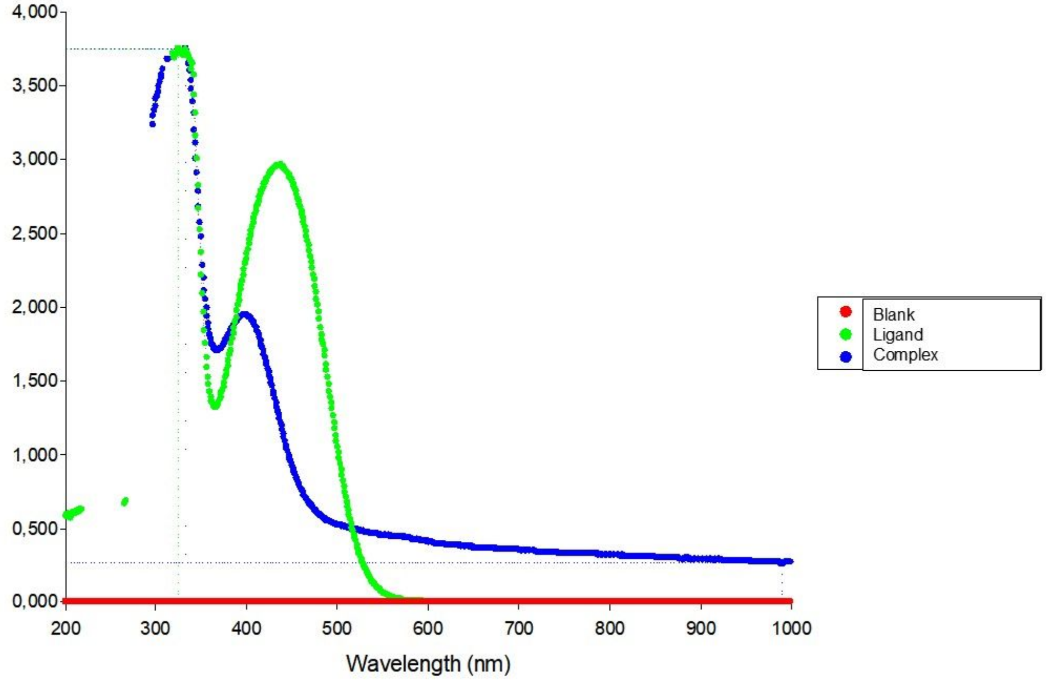

Figure S20. UV-vis spectrum of complex **4C** and its ligand

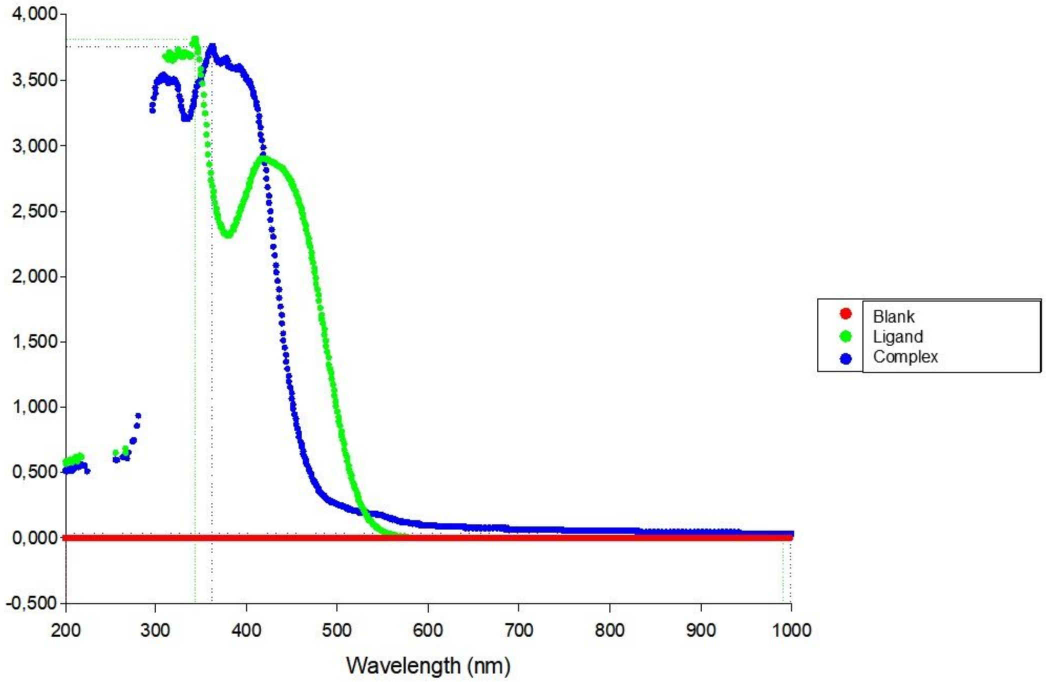

Figure S21. UV-vis spectrum of complex **5C** and its ligand

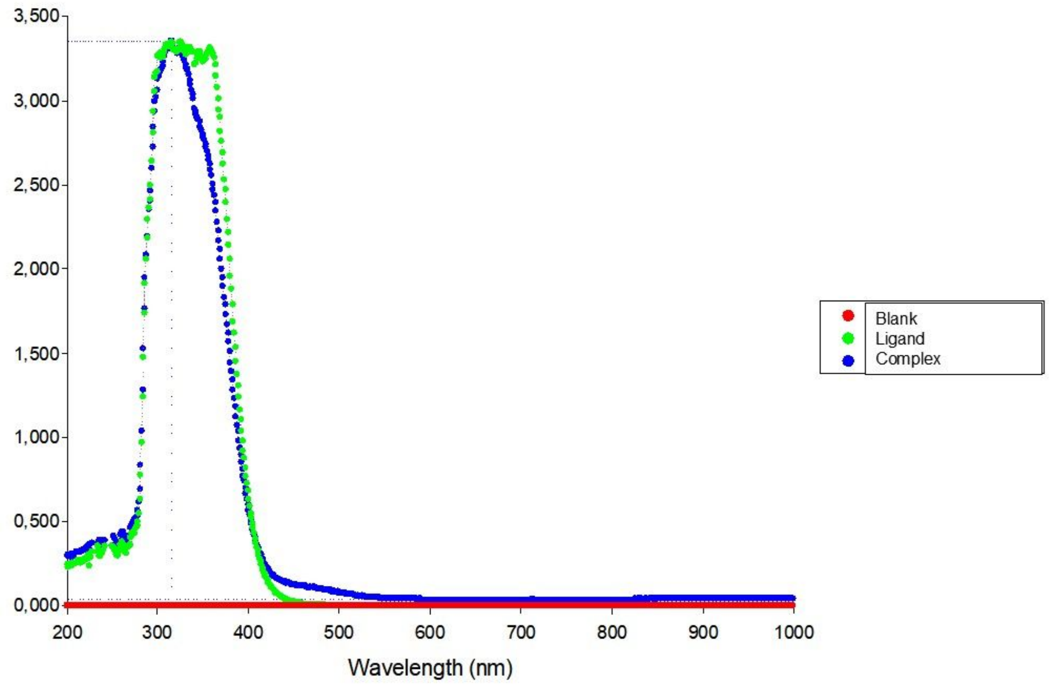

Figure S22. UV-vis spectrum of complex **6C** and its ligand

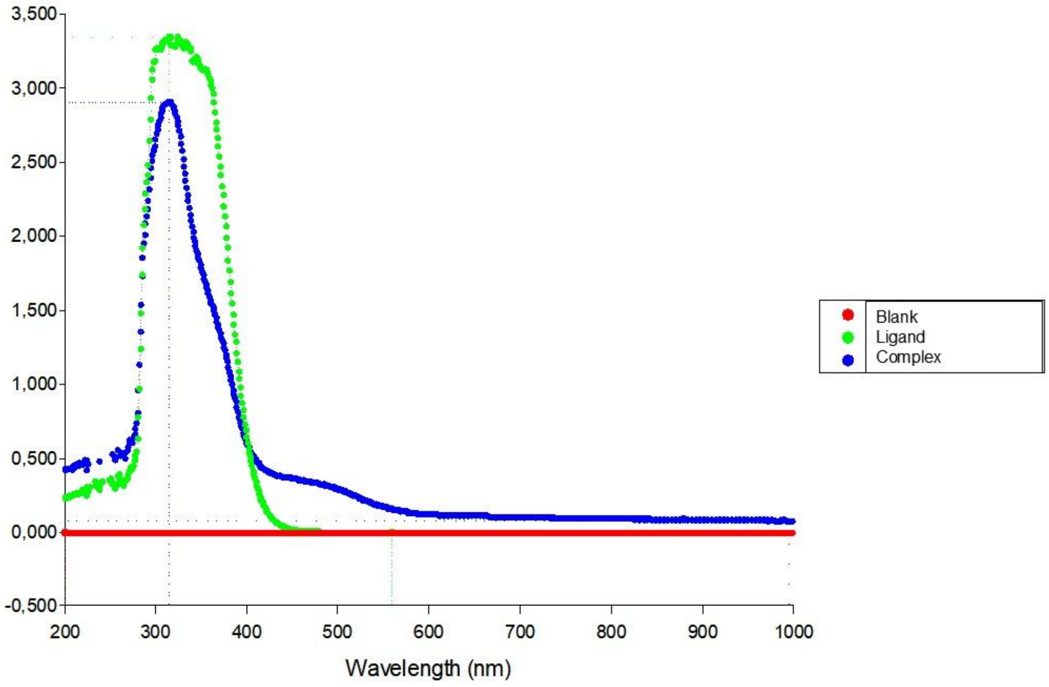

Figure S23. UV-vis spectrum of complex **7C** and its ligand

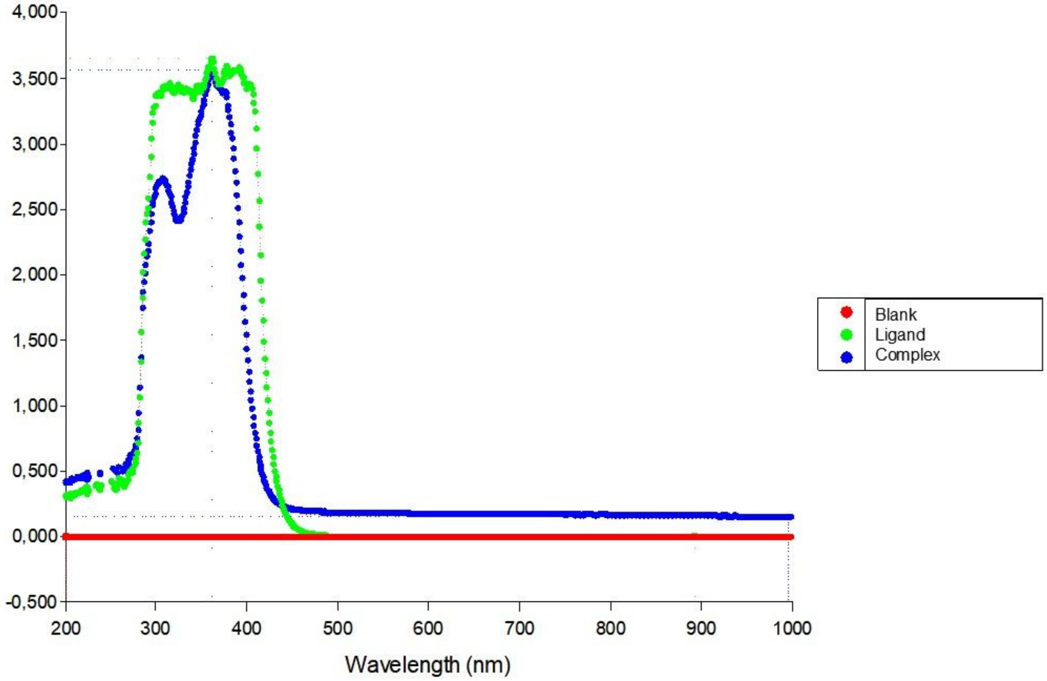

Figure S24. UV-vis spectrum of complex 8C and its ligand

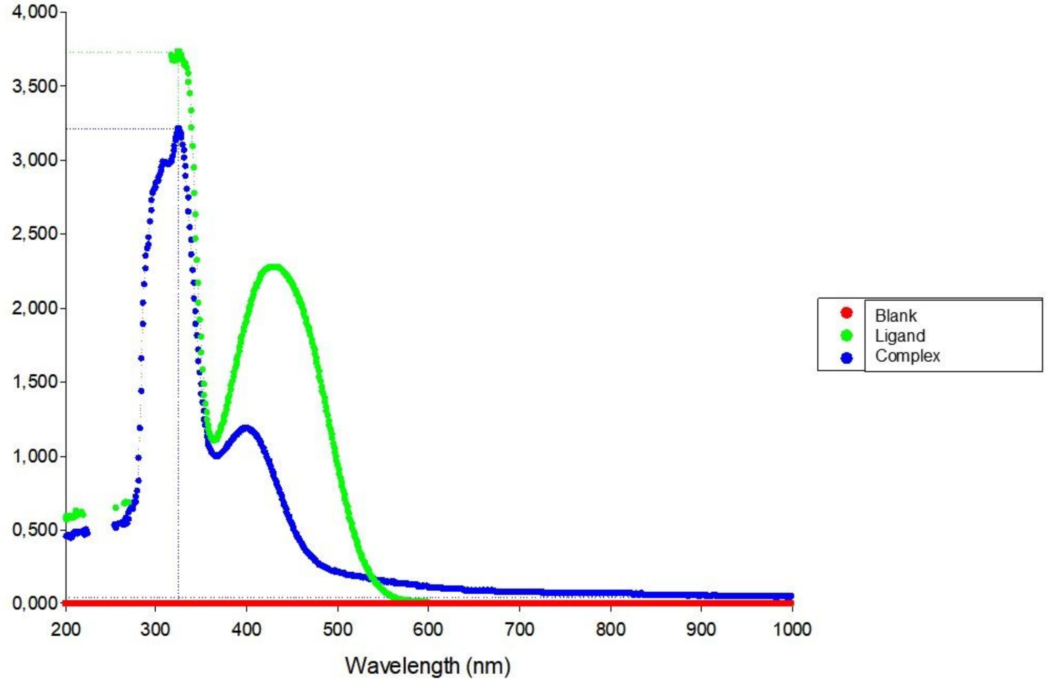

1.3 RMN Characterization by NMR spectroscopy

Figure S25. ^{1}H NMR spectra of ligand **1** and complex **1C** recorded in DMSO-d$_6$ (400 MHz).

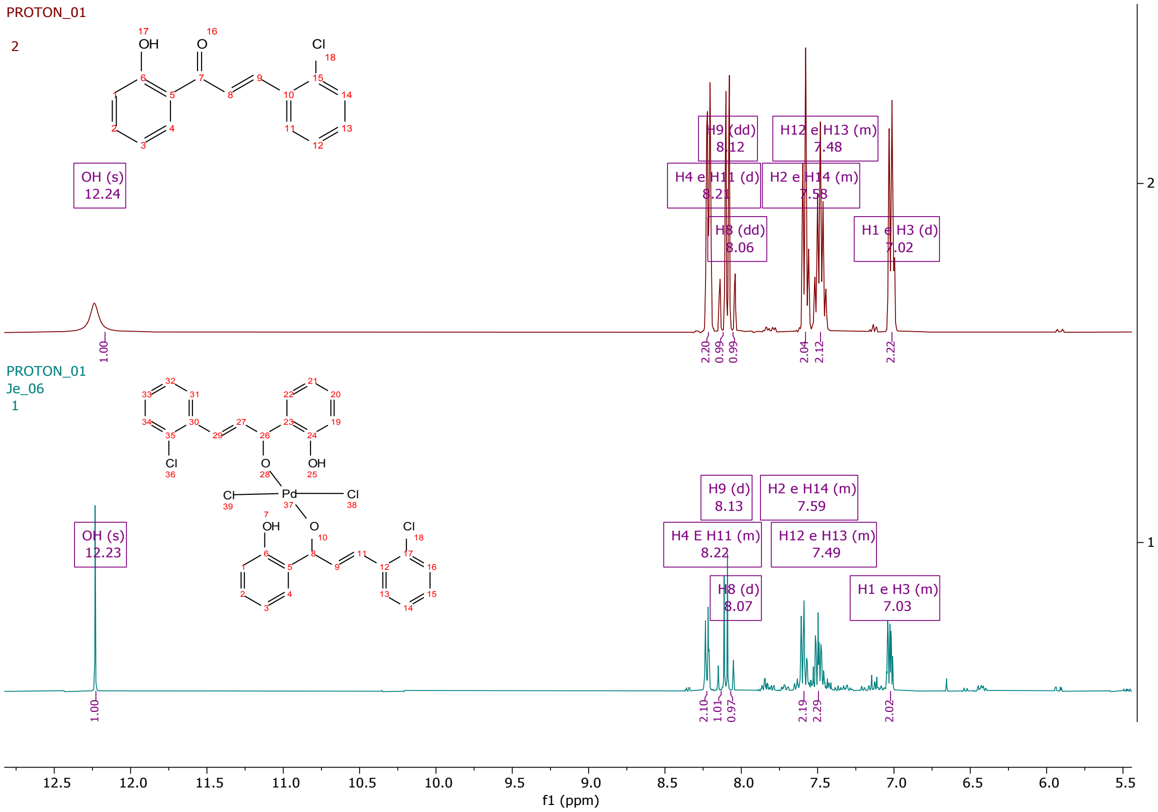

Figure S26. ^{1}H NMR spectra of ligand **2** and complex **2C** recorded in DMSO-d_6 (400 MHz).

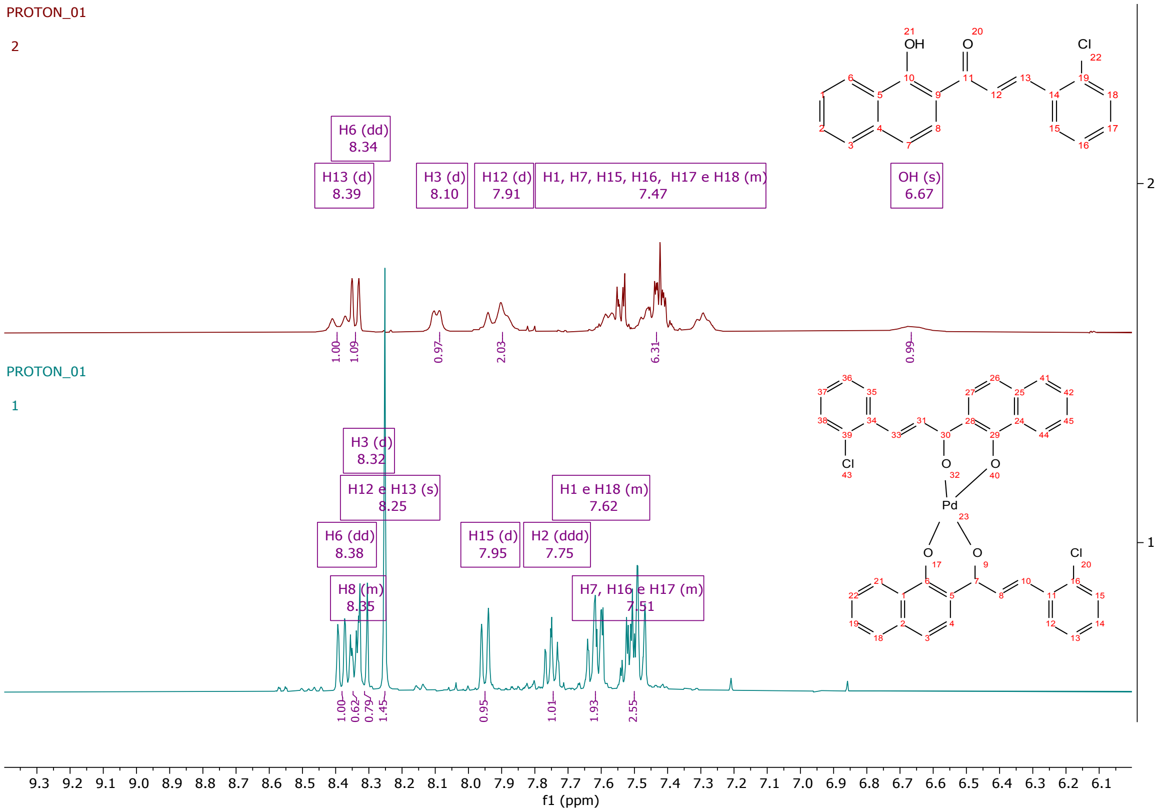

Figure S27. ^{1}H NMR spectra of ligand **3** and complex **3C** recorded in $CDCl_3$ (400 MHz).

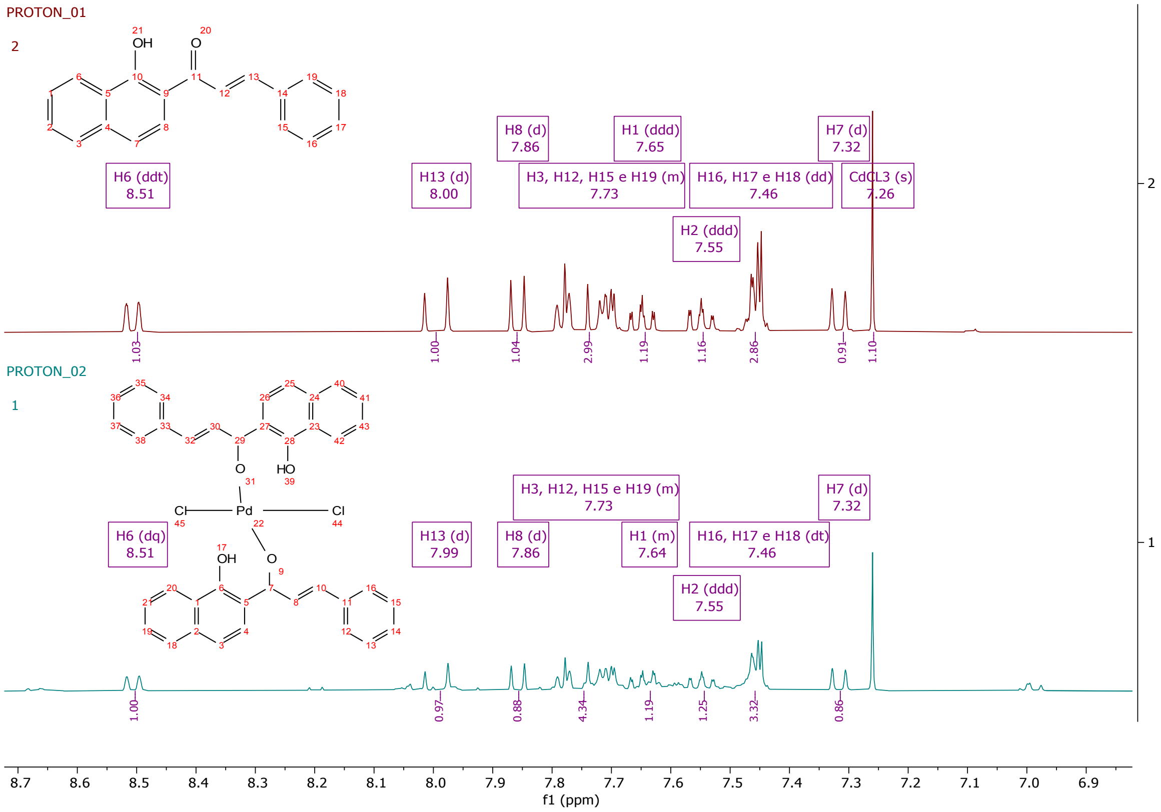

Figure S28. ^{1}H NMR spectra of ligand **4** and complex **4C** recorded in DMSO-d_6 (400 MHz).

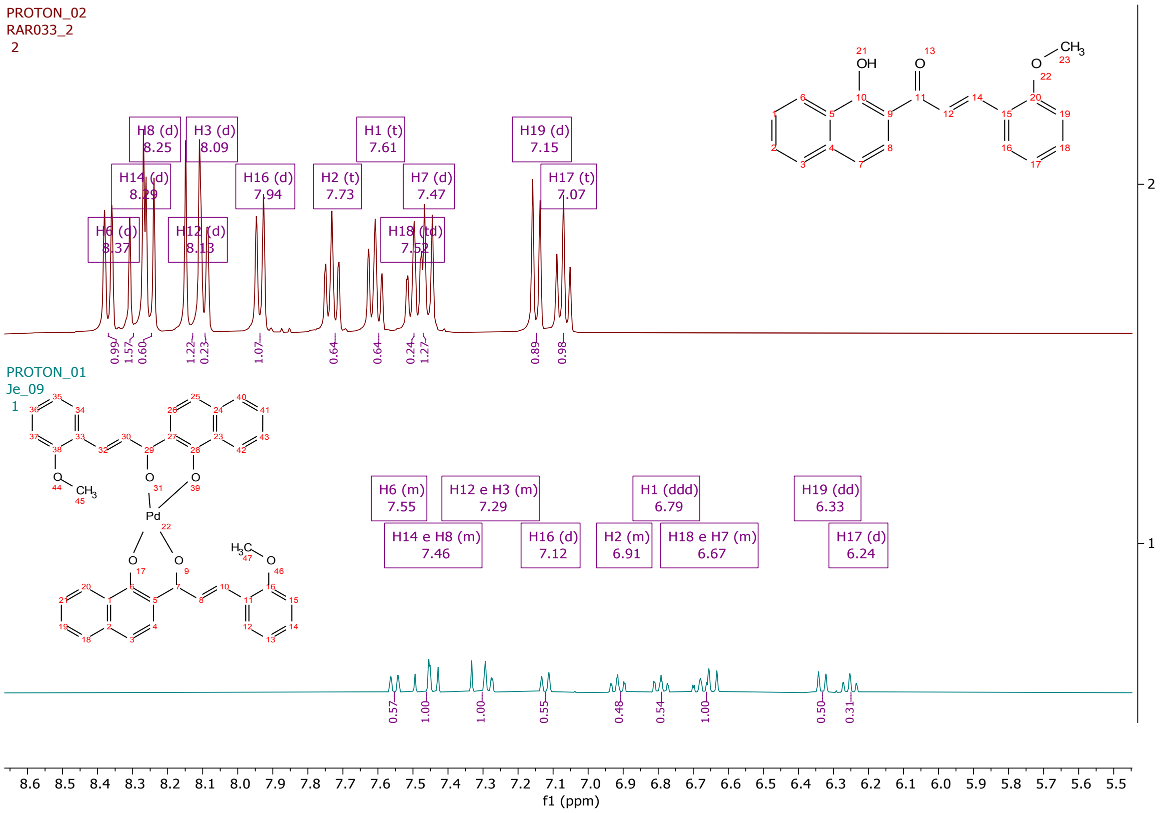

Figure S29. ^{1}H NMR spectra of ligand **5** and complex **5C** recorded in DMSO-d_6 (400 MHz).

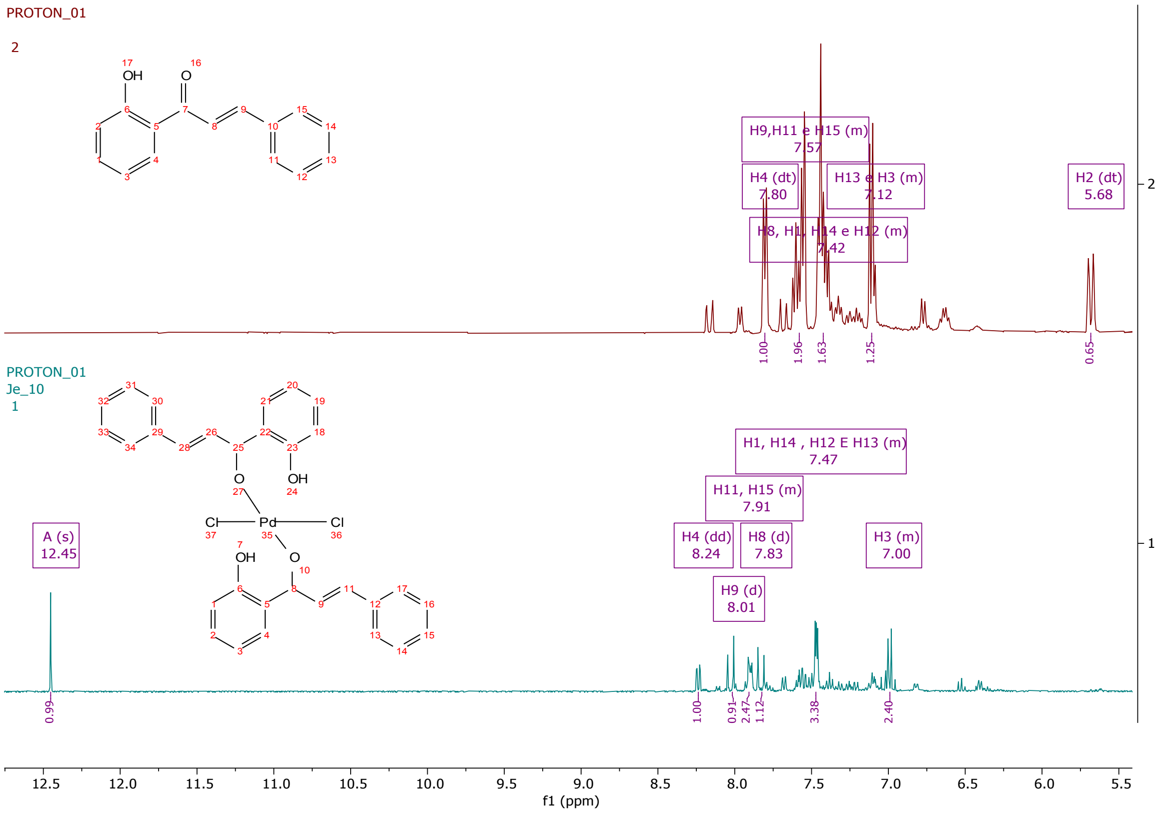

Figure S30. ^{1}H NMR spectra of ligand **6** and complex **6C** recorded in DMSO-d_6 (400 MHz).

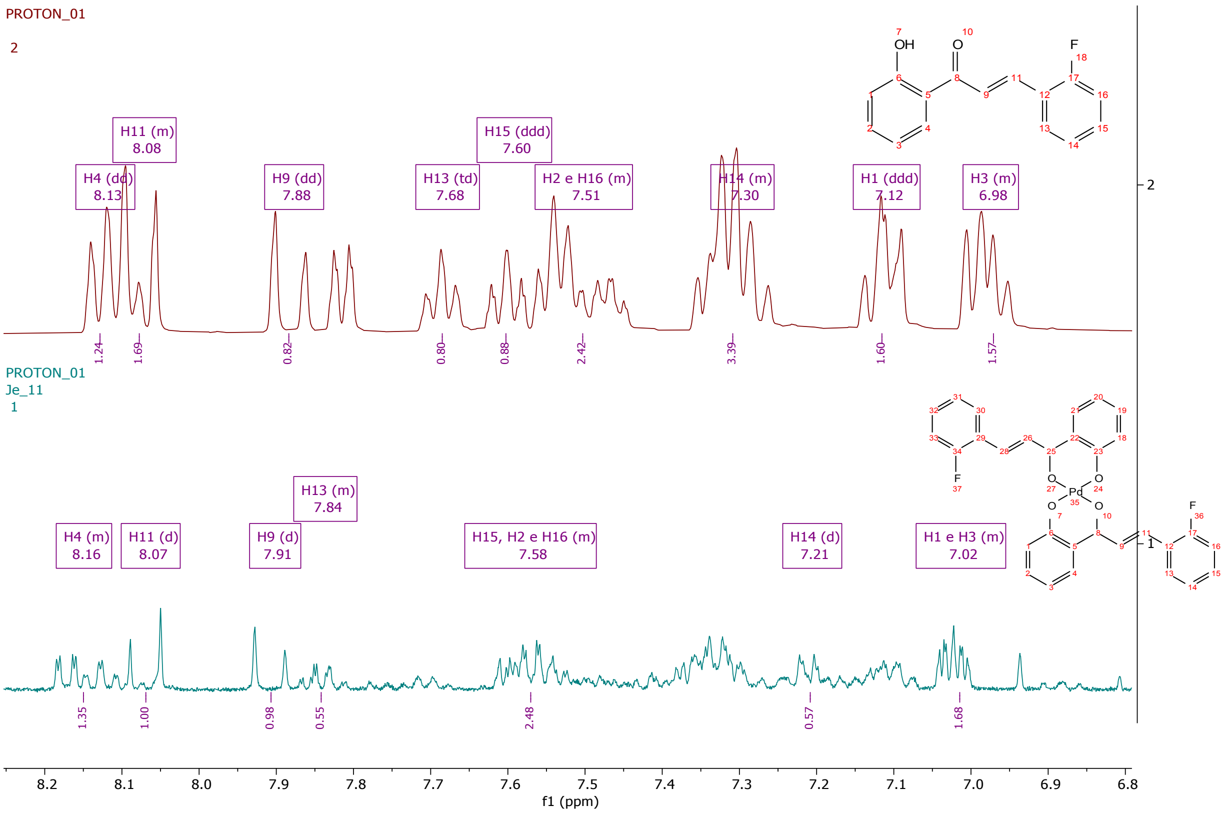

Figure S31. ^{1}H NMR spectra of ligand **7** and complex **7C** recorded in DMSO-d_6 (400 MHz).

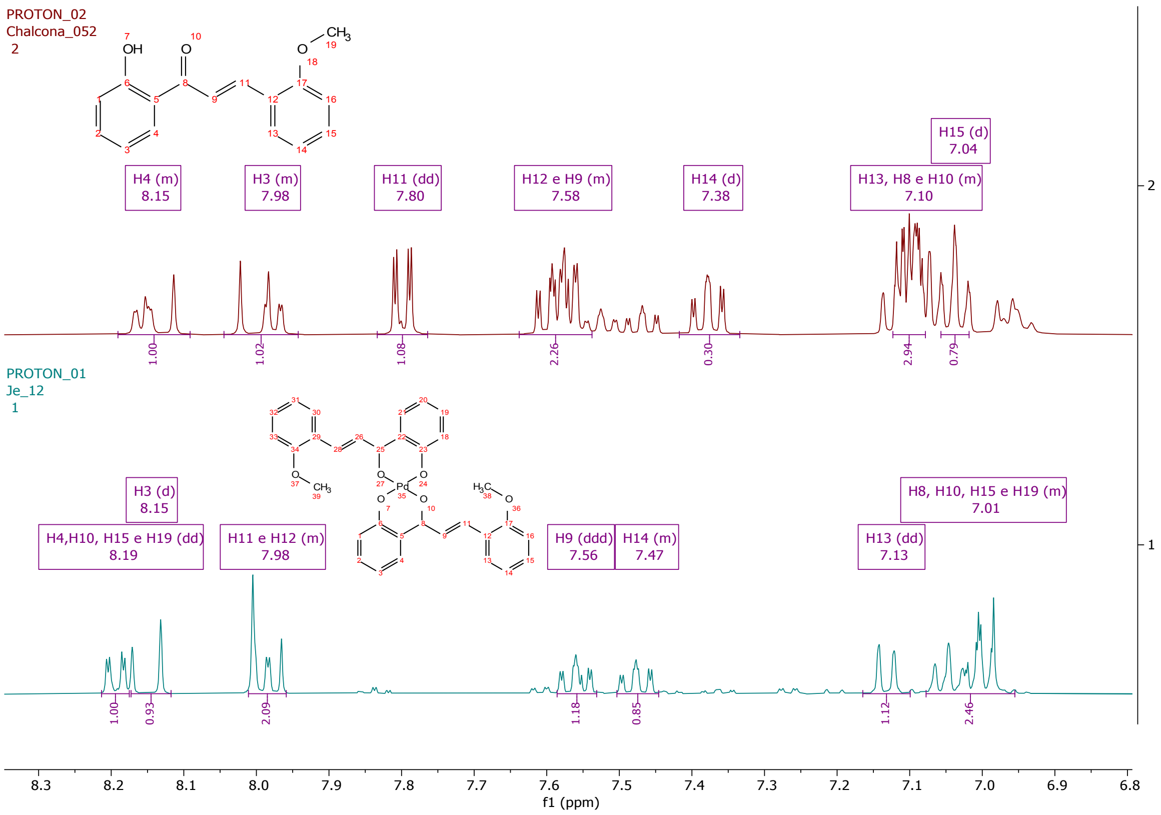

Figure S32. ^{1}H NMR spectra of ligand **8** and complex **8C** recorded in DMSO-d_6 (400 MHz).

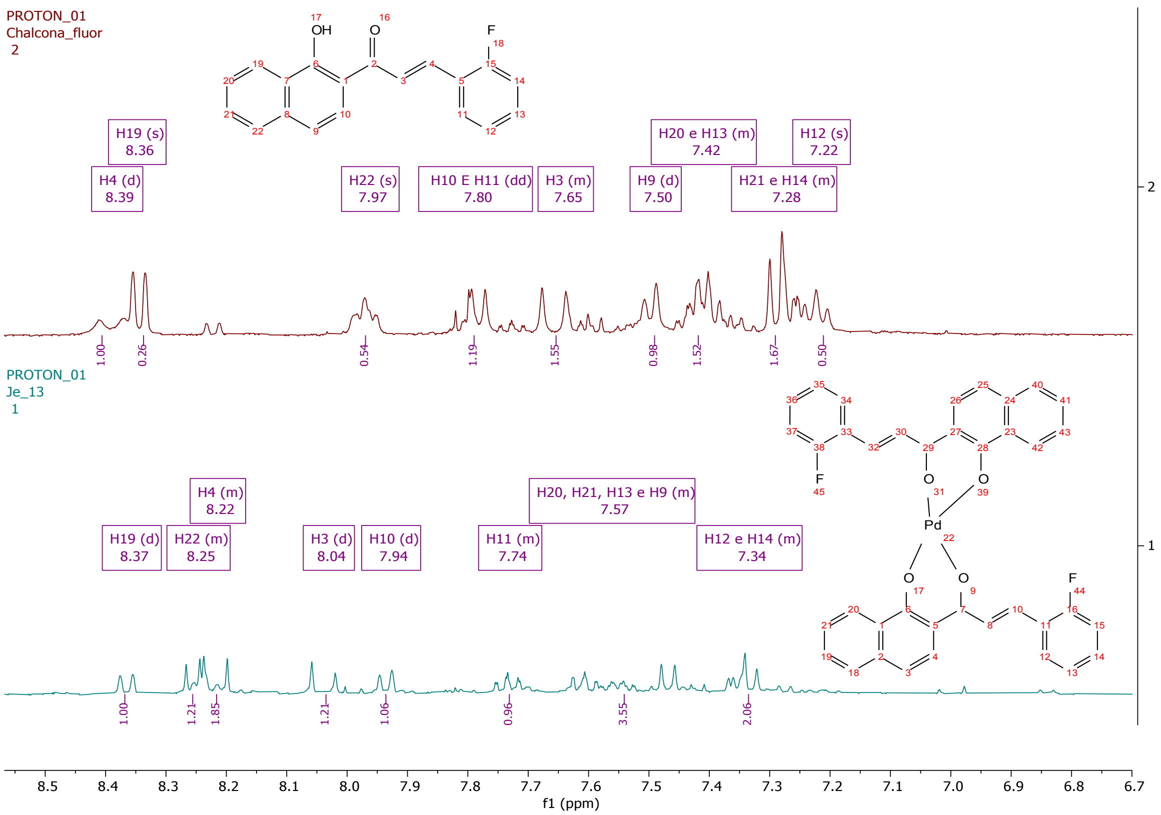

1.4 Thermogravimetric Analysis

Figure S33. TG Curve of Complex **1C**

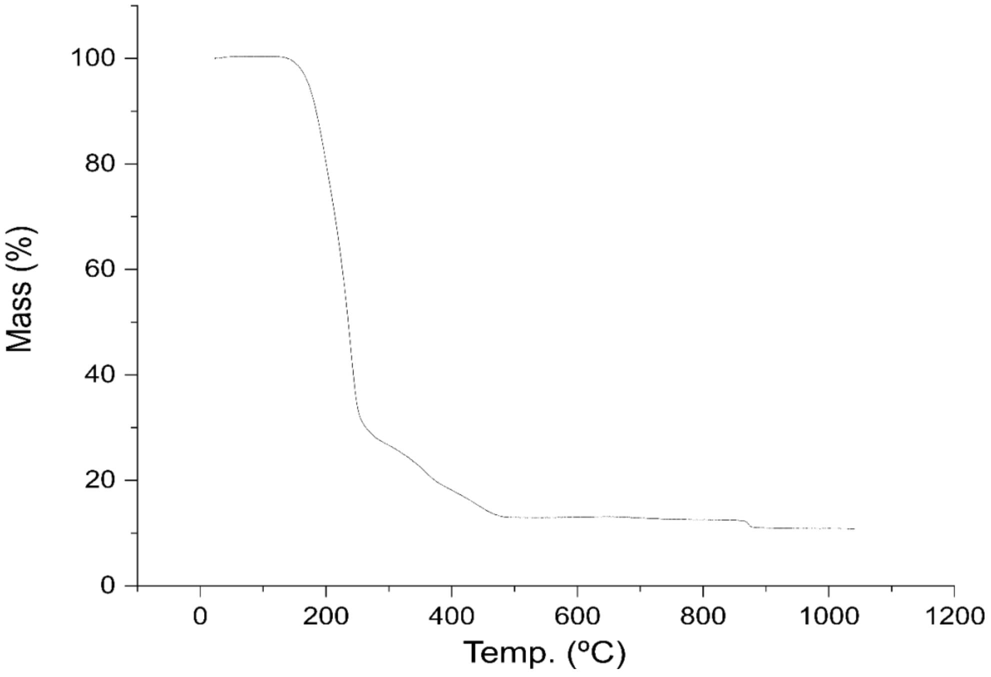

Figure S34. TG Curve of Complex **2C**

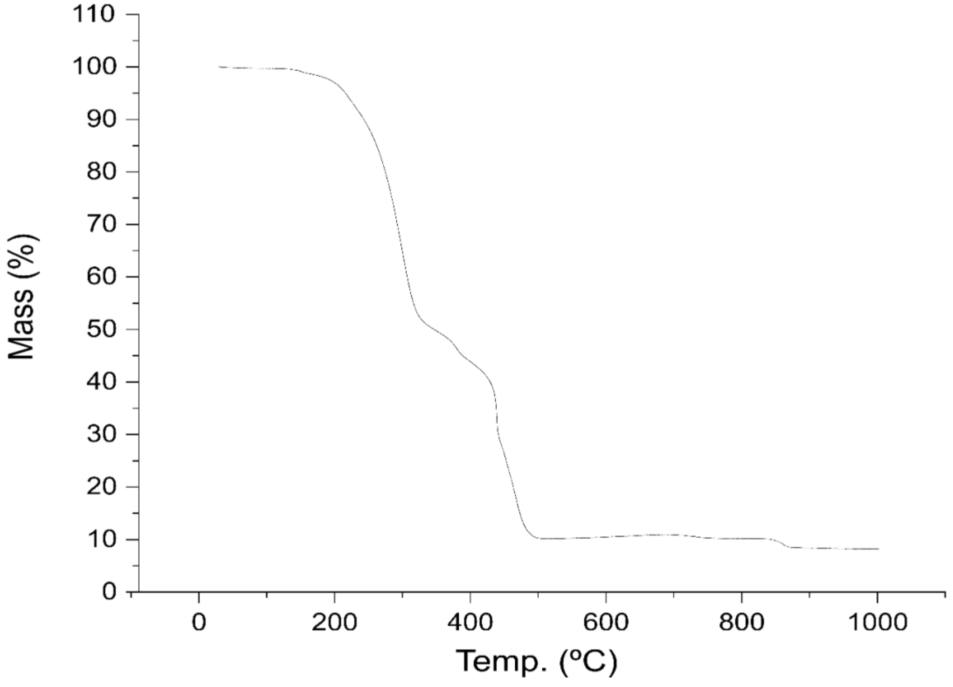

Figure S35. TG Curve of Complex **3C**

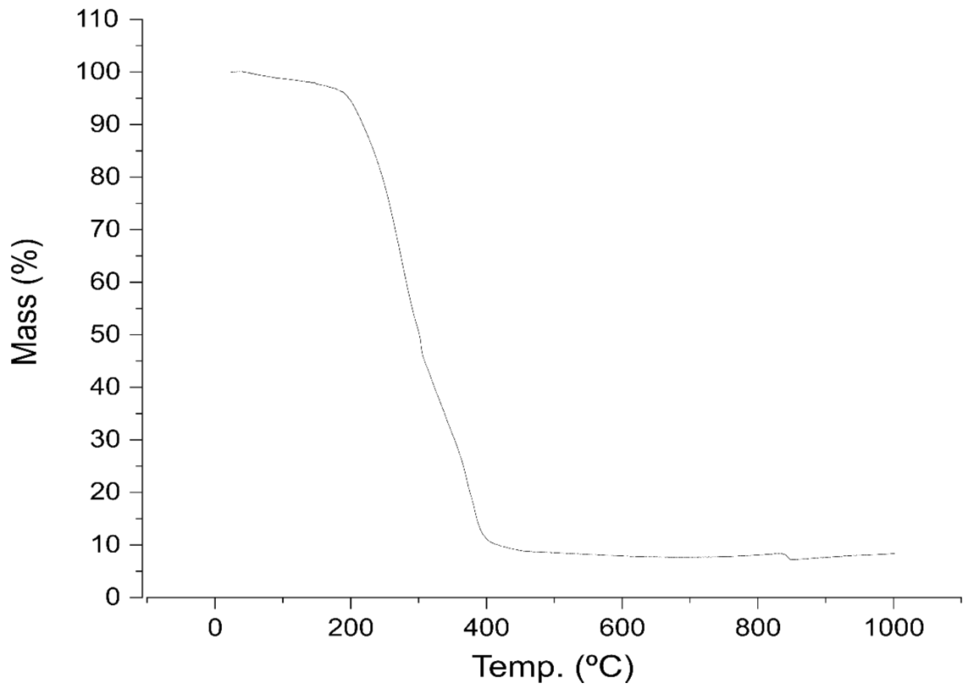

Figure S36. TG Curve of Complex **4C**

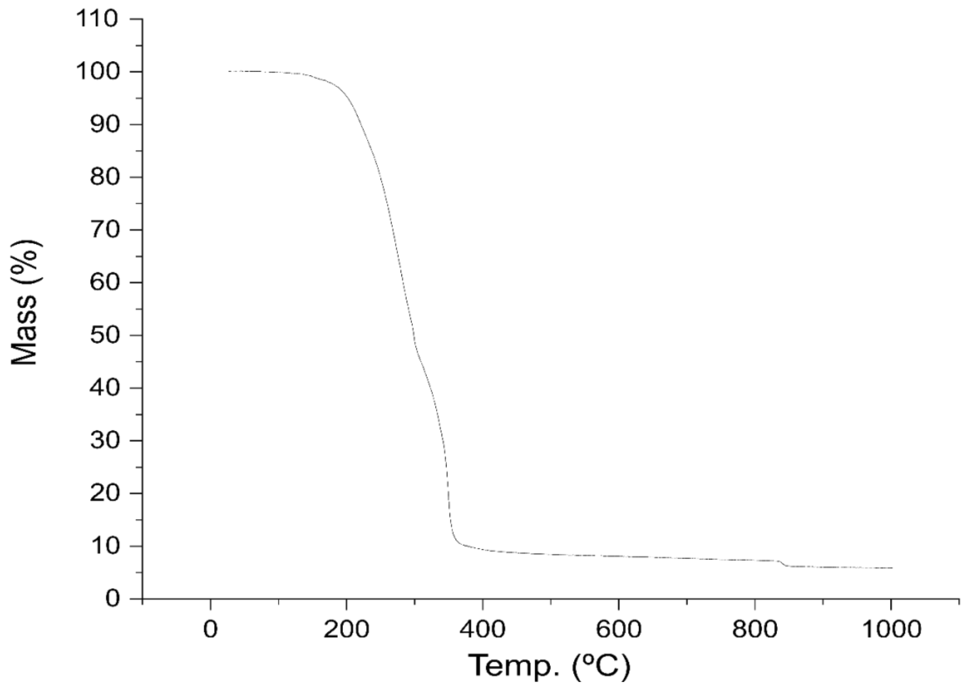

Figure S37. TG Curve of Complex **5C**

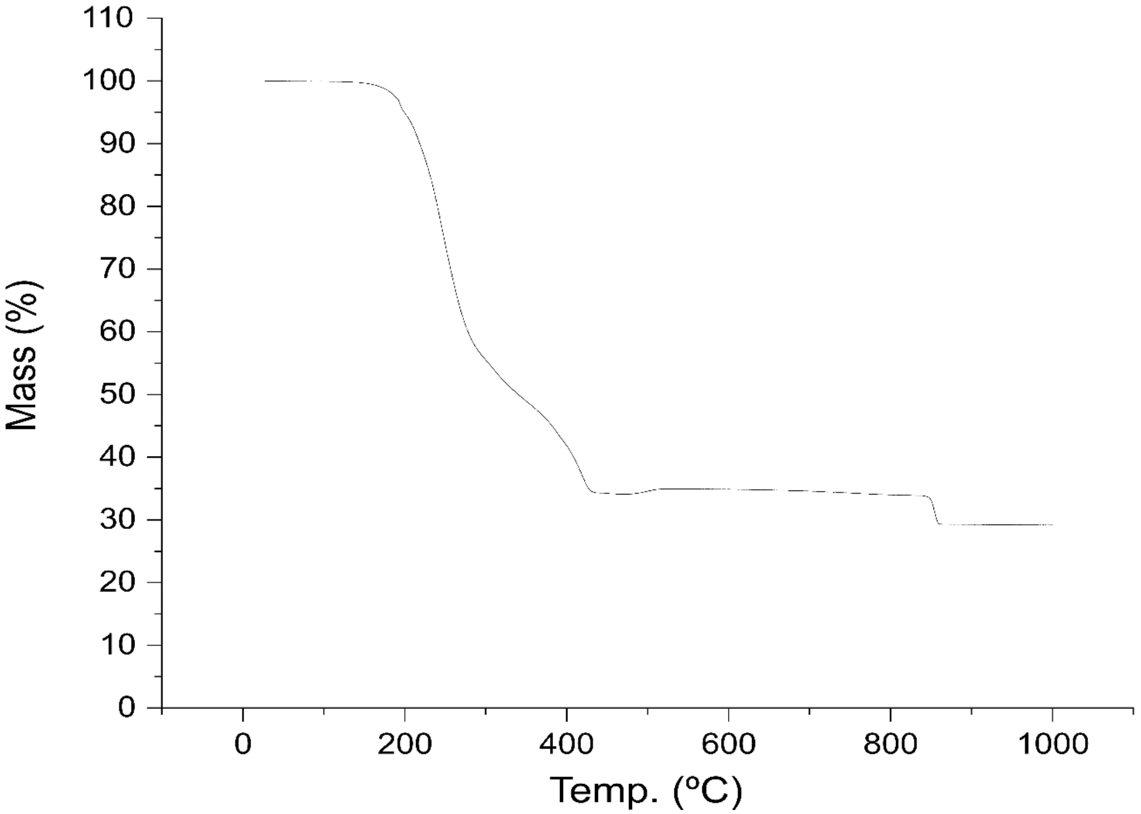

Figure S38. TG Curve of Complex **6C**

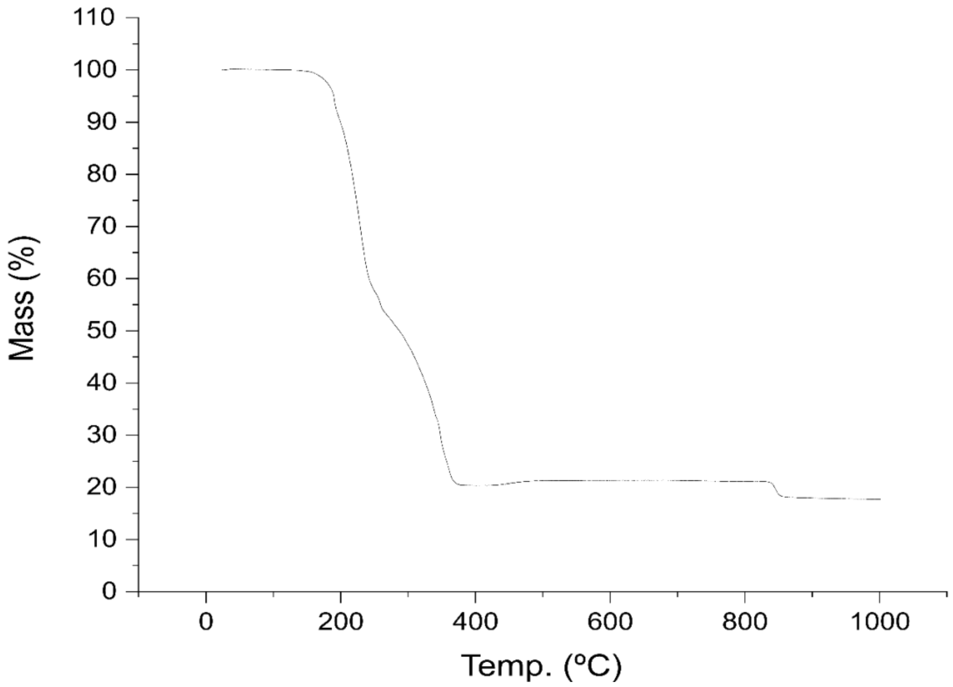

Figure S39. TG Curve of Complex **7C**

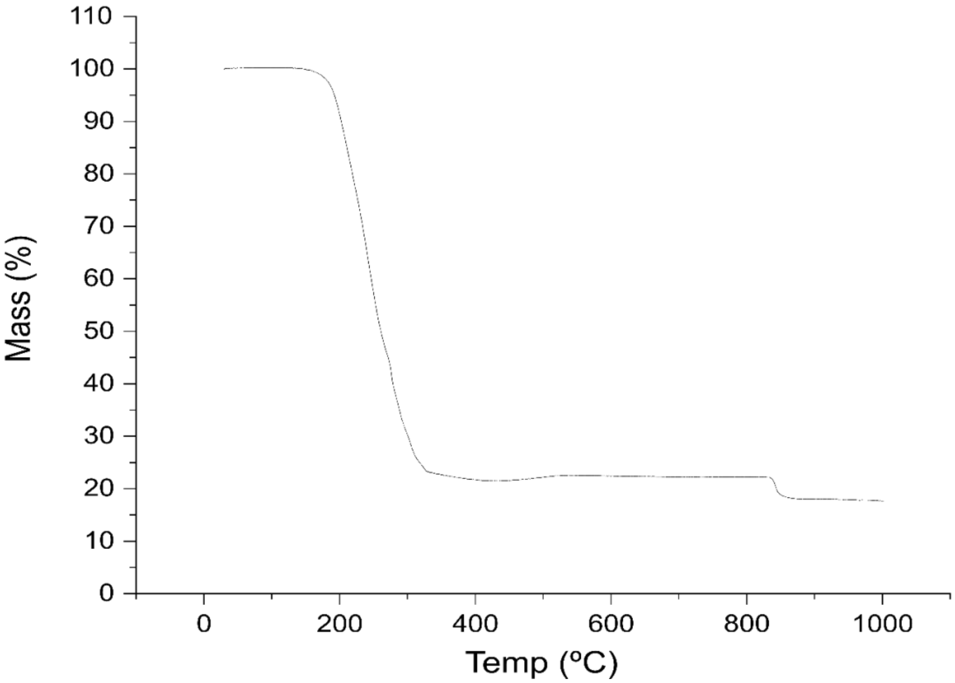

Figure S40. TG Curve of Complex **8C**

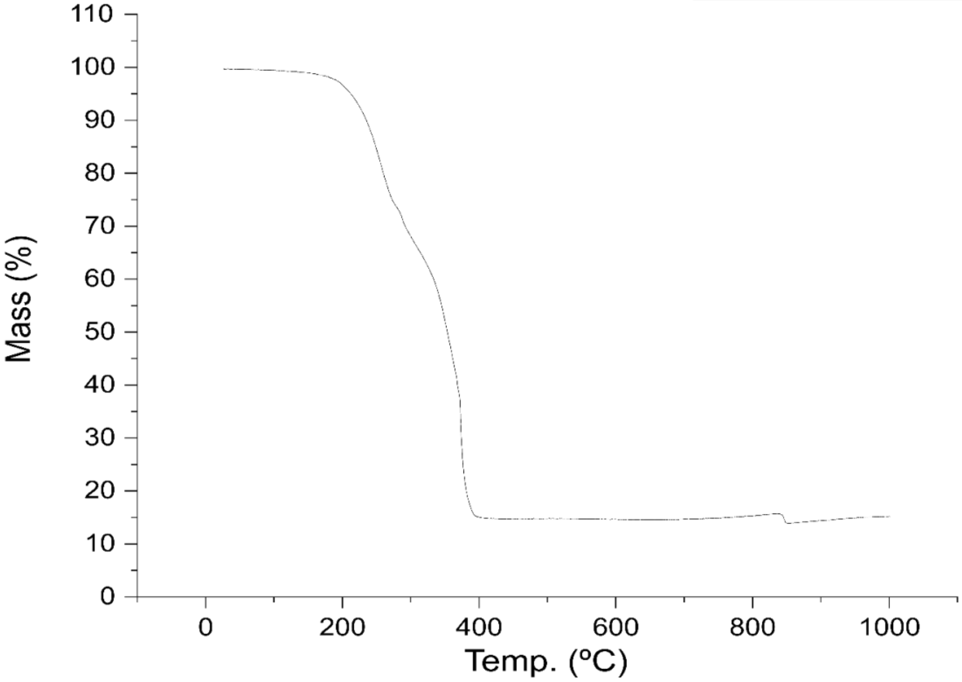

1.5 Massas Characterization by Mass Spectrometry

Figure S41. Comparison between the experimental (A) and theoretical (B) mass spectra of the complex **1C**

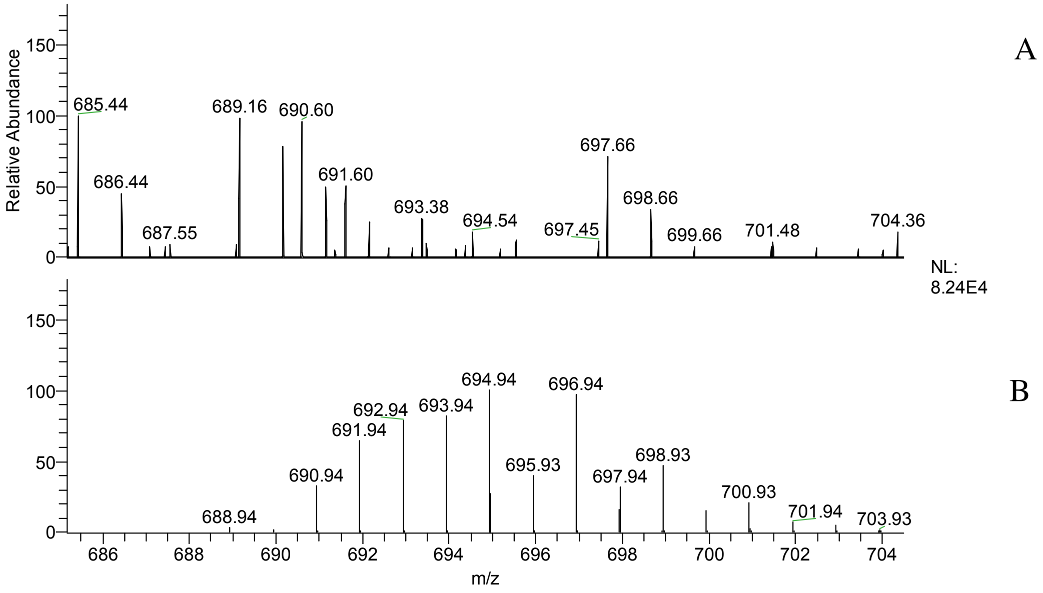

Figure S42. Comparison between the experimental (A) and theoretical (B) mass spectra of the complex **2C**

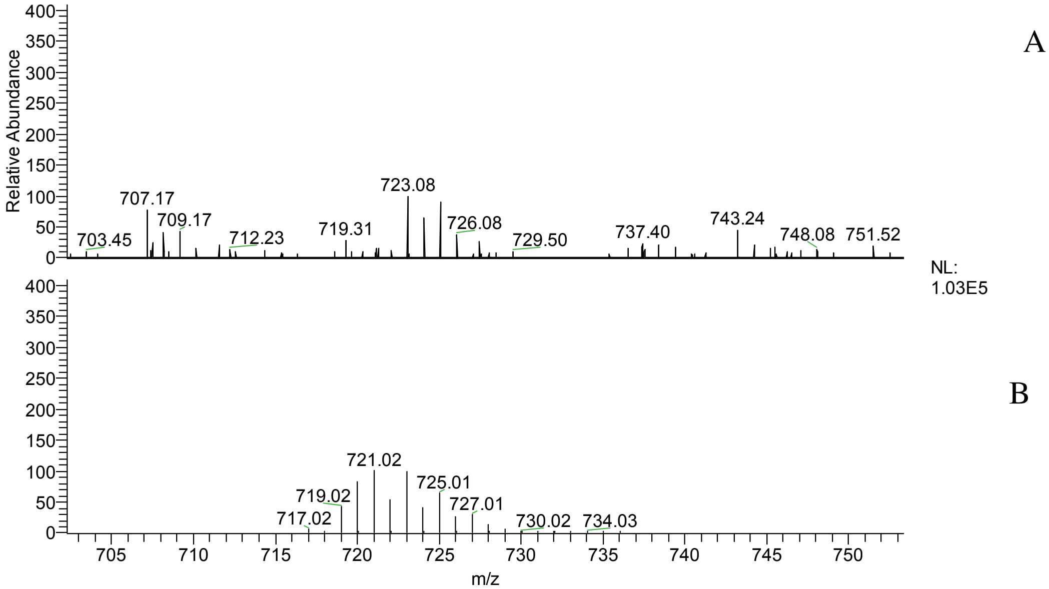

Figure S43. Comparison between the experimental (A) and theoretical (B) mass spectra of the complex **3C**

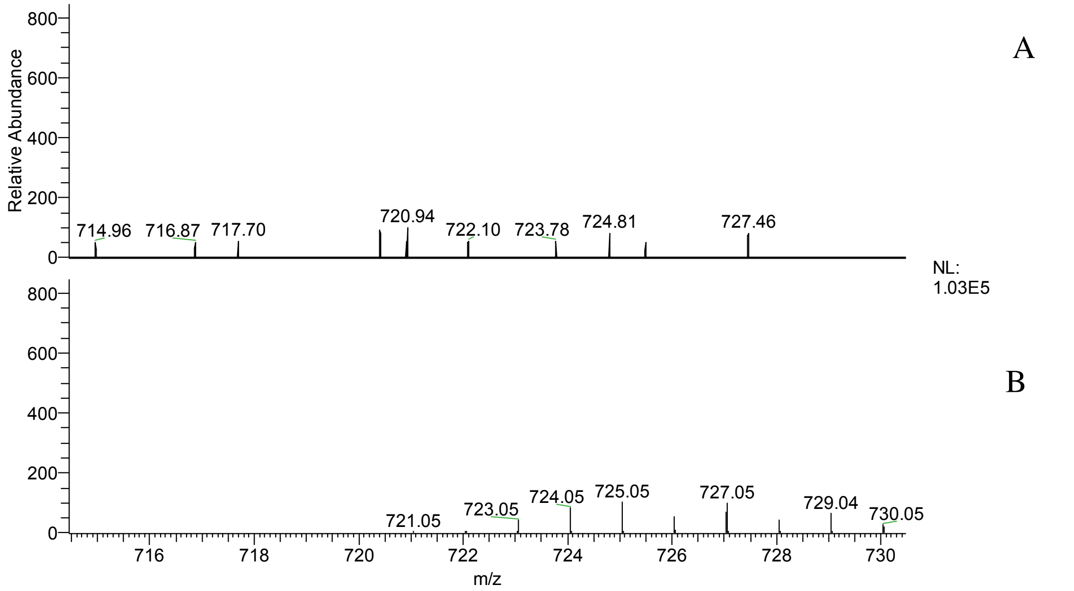

Figure S44. Comparison between the experimental (A) and theoretical (B) mass spectra of the complex **4C**

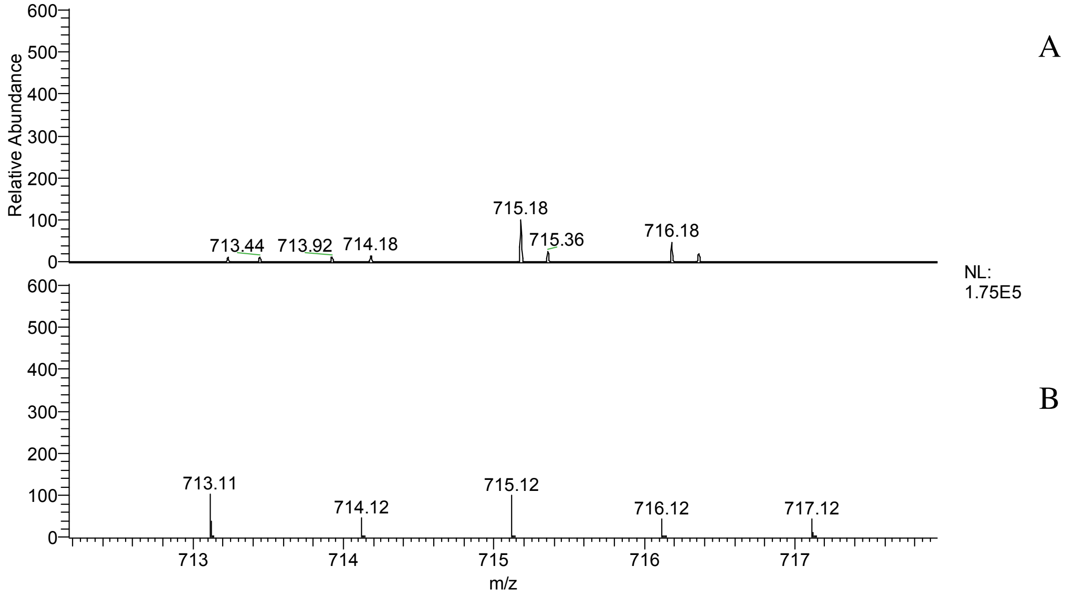

Figure S45. Comparison between the experimental (A) and theoretical (B) mass spectra of the complex **5C**

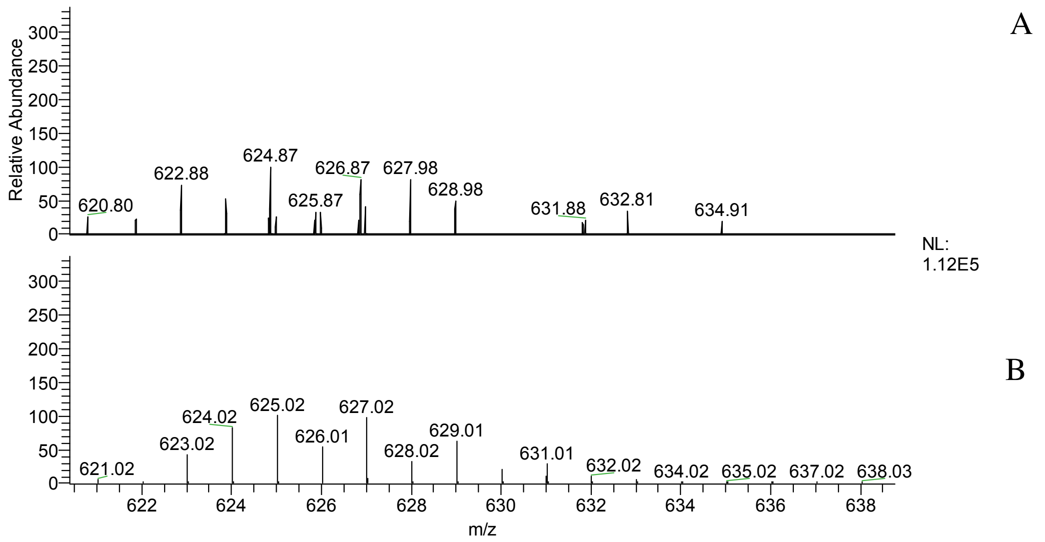

Figure S46. Comparison between the experimental (A) and theoretical (B) mass spectra of the complex **6C**

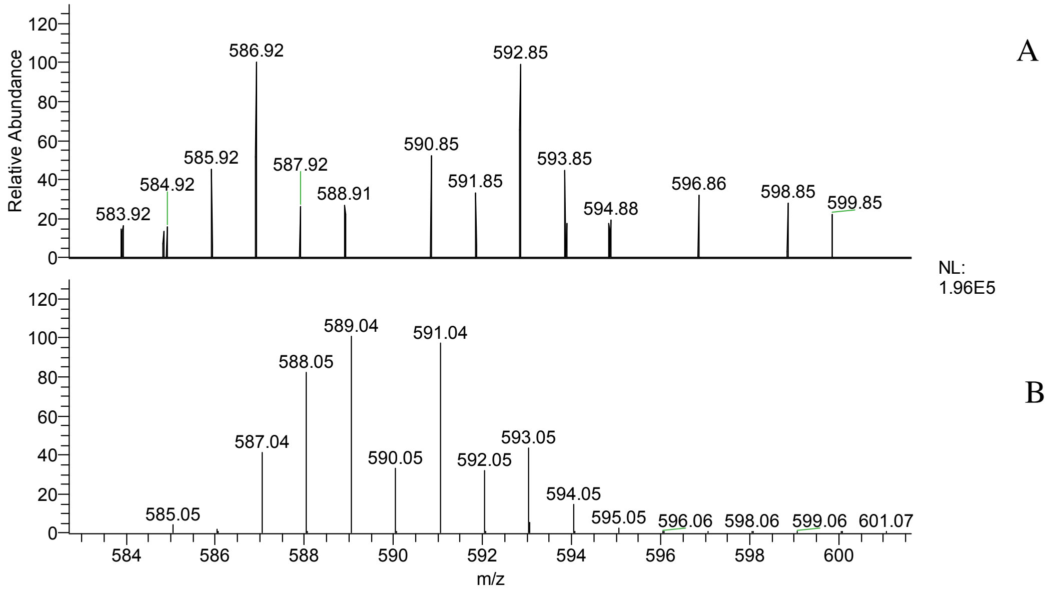

Figure S47. Comparison between the experimental (A) and theoretical (B) mass spectra of the complex **7C**

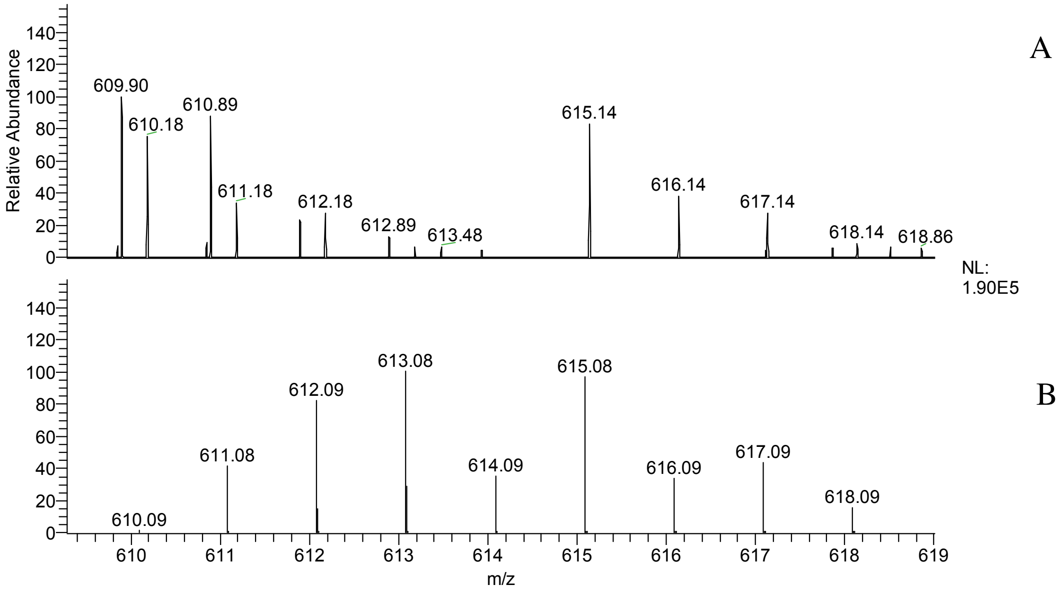

Figure S48. Comparison between the experimental (A) and theoretical (B) mass spectra of the complex **8C**

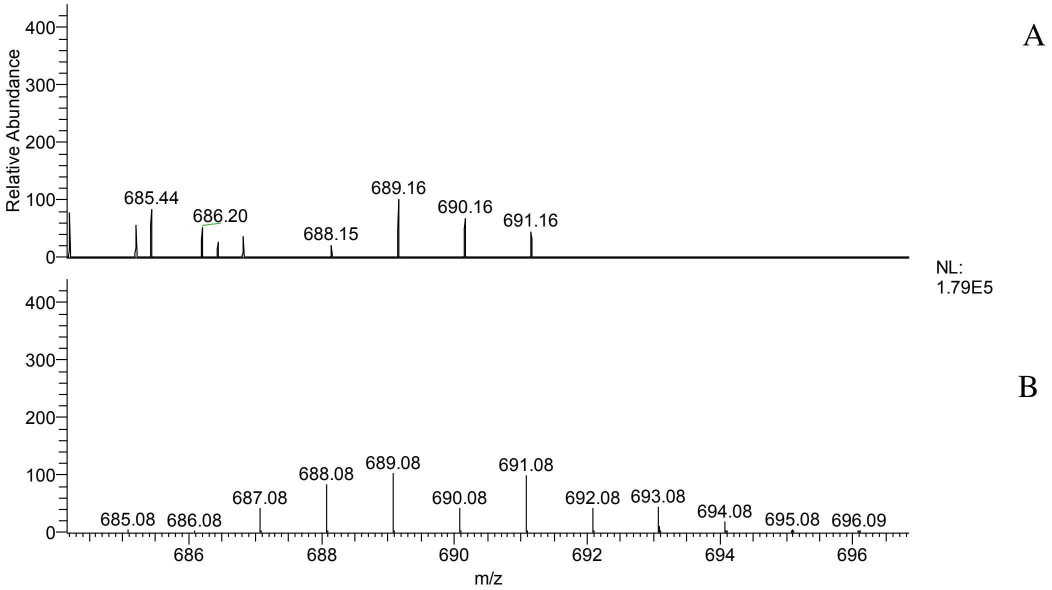

1.6 Thermogravimetric calculations of the complexes

1.6.1 Complex 1C – $C_{30}H_{22}Cl_4O_4Pd$

$$M_{9C} = 694.73 g \cdot mol^{-1}$$

The residue obtained by TGA was 10.8%, which implies:

$$\%\Delta m_{exp} = 100 - 10.8 = 89.2\%$$

If all the metal remained as Pd^0, the theoretical fraction would be:

$$\%R = \frac{106.42}{694.73} \cdot 100 \approx 15.3\%$$

Difference between the experimental value (10.8%) and the theoretical value (~15.3%).

1.6.2 Complex 2C – $C_{38}H_{24}Cl_2O_4Pd$

$$M_{13C} = 721.93 g \cdot mol^{-1}$$

Experimental residue: 8.2%

$$\%\Delta m_{exp} = 100 - 8.2 = 91.8\%$$

Theoretically, if only Pd remained:

$$\%R = \frac{106.42}{721.93} \cdot 100 \approx 14.7\%$$

Difference between the experimental value (8.2%) and the theoretical value (~14.7%).

1.6.3 Complex 3C – $C_{38}H_{28}Cl_2O_4Pd$

$$M_{11C} = 725.95 g \cdot mol^{-1}$$

Experimental residue: 8.3%

$$\%\Delta m_{exp} = 100 - 8.3 = 91.7\%$$

Theoretically, if the residue were only Pd:

$$\%R = \frac{106.42}{725.95} \cdot 100 \approx 14.6\%$$

Difference between the experimental value (8.3%) and the theoretical value (~14.6%).

1.6.4 Complex 4C – $C_{40}H_{30}O_6Pd$

$$M_{14C} = 713.08 g \cdot mol^{-1}$$

Experimental residue: 5.9%

$$\%\Delta m_{exp} = 100 - 5.9 = 94.1\%$$

Theoretically, if only Pd remained:

$$\%R = \frac{106.42}{713.08} \cdot 100 \approx 14.9\%$$

Difference between the experimental value (5.9%) and the theoretical value (~14.9%).

1.6.5 Complex 5C – $C_{30}H_{24}Cl_2O_4Pd$

$$M_{7C} = 625.84 g \cdot mol^{-1}$$

According to TGA, the final residue corresponded to 29.2% of the initial mass, implying a total experimental mass loss of:

$$\%\Delta m_{exp} = 100 - 29.2 = 70.8\%$$

Based on the thermal interpretation, the residue was assigned to PdO + 5C. The molar mass of this residue is:

$$M_{PdO} = 106.42 + 16 = 122.42 g \cdot mol^{-1}$$

$$M_{PdO+5C} = 122.42 + 5 \cdot (12) = 182.42 g \cdot mol^{-1}$$

The theoretical residue fraction is then:

$$\%R = \frac{182.42}{625.84} \cdot 100 \approx 29.2\%$$

1.6.6 Complex 6C – $C_{30}H_{20}F_2O_4Pd$

$$M_{8C} = 588.91 g \cdot mol^{-1}$$

TGA indicated a final residue of 17.8%; therefore:

$$\%\Delta m_{exp} = 100 - 17.8 = 82.2\%$$

Assuming the residue is metallic palladium (Pd^0), the theoretical Pd fraction is:

$$\%Pd_{calc} = \frac{106.42}{588.89} \cdot 100 \approx 18.1\%$$

1.6.7 Complex 8C – $C_{38}H_{24}F_2O_4Pd$

$$M_{12C} = 689.01 g \cdot mol^{-1}$$

Experimental residue: 15.2%

$$\%\Delta m_{exp} = 100 - 15.2 = 84.8\%$$

Theoretical (metallic Pd):

$$\%Pd_{calc} = \frac{106.42}{689.01} \cdot 100 \approx 15.4\%$$

1.7 Elemental analysis (CHN)

Table S1. Elemental (CHN) Analysis Data for Palladium(II) Complexes

Sample	Carbon (%) experimental	Carbon (%) theoretical	Hydrogen (%) experimental	Hydrogen (%) theoretical	Nitrogen (%)
**1C**	64.506	51.87	3.574	3.19	0
**2C**	64.588	63.22	3.764	3.35	0
**3C**	68.893	62.87	3.831	3.89	0
**4C**	70.743	67.37	4.074	4.27	0
**5C**	55.219	57.57	3.056	3.87	0
**6C**	60.017	61.19	3.159	3.42	0
**7C**	72.153	62.70	5.30	4.28	0
**8C**	66.102	66.24	3.437	3.51	0

Note: Elemental analysis values may deviate from theoretical values due to residual solvent molecules and the presence of heavy atoms (Pd, Cl, and F), which significantly influence the relative percentages of light elements in the combustion analysis.

1.8 Cellular Viability Response to Palladium Salts Dissolved in DMSO

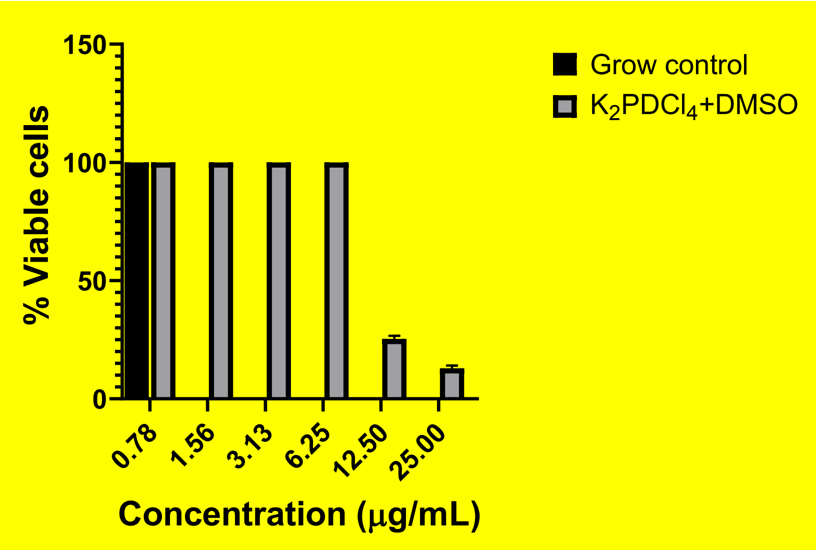

1.9 Molecular Docking

Figure S49. Molecular docking complex **1C**

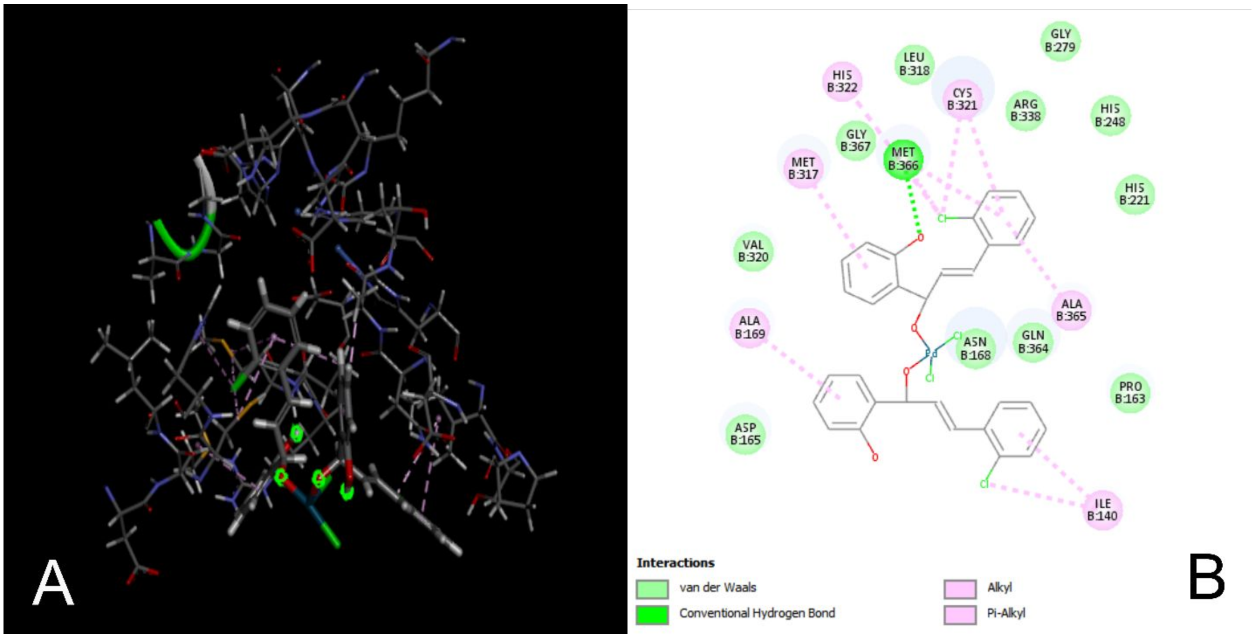

Note:(A) Stick diagram of the urease active site, with the main ligand interactions and (B) 2D diagram of the urease enzyme active site, with the main interactions.

Figure S50. Molecular docking complex **2C**

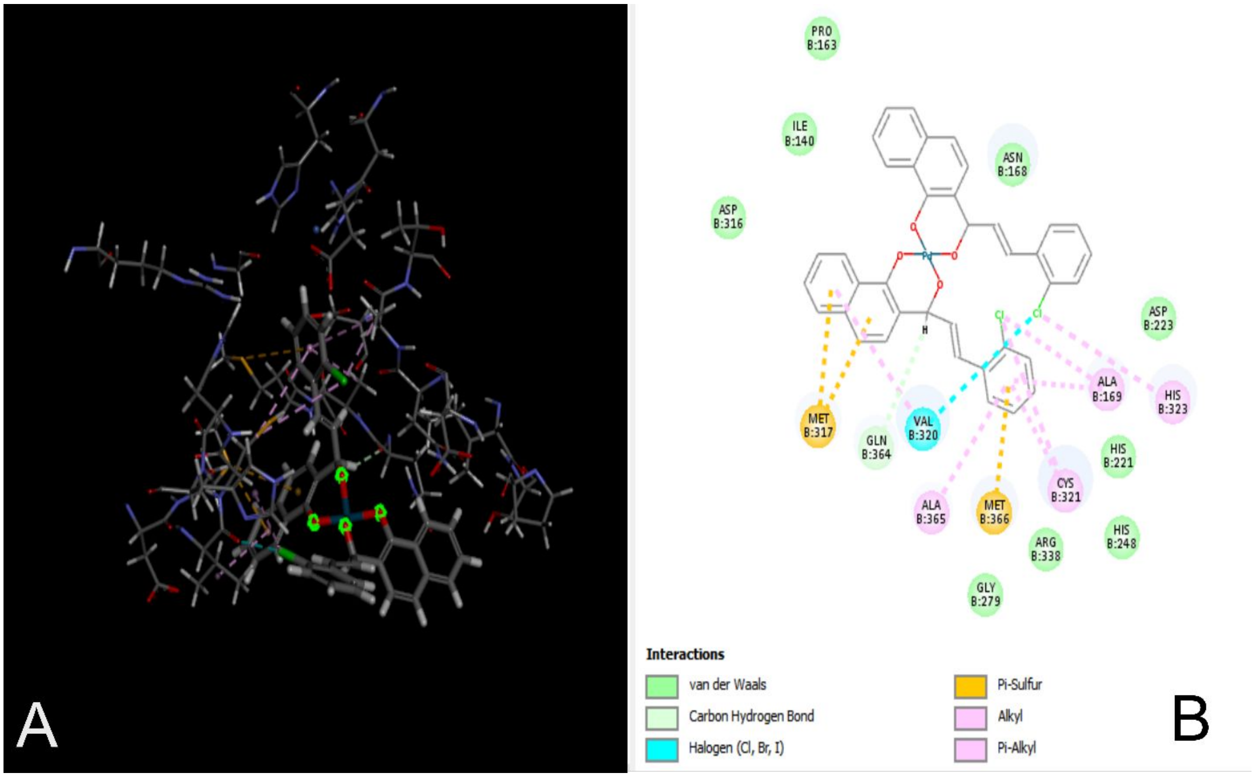

Note:(A) Stick diagram of the urease active site, with the main ligand interactions and (B) 2D diagram of the urease enzyme active site, with the main interactions.

Figure S51. Molecular docking complex **3C**

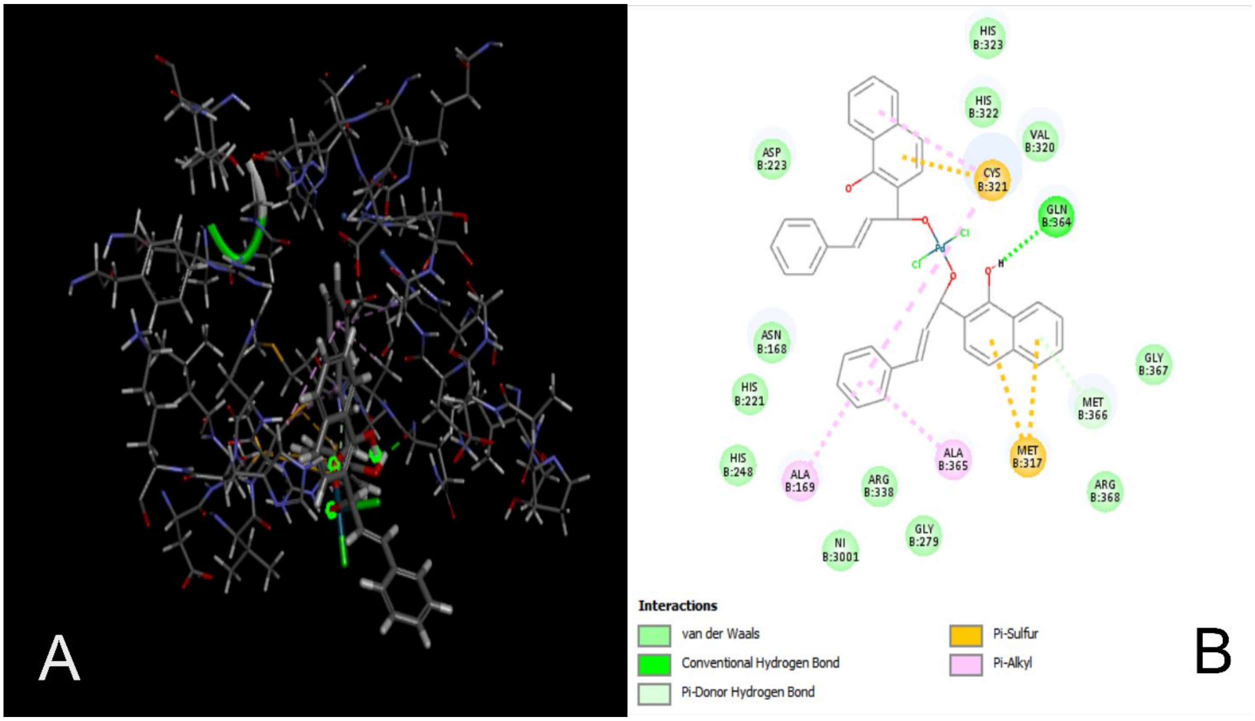

Note:(A) Stick diagram of the urease active site, with the main ligand interactions and (B) 2D diagram of the urease enzyme active site, with the main interactions.

Figure S52. Molecular docking complex **4C**

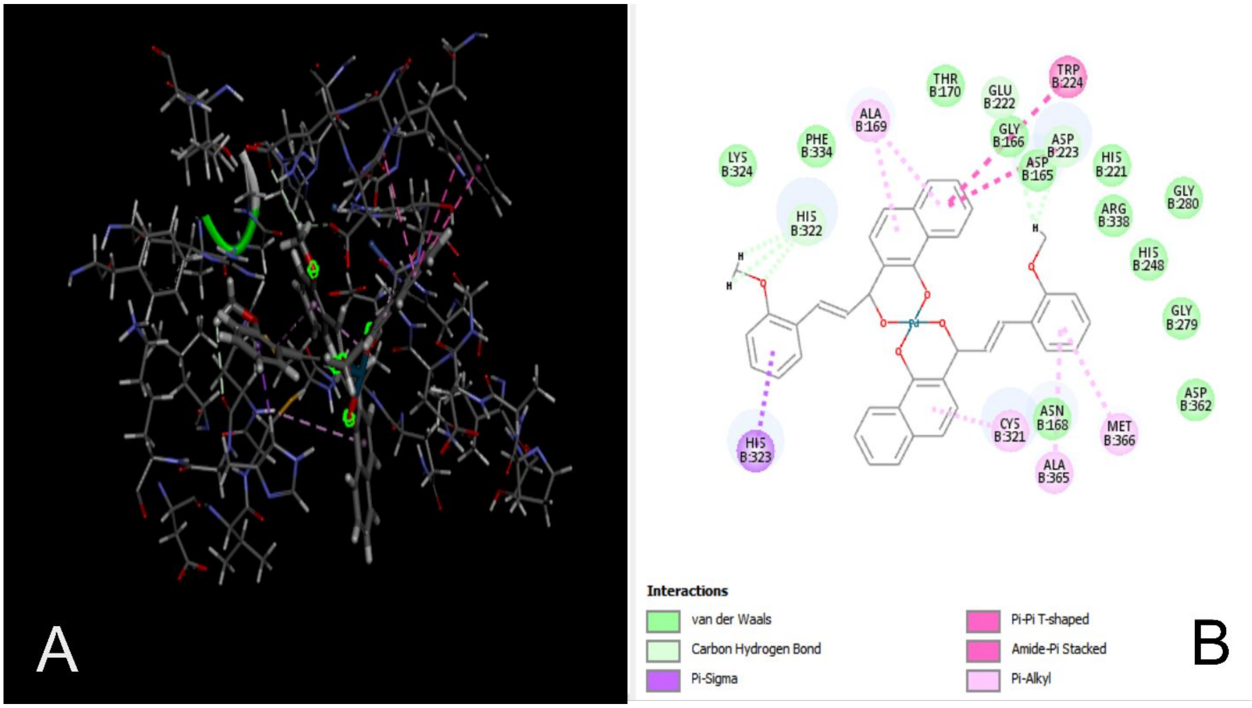

Note:(A) Stick diagram of the urease active site, with the main ligand interactions and (B) 2D diagram of the urease enzyme active site, with the main interactions.

Figure S53. Molecular docking complex **5C**

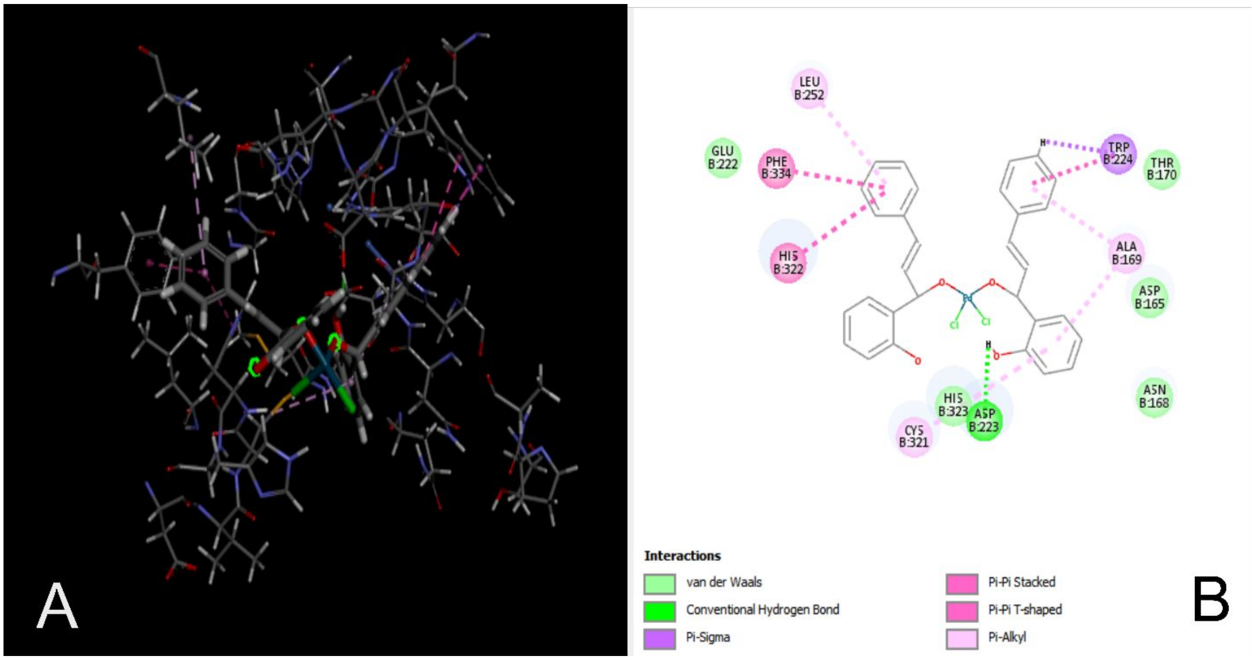

Note:(A) Stick diagram of the urease active site, with the main ligand interactions and (B) 2D diagram of the urease enzyme active site, with the main interactions.

Figure S54. Molecular docking complex **6C**

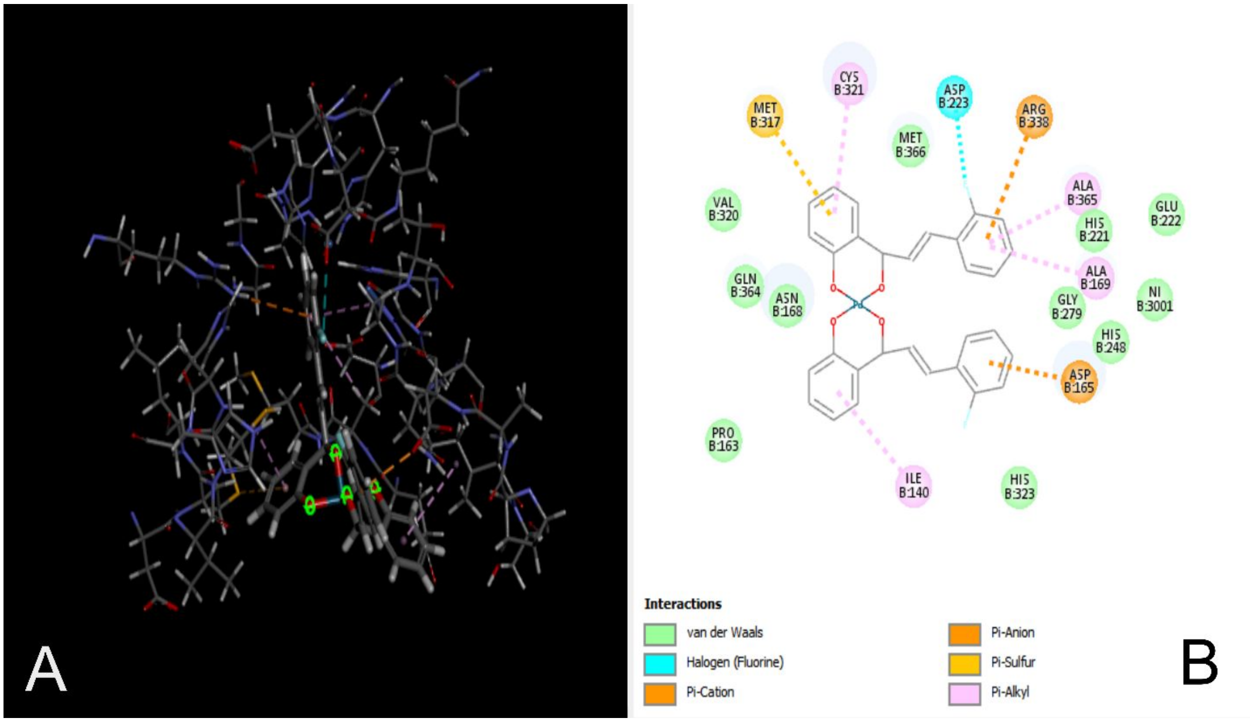

Note:(A) Stick diagram of the urease active site, with the main ligand interactions and (B) 2D diagram of the urease enzyme active site, with the main interactions.

Figure S55. Molecular docking complex **7C**

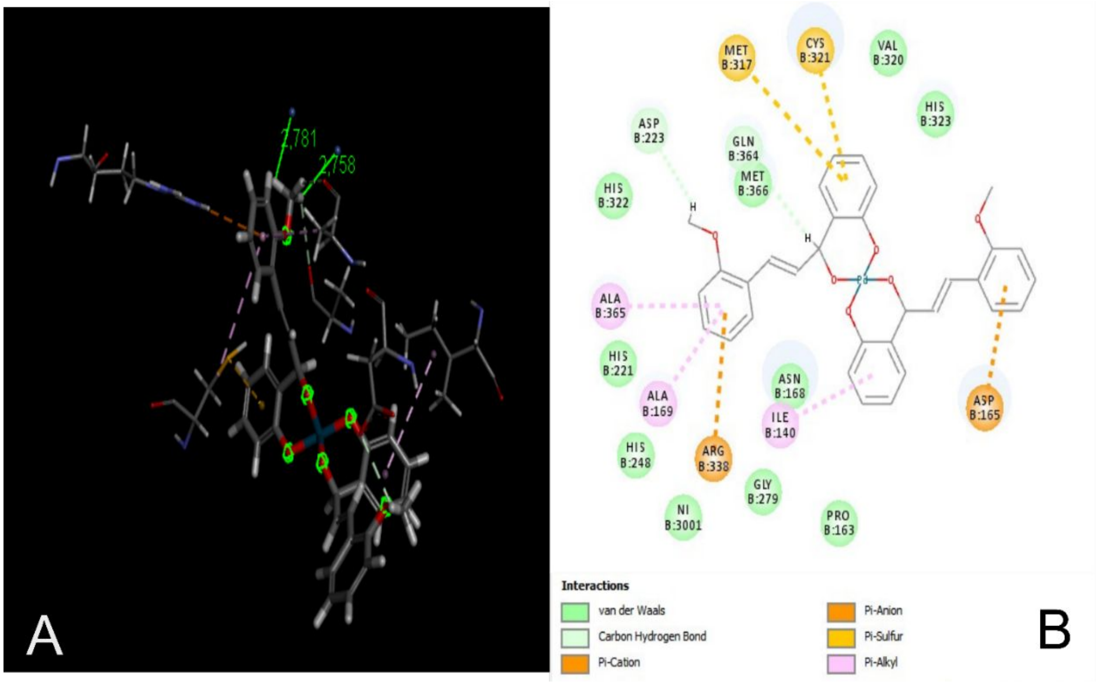

Note:(A) Stick diagram of the urease active site, with the main ligand interactions and (B) 2D diagram of the urease enzyme active site, with the main interactions.

Figure S56. Molecular docking complex **8C**

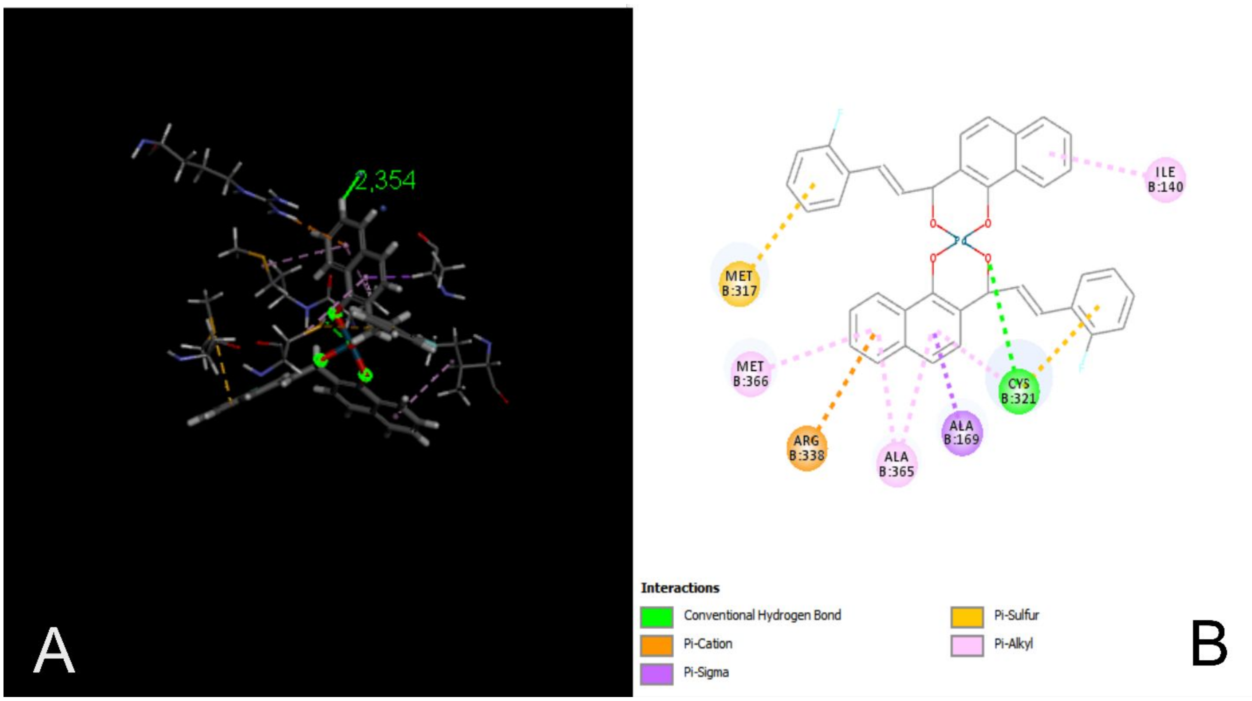

Note:(A) Stick diagram of the urease active site, with the main ligand interactions and (B) 2D diagram of the urease enzyme active site, with the main interactions.

Figure S57. Molecular docking chalcone **6**

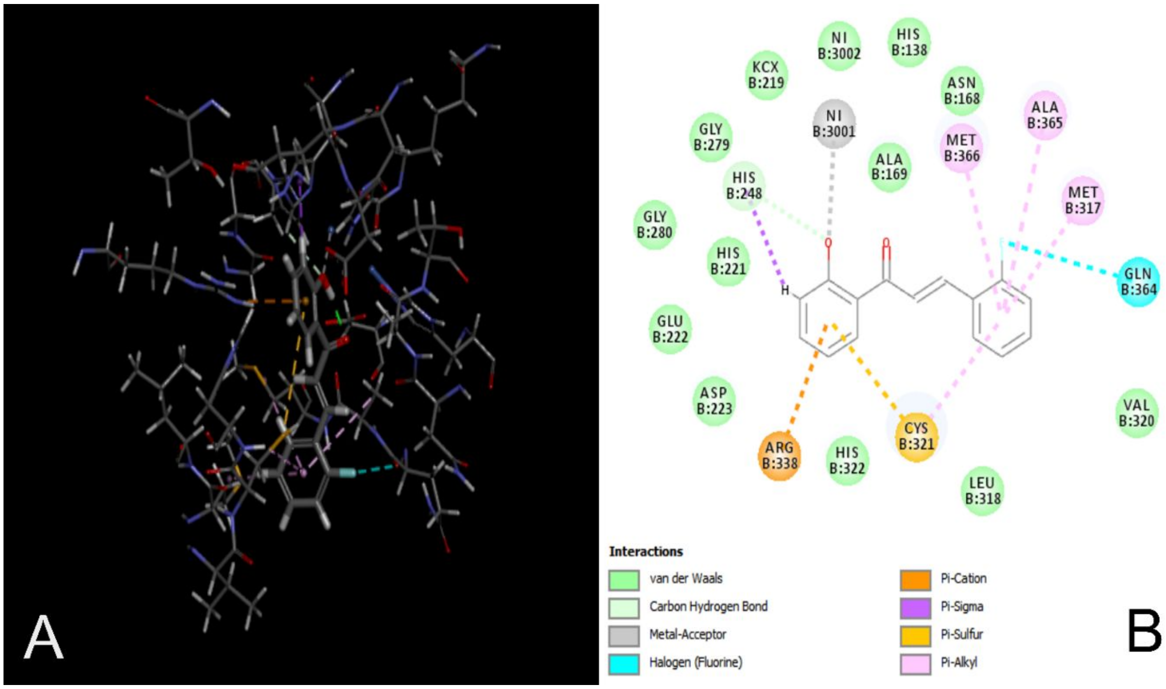

Note:(A) Stick diagram of the urease active site, with the main ligand interactions and (B) 2D diagram of the urease enzyme active site, with the main interactions.
